# Supplementary material for: Stable species boundaries despite ten million years of hybridization in tropical eels
Source: Nat Commun. 2020 Mar 18;11:1433. doi: 10.1038/s41467-020-15099-x (PMC7080837; doi:10.1038/s41467-020-15099-x)
Supplement: Supplementary file 1 — Supplementary Information [file 41467_2020_15099_MOESM1_ESM.pdf]

Supplementary Information

Stable Species Boundaries Despite Ten Million Years of Hybridization in Tropical Eels

Barth, Gubili, Matschiner et al.

Supplementary Notes

Supplementary Note 1: Contrasting patterns of within-species genomic variation.
Our extensive sampling scheme of tropical eels permitted detailed analyses of genomic variation within *A. marmorata*, *A. megastoma* and *A. obscura*, as we sampled each of the three species at multiple sites throughout their geographic distribution (Fig. 1a; Supplementary Table 1). These analyses were based on a dataset of 155,896 RAD-sequencing derived single-nucleotide polymorphisms (SNPs), partitioned according to species and subsequently filtered to exclude invariant sites (minor allele count > 2) and missing data ($> 20\%$; Supplementary Figure 1). Using these partitioned datasets, principal-component analysis (PCA) of genomic variation was performed with smartpca in EIGENSOFT v.6.0.1 [1], including the function "lsqproject" to account for missing data. For *A. marmorata*, PCA separated four populations present in the western Indian Ocean (South Africa, Reunion, Mayotte), in Indonesia (Java), the South China Sea (Philippines and Taiwan), and the western South Pacific (Bougainville Island, Solomon Islands, Vanuatu, New Caledonia, Samoa, and American Samoa). The latter three, however, were only discernible on the second principal-component axis (explaining 2.2% of genetic variation), along which the individuals from Java appeared intermediate between those from the western Indian Ocean and the western South Pacific (Supplementary Figure 4a). Our results are thus consistent with divergence among Indian and Pacific ocean populations [2–5] and with the region of Java representing a contact zone between those populations [5]. Conversely, no population structure was detected in either *A. megastoma* or *A. obscura* (Supplementary Figures 4c-f), supporting the hypothesized single spawning area for the two species in the western South Pacific [6, 7].

Supplementary Note 2: Double-digest restriction-site associated DNA (ddRAD) sequencing.
Following Peterson et al.[8], 20 units of EcoRI-HF (New England Biolabs) and 20 units of MspI (New England Biolabs) were used to digest 400 ng of genomic DNA per sample in a 37 °C incubation for 8 hours. Digests were purified with homemade paramagnetic carboxyl-modified beads (Sera-Mag, Fisher Scientific) [9]. Moreover, samples were randomly placed in PCR plates, ensuring a wide coverage of geographic sampling locations per plate during library preparation. T4 DNA ligase (New England Biolabs) was applied to ligate 100 ng of each digested DNA fragment to a EcorRI-specific P1 adapter that contained a 5-bp barcode and the MspI-specific P2 adapter in room temperature, followed by an enzyme heat-kill at 65 °C for 10 min. Twenty-four unique barcodes were used so that the ligated DNA fragments from 24 individuals could be pooled (according to one index) to form a single ddRAD-seq library. Ligations were cleaned with homemade paramagnetic carboxyl-modified beads. Fragments in the range of 300-400 bp were selected using AMPure XP beads (Agilent Technologies) and were subsequently amplified by 12 rounds of PCR with the following conditions: 98 °C for 60 s; 12 cycles of 98 °C for 10 s, 60 °C for 30 s, 72 °C for 30 s; 72 °C for 10 min using Q5® High Fidelity polymerase (New England Biolabs), and the Illumina sequencing primers (PCR Primer 1 and Index added PCR Primer 2; four unique 6-bp multiplexing indices were used). In total, six separate PCR reactions of the same index were amplified, pooled and cleaned with homemade paramagnetic carboxyl-modified beads. Library quality was assessed on a TapeStation

2200 (Agilent Technologies) to confirm fragment recovery on the selected range and was quantified using a Qubit Fluorometer 2.0. To avoid index-hopping [10, 11], none of the samples shared both the P1 barcode and the multiplexing index in a given sequencing run. In total, 20 libraries, each containing 24 barcodes, were sent to Macrogen (Korea) for 100 bp paired-end Illumina HiSeq 4000 sequencing.

Supplementary Note 3: The reliability of published age estimates for the genus *Anguilla*.
We time calibrated the species tree of tropical eels according to age estimates reported by Jacobsen et al.[12] on the basis of mitochondrial genomes. In their study, Jacobsen et al.[12] used the earliest fossil records of the family Anguillidae, *Eoanguilla leptoptera* from Monte Bolca, Italy [13, 14], to constrain the divergence between Anguillidae and Serrivomeridae to 55-50 Ma. Given that the age of the Monte Bolca deposits is 49.4-49.1 Ma [15, 16], it is indeed likely that Anguillidae originated before 50 Ma. However, since fossils do not directly constrain maximum ages, the upper boundary of 55 Ma was arbitrarily specified by Jacobsen et al.[12], and the divergence times estimated in their study would likely be underestimated if Anguillidae in fact originated earlier than 55 Ma. Nevertheless, we consider the timeline proposed by Jacobsen et al.[12] plausible for the following reasons: (i) According to this timeline, European and American anguillid species (*A. anguilla* and *A. rostrata*) diverged from Indo-Pacific members of the genus around 10.8 Ma, which is consistent with the Messinian age (7.2-5.3 Ma) of the earliest fossils of the genus, known from the Gessoso Solififera Formation in Northern Italy [17]; (ii) the timeline is consistent with those of two other recent studies based on genome-wide data [18] and a massive taxon set [19], as the most recent common ancestor of *A. anguilla* and *A. japonica* (the only species pair included in all three studies) was estimated at 13.8 Ma by Jacobsen et al.[12], at 12.4 Ma by Musilova et al.[18], and at 12.9 Ma by Rabosky et al.[19].

Supplementary Note 4: Assessing the robustness of divergence-time estimates.
To test how robust the divergence-time estimates are to alternative phylogenetic positions of *A. interioris*, SNAPP analyses were repeated separately with two fixed topologies in which *A. interioris* is either the sister of *A. bicolor* and *A. obscura* or the sister to the clade formed by *A. marmorata*, *A. luzonensis*, *A. bicolor*, and *A. obscura*. Furthermore, to test the robustness of divergence-time estimates to introgression involving *A. luzonensis* and *A. interioris*, the analyses were repeated after excluding these two species.

Additionally, we compiled a multi-locus phylogenetic dataset based on genome assemblies of the five species *A. anguilla*, *A. japonica*, *A. marmorata*, *A. obscura*, and *A. megastoma* to estimate divergence times among *Anguilla* species independently of the timeline of Jacobsen et al.[12]. Of these five species, genome assemblies of *A. anguilla* (NCBI accession GCA_000695075 [20]) and *A. japonica* (NCBI accession GCA_000470695 [21]) were included in the large-scale phylogenomic analysis of Musilova et al.[18], in which their divergence was estimated at around 12.37 Ma. We thus extracted ortholog sequences, corresponding to the loci used by Musilova et al.[18], from the three new genome assemblies of *A. marmorata*, *A. obscura*, and *A. megastoma* (Supplementary Table 5),

and aligned these jointly with those of *A. anguilla* and *A. japonica*. Alignments were then filtered according to the protocol of Musilova et al.[18], excluding 10 of the 113 genes used by Musilova et al.[18] due to missing sequences. Alignments for the remaining 103 nuclear genes were concatenated and split into two separate partitions for first- and second-codon positions; third-codon positions were excluded. Together, these two partitions included 92,530 bp with 0.07% missing data. The concatenated alignment was used for phylogenetic analyses with the software BEAST 2 [22], time calibrating the phylogeny according to the timeline estimated by Musilova et al.[18]. Specifically, we used 12.37 Ma, the estimated age for the divergence of *A. anguilla* and *A. japonica*, as a constraint on the age of the most recent common ancestor of the five *Anguilla* species, after initial analyses suggested an position of *A. anguilla* outside of a clade formed by the other four species. The model used in this analysis included a GTR substitution-rate matrix [23], gamma-distributed among-site rate variation, a strict molecular clock, and the Yule process of species diversification [24]. The BEAST 2 analysis was performed with 10 million MCMC iterations. Convergence was again assessed with Tracer, and the posterior tree distribution was summarized in an MCC tree generated with TreeAnnotator.

As another alternative to the divergence-time estimates of Jacobsen et al.[12], we also implemented age constraints according to the timeline of Rabosky et al.[19]. The large-scale time-calibrated phylogeny of Rabosky et al.[19], based on molecular data for 11,638 ray-finned fishes and 139 fossil constraints, includes 20 species and subspecies of the genus *Anguilla* and places the earliest divergence within the genus, the separation of *A. australis*, at 21.57 Ma. As the genus was mostly (82%) represented by mitochondrial sequences in the dataset of Rabosky et al.[19], we extracted homologous mitochondrial sequences from the new genome assemblies of *A. marmorata*, *A. megastoma*, and *A. obscura* (Supplementary Table 5) using BLAST [25], and integrated these sequences with the mitochondrial data for *Anguilla* compiled by Rabosky et al.[19]. This integrated mitochondrial dataset was concatenated into a single alignment and used again for phylogenetic inference with BEAST 2. According to the phylogeny of Rabosky et al.[19], *A. australis* was constrained to be the outgroup of the other *Anguilla* species and the divergence of *A. australis* was fixed at 21.57 Ma. The model used in this analysis was identical to that used for nuclear data, except that the GTR substitution-rate matrix was replaced with an HKY matrix [26] because some GTR rate parameters appeared unidentifiable in preliminary analyses. The BEAST 2 analysis was again performed for 10 million MCMC iterations, convergence was assessed with Tracer, and a MCC summary tree was generated with TreeAnnotator.

Supplementary Note 5: Identification of genomic regions with potential structural rearrangements.

To identify structural rearrangements among eel genomes that could potentially be linked to cytonuclear incompatibilities, we performed whole-genome alignment with the program LASTZ v.1.04 [27]. This was done separately for the assemblies of *A. japonica*[21], *A. marmorata*, *A. megastoma*, and *A. obscura* that were all aligned to the *A. anguilla* reference genome assembly [28], after excluding scaffolds shorter than 100,000 bp from this reference assembly. To prepare all genome assemblies for whole-genome alignment, we first identified and soft-masked repetitive regions with both Tandem

Repeats Finder (TRF) v.4.07b [29] and RepeatMasker v.1.0.8 (http://www.repeatmasker.org). For TRF, we applied a matching weight of 2, a mismatching penalty of 7, an indel penalty of 7, a match probability of 80, and indel probability of 10, and limited the report to alignments with a minimum score of 50 and a maximum period size of 2,000. RepeatMasker was run with default settings, using a repeat library generated ab initio with the associated tool RepeatModeler. Various Genome Browser and BLAT utilities (http://hgdownload.soe.ucsc.edu/admin/exe) [30] were used for format conversions. Subsequent to alignment with LASTZ, the tool single_cov2 from the MULTIZ-TBA program package v.012109 [31] was applied to remove overlapping regions from aligned blocks in the whole-genome alignments.

We then investigated each of the four pairwise whole-genome alignments for signals of structural genomic rearrangements between *A. anguilla* and the other eel species, focusing on alignment blocks with a minimum LASTZ score of 50,000 and a minimum length of 1,000 bp. Specifically, when the same two scaffolds appeared in multiple alignment blocks, we recorded a potential inversion if some of these alignment blocks differed in their orientation and a potential transposition if the aligned fragments from the two scaffolds did not appear in the same order on those scaffolds. For each potential rearrangment, the start and end positions of adjacent alignment blocks were used to localize the rearrangement within a region of the *A. anguilla* reference assembly. Out of 378 potential rearrangements, we excluded 110 that could not be localized more precisely than within 10 kb from further analyses. We unified the set of potential rearrangement regions across the four pairwise whole-genome alignments by merging overlapping regions on the *A. anguilla* reference assembly. For example, if a potential rearrangement between *A. marmorata* and *A. anguilla* was found between positions 1 kb and 3 kb on a given *A. anguilla* scaffold and a potential rearrangement between *A. megastoma* and *A. anguilla* was detected between positions 2 kb and 4 kb on the same *A. anguilla* scaffold, we assumed that a rearrangements may have occured at the same position in both pairs, between 2 kb and 3 kb of the scaffold. This produced a set of 255 regions for which one or more of the four whole-genome alignments supported the presence of a rearrangement.

Supplementary Note 6: Verification of the presence or absence of rearrangements among eel genomes.

For each of the 255 regions with potential rearrangements (see Supplementary Note 5), we inspected once again each of the original four pairwise whole-genome alignments with more permissive filtering thresholds as before to determine the presence or absence of the rearrangement between the species pair. Specifically, we first determined, for each region and each pairwise whole-genome alignment, the scaffolds in the alignment blocks closest to the start and the end of the region, and, if any, the scaffolds in alignment blocks completely within the region. If a single alignment block or multiple closely spaced (< 500 bp) alignment blocks (with sequences in identical orientation and order) spanned the entire region, the rearrangement was recorded to be absent between the species pair. If multiple more distantly spaced (≥ 500 bp) alignment blocks were localized within the region and included the same non-reference scaffold, an inversion was recorded if the alignment blocks differed in their orientation, and a transposition was recorded if the order of aligned fragments differed between the reference and the non-reference scaffold. When different non-reference scaffolds were

included in alignment blocks covering the region, with LASTZ alignment scores greater than 50,000 and alignment lengths greater than 500 bp, we recorded that the presence of the rearrangement was unknown in the given species pair.

For each region, we then determined whether it was within or close to a coding sequence on the *A. anguilla* reference genome assembly according to gene prediction with AUGUSTUS v.3.3.3 [32] (see Supplementary Note 7). If a coding sequence overlapped with the region or was located within 2,000 bp upstream or downstream of it, we used BLASTP [25] searches to compare the translated coding sequences to the zebrafish (*Danio rerio*) proteome (assembly version GRCz11; NCBI accession GCA_000002035.4 [33]) to identify homologous proteins. We further generated dot plots for each pair of scaffolds with a putative rearrangement, and visually assessed whether or not the resulting 874 dot plots confirmed the presence of rearrangements. A list of all detected potential rearrangements, with or without visual confirmation, is provided in Supplementary Table 11. Nine regions on eight different *A. anguilla* scaffolds were visually confirmed to contain rearrangements that were species-specific to either *A. marmorata*, *A. megastoma*, or *A. obscura*; dot plots for these regions are shown in Supplementary Figure 21.

As errors in the assemblies of *A. marmorata*, *A. megastoma*, and *A. obscura* could potentially generate the same patterns as true rearrangements in dot plots, we further investigated the WGS reads of these species mapped to either the *A. anguilla* reference genome assembly and the species-specific assembly. For true rearrangements, we expected a rather homogenous depth of reads mapped to the species-specific assembly but short regions with a depth approaching zero in the reads mapped to the *A. anguilla* assembly. We also expected reads without proper pairing at the boundaries of true inversions when reads are mapped to the *A. anguilla* assembly, but not when these are mapped to the species-specific assembly. In contrast, we expected putative errors in the species-specific assemblies to produce regions with a read depth close to zero as well as large proportions of reads without proper pairing when reads are mapped to species-specific assemblies but not when these are mapped to the *A. anguilla* assembly. We mapped reads to the *A. anguilla* and species-specific assemblies with BWA MEM v.0.7.17 [34] and subsequently used SAMtools v.1.3 to remove all unmapped reads, reads with a mapping quality below 40, reads without mapped mates, duplicate reads, and reads that were placed in supplementary alignments (SAM flag 3596). From the remaining set of high-quality reads, we identified reads without proper pairing as those that had a mate on the same strand or within unexpected distance (SAM flags 2 and 48).

From the resulting distributions of reads in putative recombination regions (also shown in Supplementary Figure 21), we conclude that species-specific inversions are present in *A. obscura* on scaffold scf7180011634263 (Supplementary Figure 21a) and in *A. megastoma* on scaffold scf7180010922797 (Supplementary Figure 21d). In addition, we find evidence for the heterozygous presence of inversions in *A. obscura* on scaffold scf7180011662322 (Supplementary Figure 21g and Supplementary Figure 22a) and in *A. megastoma* on scaffold scf7180010919884 (Supplementary Figure 21 and Supplementary Figure 22b). The latter of these occurs in a region that is homologous to exon 35 of the *myhc4* gene in zebrafish (*Danio rerio*) (Supplementary Figure 22b). The inversion may thus affect the protein encoded by this gene, myosin heavy chain, in *A. megastoma*.

Supplementary Note 7: De novo gene prediction for the *A. anguilla* reference genome assembly. We performed de novo gene prediction for the *A. anguilla* reference genome [28] using the AUGUSTUS v.3.3.3 web server (http://bioinf.uni-greifswald.de/webaugustus/prediction) [32]. We applied the zebrafish (*Danio rerio*) training set, excluded the prediction of untranslated regions and alternative transcripts, and set AUGUSTUS to report genes on both strands while considering potential conflict between the two strands.

Supplementary Figures

Supplementary Figure 1: Molecular datasets used in this study.

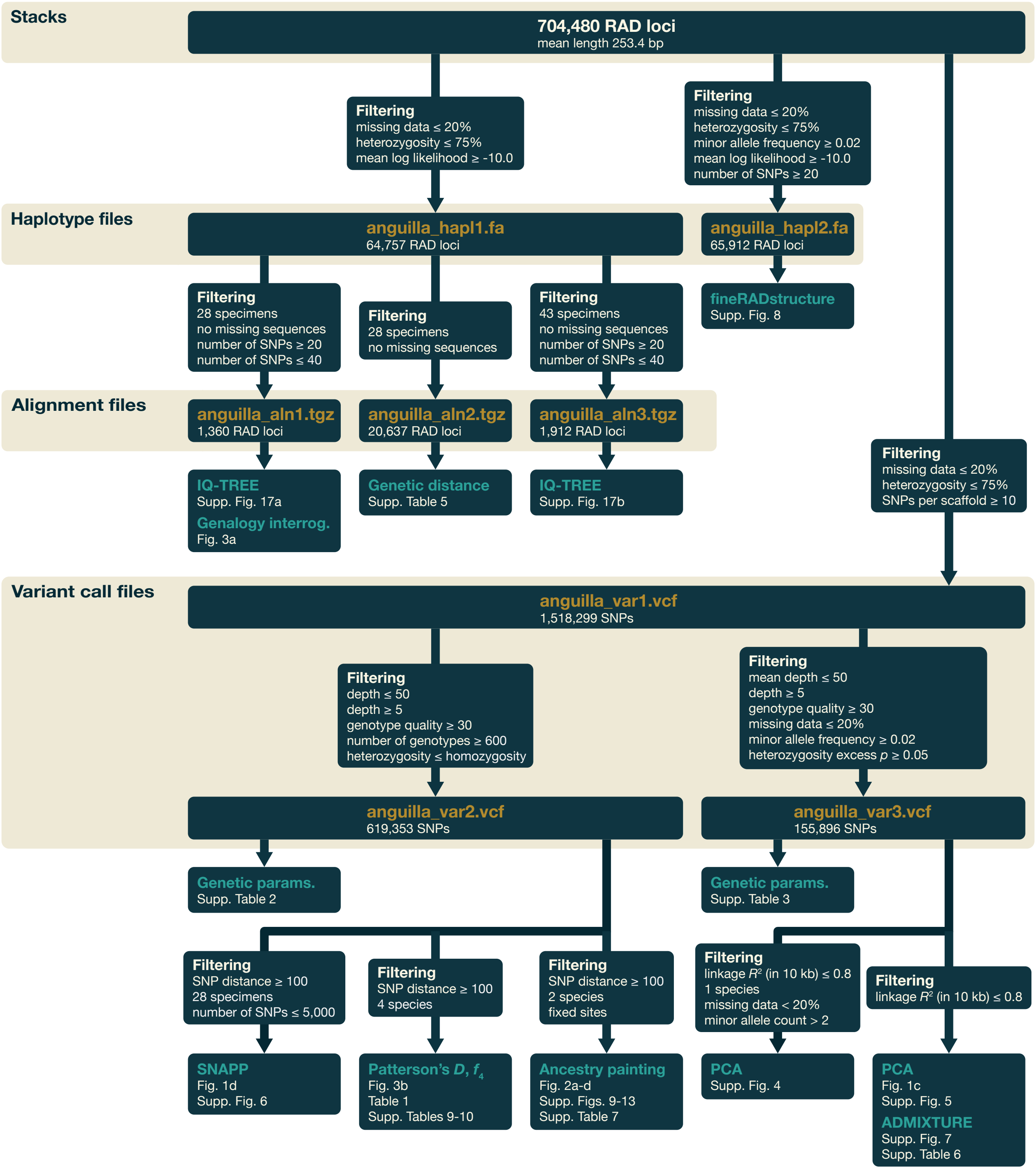

Flow chart illustrating how RAD sequencing data was filtered and used for various analyses. File names are shown in yellow and analyses are highlighted in cyan.

Supplementary Figure 2: Haplotype-genealogy graph based on mitochondrial sequences.

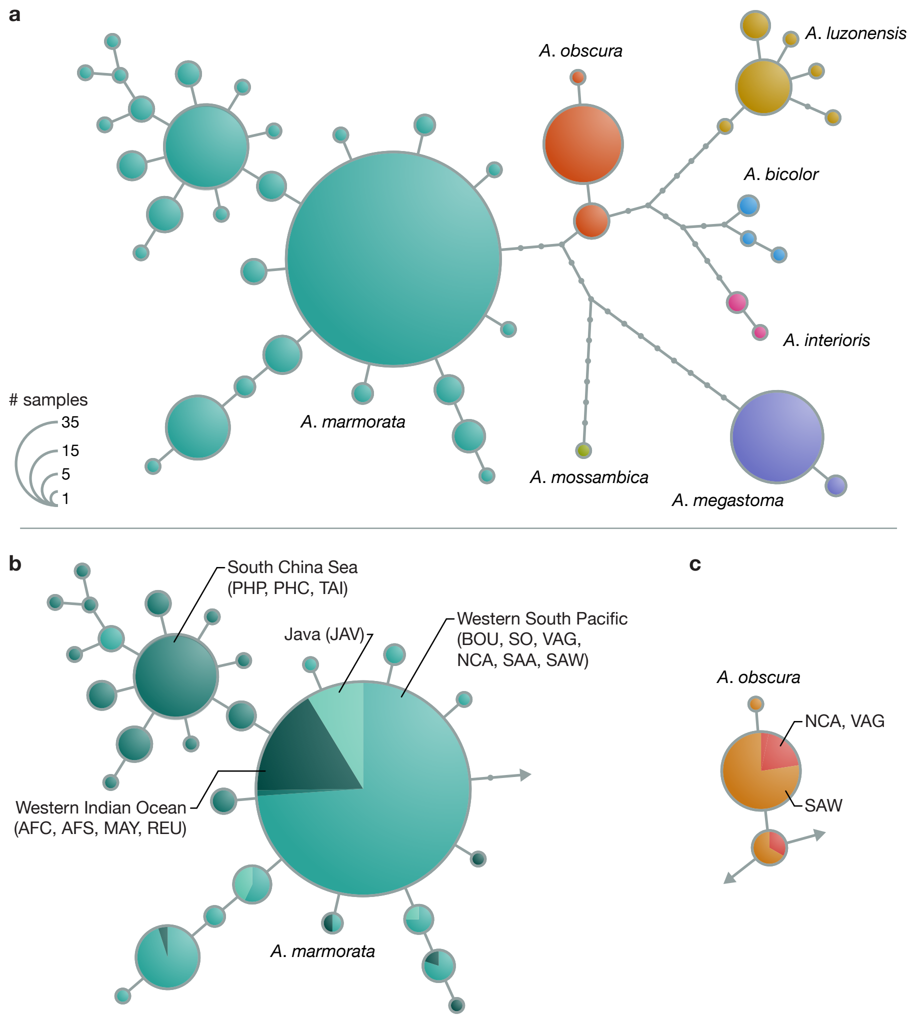

a) Graph generated with the software Fitchi v.1.1.4 [35] for two concatenated RAD loci mapping to positions 10630-10720 and 12015-12105 of the *Anguilla japonica* mitochondrial genome (NCBI accession CM002536). The genealogy of mitochondrial sequences for all 456 individuals was produced using RAxML v. 8.2.11 [36] with the GTRCAT model of sequence evolution. **b)** As a) but only showing *A. marmorata* individuals, with different shades for the four geographically distinct populations (western Indian Ocean, Java, South China Sea, and western South Pacific). **c)** As a) but only showing *A. obscura* individuals, with different shades for geographically distinct sampling sites. A single *A. obscura* individual from New Caledonia (NCA), NCA16015, is grouped with eight individuals from Vanuatu (VAG).

Supplementary Figure 3: Morphological variation among tropical eel species.

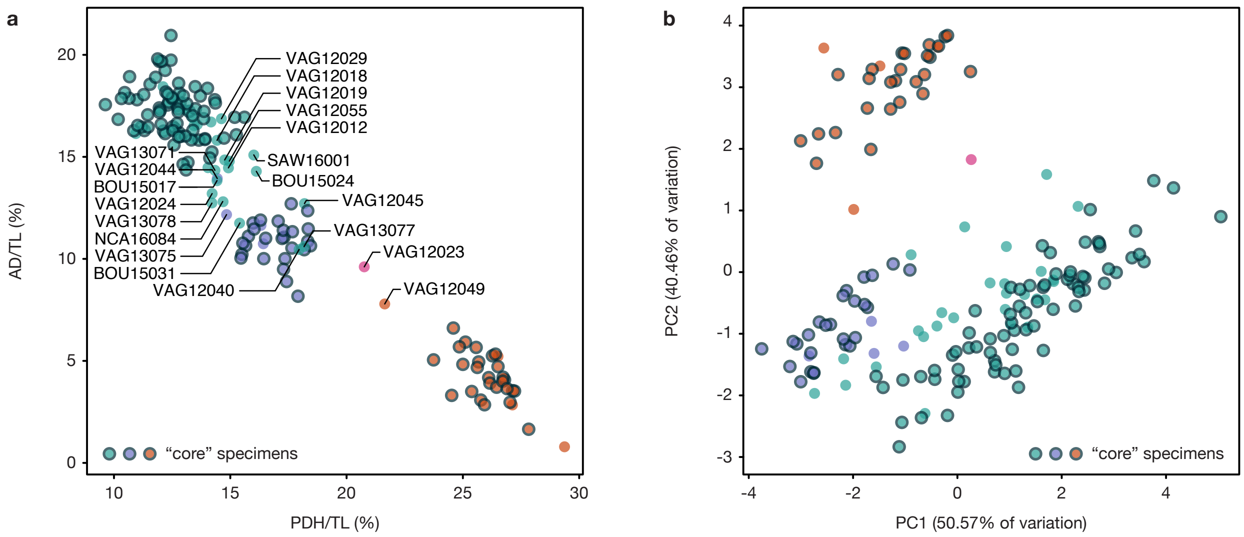

a) Following [37], the predorsal length without the head (PDH) and the distance between the dorsal fin and the anus (AD) were measured for 161 individuals available for morphological analyses (100 × *A. marmorata*, 30 × *A. megastoma*, 30 × *A. obscura*, and 1 × *A. interioris*) and standardized by terminal length (TL). Color code is identical to Supplementary Figure 2a. Individuals selected as putatively unadmixed "core" group representatives of *A. marmorata*, *A. megastoma*, and *A. obscura* are marked with dark gray outlines. Specimen IDs (see Supplementary Table 1) are given for putative hybrids and one representative of *A. interioris*. **b)** First and second principal components of morphological variation. "Core" individuals were selected according to clusters shown in this plot.

Supplementary Figure 4: Genomic variation within tropical eel species.

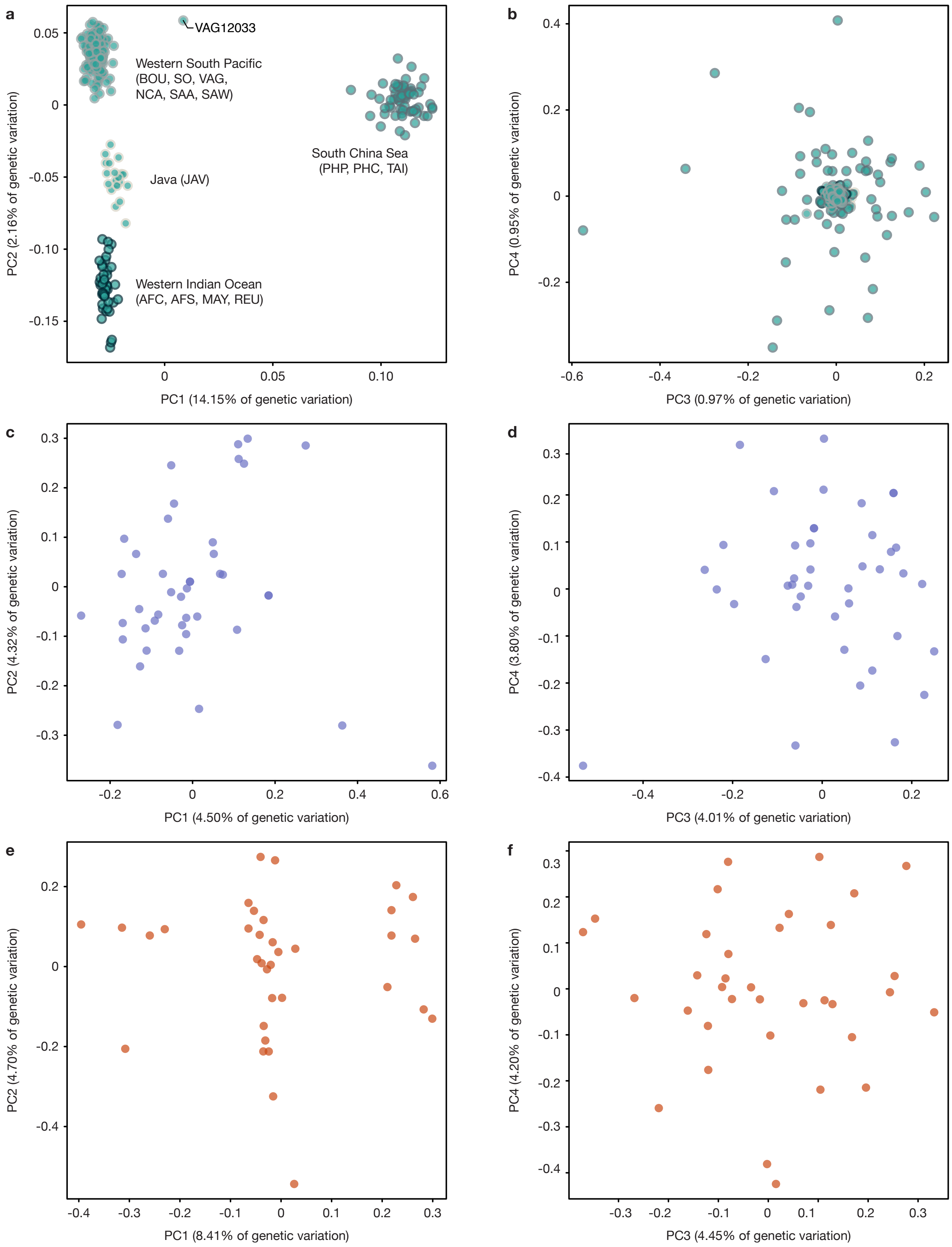

a-b) Comparison of first and second (a), and third and fourth (b), principal components of genomic variation in *A. marmorata*. Fill and stroke colors indicates geographic origin. The individual VAG12033 is characterized by a large proportion of missing data (see Supplementary Table 1), which might explain its outlier positions. **c-d)** Comparison of first and second (c), and third and fourth (d), principal components of genomic variation in *A. megastoma*. **e-f)** Comparison of first and second (e), and third and fourth (f), principal components of genomic variation in *A. obscura*. Even though *A. obscura* individuals appear to form three clusters along PC1 (e), these are not correlated to sampling site, sampling year, morphology, or proportion of missing data, and are therefore not further discussed. Putative between-species hybrids (see Supplementary Table 7) were exluded from this analysis.

Supplementary Figure 5: Genomic variation among tropical eel species.

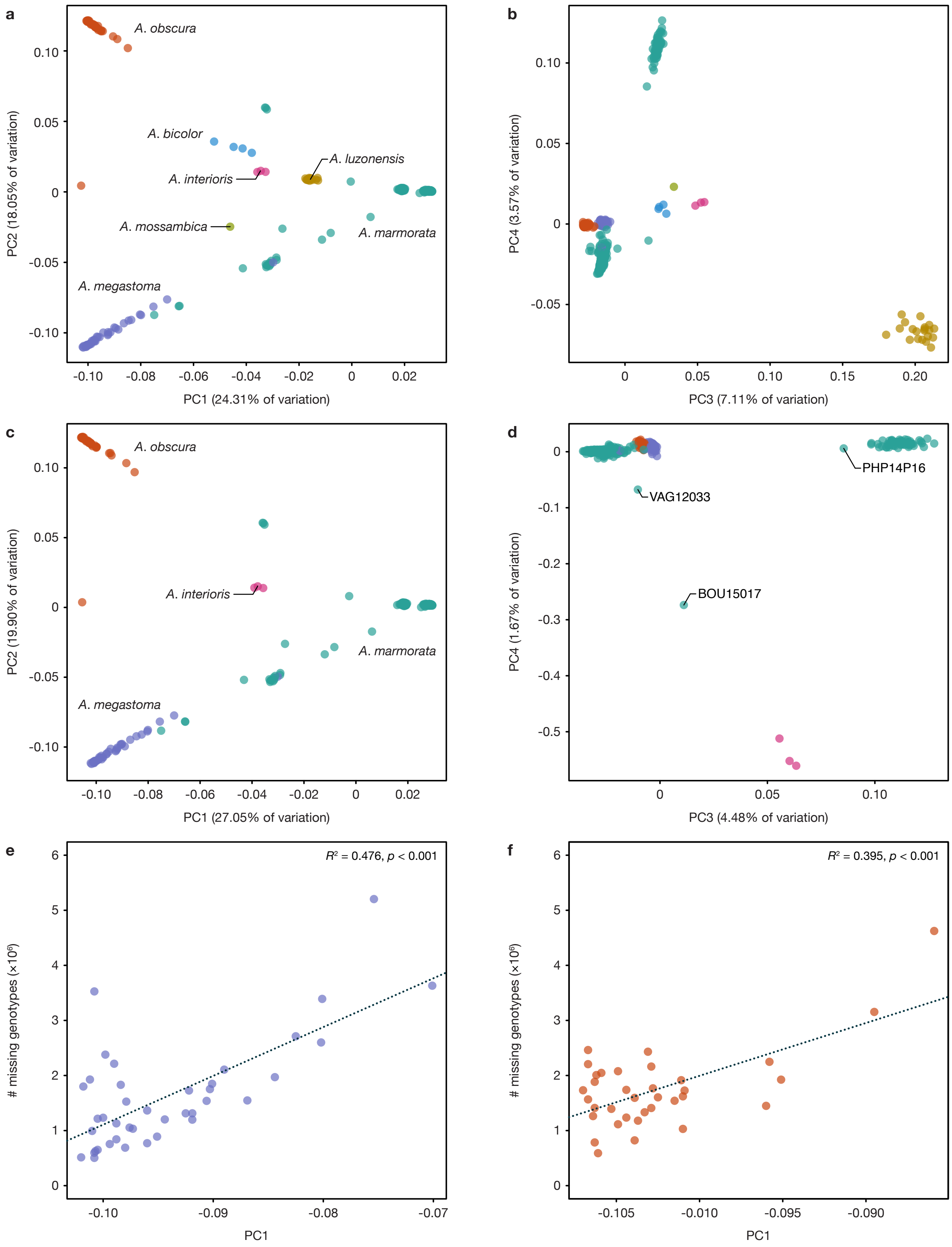

a) First and second principal components of genomic variation among the seven species *A. marmorata* (cyan), *A. luzonensis* (brown), *A. megastoma* (purple), *A. obscura* (red), *A. bicolor* (blue), *A. interioris* (magenta), and *A. mossambica* (green). **b)** Third and fourth principal components of genomic variation among the seven species. **c)** First and second principal components of genomic variation, focusing on the four species *marmorata* (cyan), *A. megastoma* (purple), *A. obscura* (red), and *A. interioris* (magenta). **d)** Third and fourth principal components of genomic variation, for the same four species as in c). The two individuals VAG12033 and PHP14P16 are characterized by a large proportion of missing data (see Supplementary Table 1), which might explain their outlier positions. **e)** First principal component of genomic variation and number of missing genotypes in *A. megastoma*. The significant correlation indicates that the positions of *A. megastoma* individuals within an elongated cluster in c) are influenced by missing data rather than by different admixture proportions (homogeneous admixture proportions among *A. megastoma* individuals are also suggested by patterns of coancestry shown in Supplementary Figure 8). **f)** As e) but for *A. obscura*.

Supplementary Figure 6: Divergence times of tropical eel species.

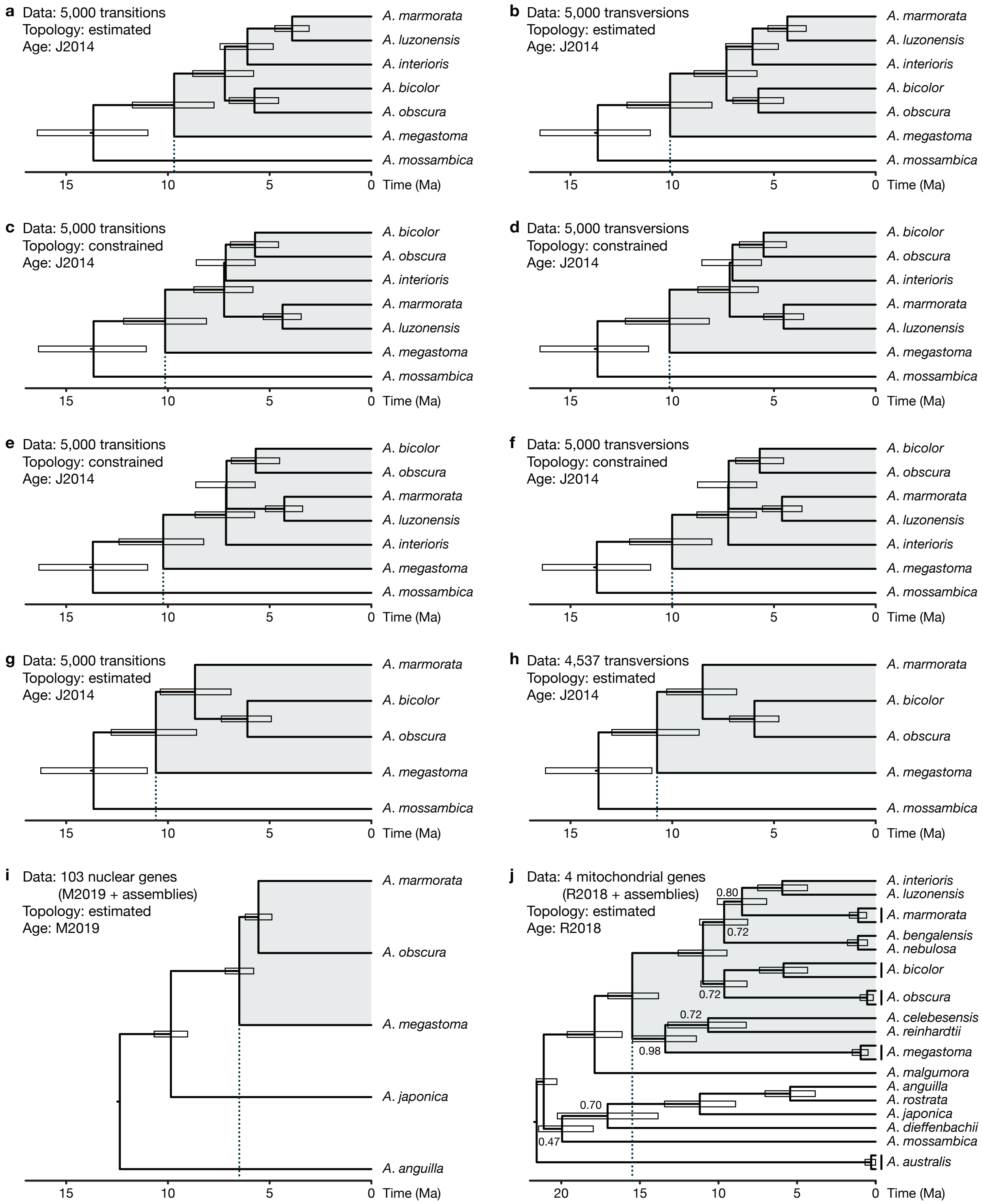

a-b) Maximum-clade-credibility (MCC) summary trees of SNAPP analyses with 5,000 transition or transversion sites, respectively, without topology constraints and a single age constraint on the root divergence according to [12] (J2014). Node bars indicate 95% highest posterior density intervals intervals. The gray area marks the group of species for which we find evidence of past and ongoing hybridization, and the dotted line indicates the crown age of this group. **c-d)** As a-b), but with a topology constraint on the position of *A. interioris* as the sister species to a clade combining *A. bicolor* and *A. obscura*; this position is supported by maximum-likelihood inference with IQ-TREE (Supplementary Figure 19). **e-f)** As a-b), but with a topology constraint on the position of *A. interioris* as the outgroup to a formed by *A. bicolor*, *A. obscura*, *A. marmorata*, and *A. luzonensis*; this position is supported by genealogy interrogation (Fig. 3a). **g-h)** As a-b), but excluding the the two species *A. luzonensis* and *A. interioris* due to their strong signals of ancient hybridization. **i)** MCC tree of a BEAST analysis with 103 nuclear genes. Sequences of these genes from *A. anguilla* and *A. japonica* were included in [18] (M2019) and were here complemented with orthologs extracted from the new genome assemblies of *A. marmorata*, *A. megastoma*, and *A. obscura*. A single age constraint on the root was used for calibration according to [18]. **j)** MCC tree of a BEAST analysis with four mitochondrial genes that were used in [19] (R2018). The dataset of [19] was here complemented with orthologs from the three new genome assemblies (*A. marmorata*, *A. megastoma*, and *A. obscura*), and a single age constraint on the root was used according to [19]. Note that the timescale in j) differs from a-i). Unless specified, all nodes received full Bayesian support.

Supplementary Figure 7: Maximum-likelihood ancestry inference.

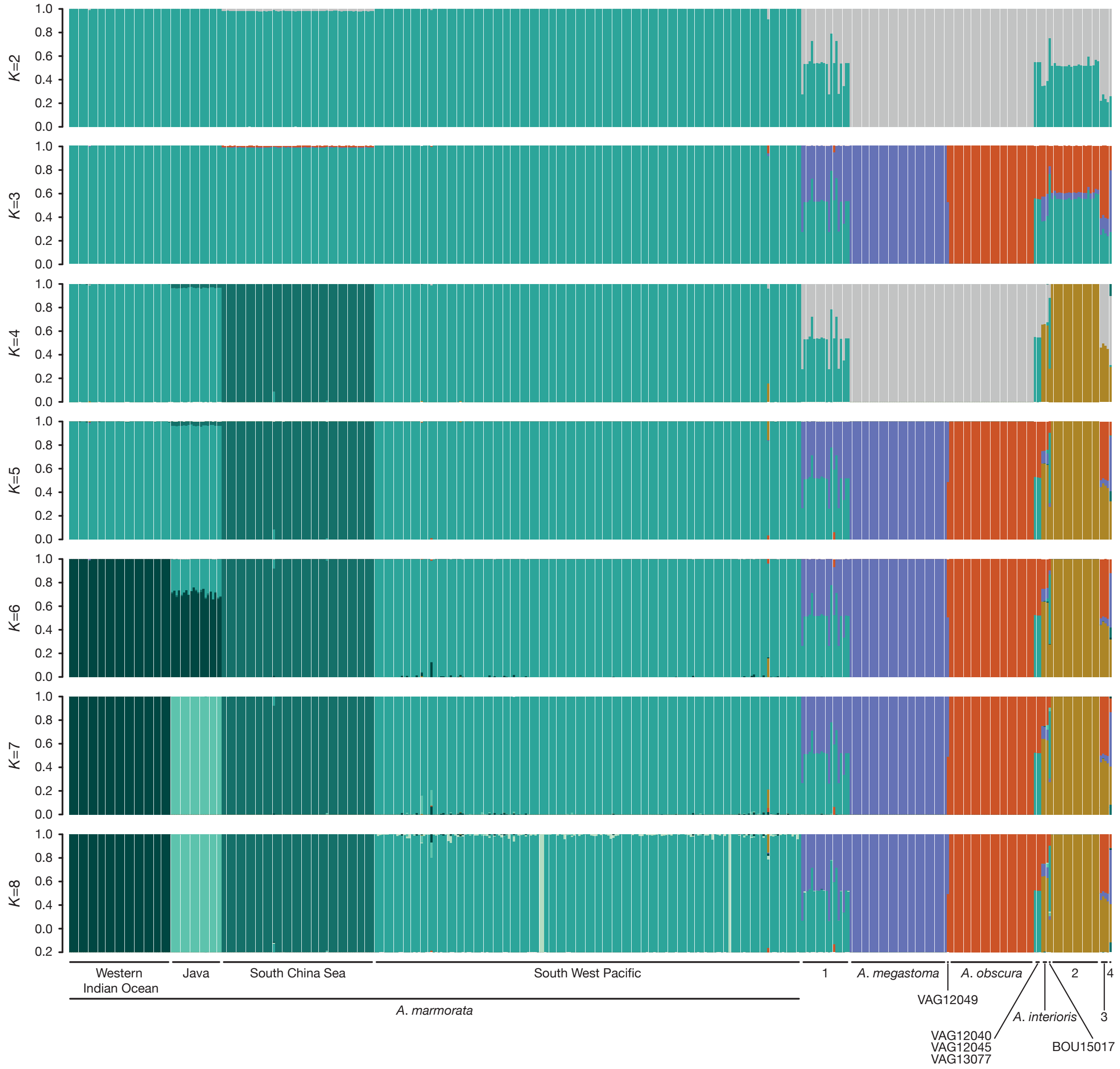

Ancestry proportions displayed in bars per individual were inferred for the models $K = 1$ to $K = 8$ based on 117,638 variable sites using the software ADMIXTURE [38]. Cross-validation (CV) errors are given in Supplementary Table 6. For *A. marmorata*, individuals are labeled according to geographic origin: Indian Ocean (sampling locations AFC, AFS, MAY, REU), Java, South China Sea (PHP, PHC, TAI), and Pacific (BOU, NCA, SAA, SAW, SO, VAG). Label 1 marks 20 individuals that appear admixed between *A. marmorata* and *A. megastoma*: BOU15031, SAA16011, SAA16012, SAA16013, SAA16024, SAA16027, SAW17B27, SAW17B49, VAG12012, VAG12018, VAG12019, VAG12024, VAG12029, VAG12037, VAG12044, VAG12053, VAG12055, VAG13071, VAG13078, and VAG13087. Labels 2-4 indicate species *A. luzonensis* (2), *A. bicolor* (3), and *A. mossambica* (4).

Supplementary Figure 8: Individual coancestry based on haplotype similarity.

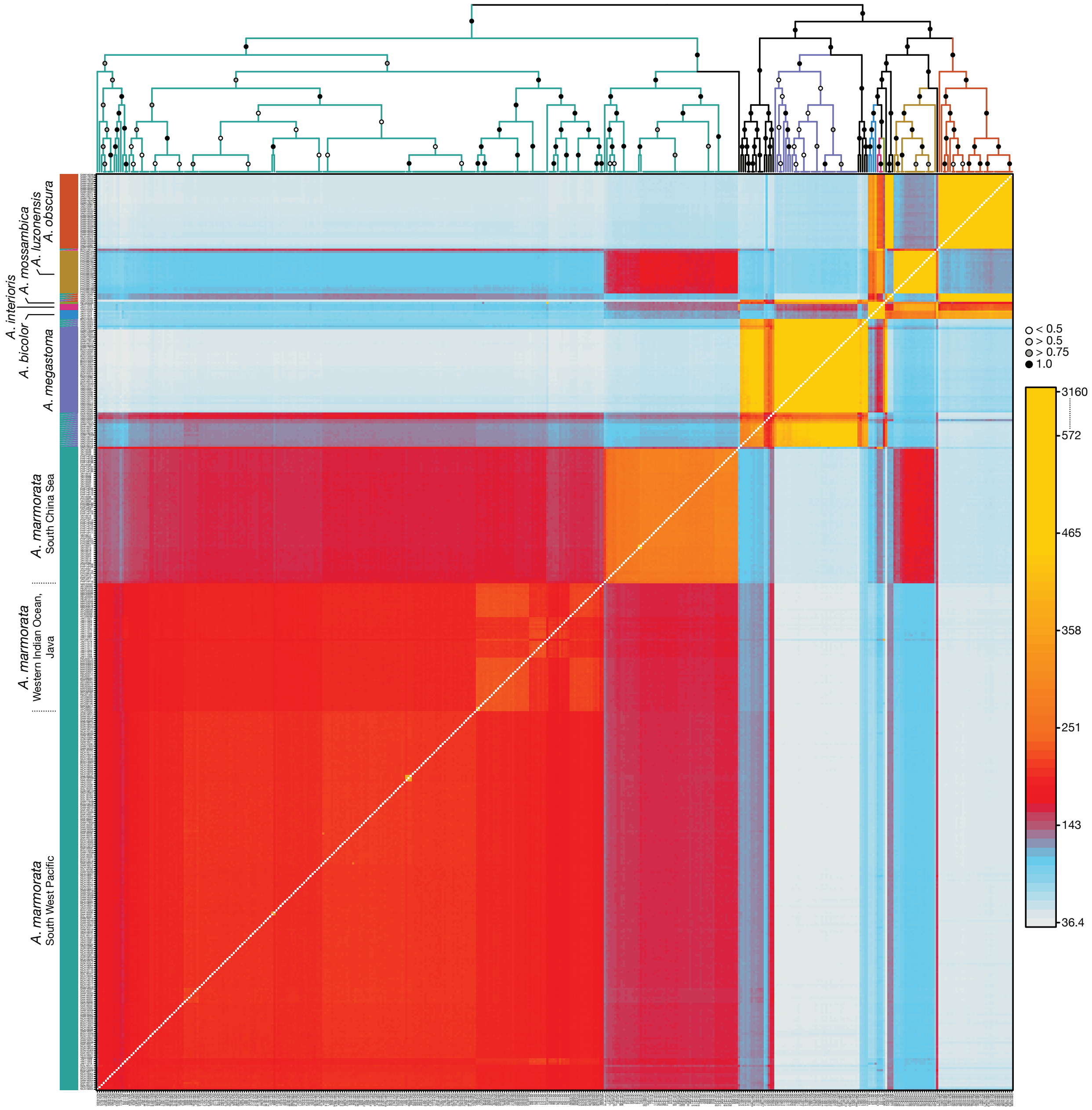

Coancestry was investigated based on RAD loci with fineRADstructure [39]. Heatmap colors indicate numbers of RAD loci with estimated shared coancestry. Individuals are listed on both axes in the same order, clustered according to the tree shown on top of the heatmap [40]. Note that even though *A. luzonensis* appears to have more shared coancestry with the South China Sea population of *A. marmorata* than with other populations, introgression between *A. luzonensis* and the South China Sea population of *A. marmorata* is not supported by D and f_4 statistics (Supplementary Table 10).

Supplementary Figure 9: Ancestry painting for *A. marmorata* and *A. megastoma.*

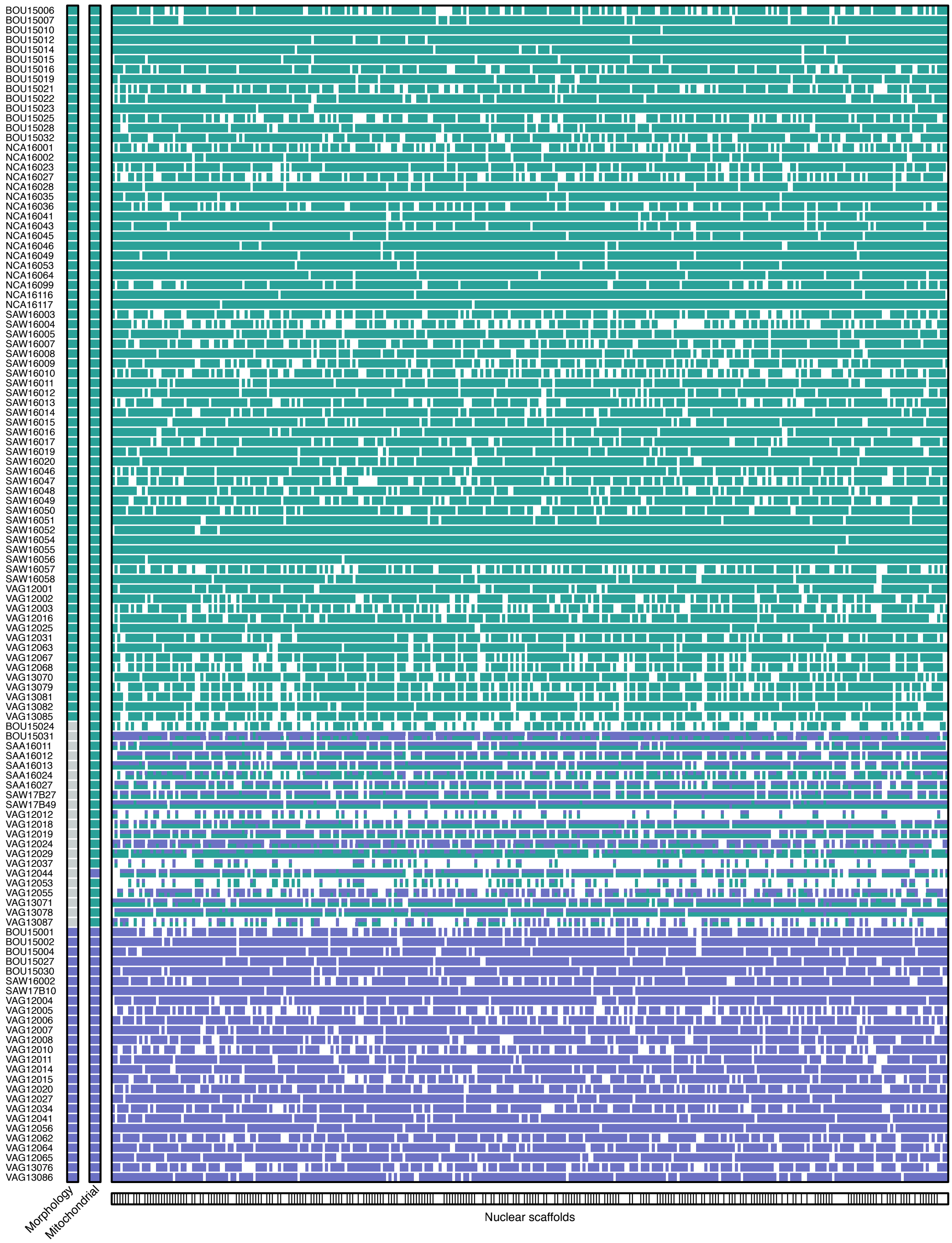

Ancestry painting for 73 “core” *A. marmorata* individuals, 26 “core” *A. megastoma* individuals, and 20 recent hybrids between the two species. In addition, one *A. marmorata* individual (BOU15024) was included because it was initially assumed to be a hybrid based on morphological measurements (Supplementary Figure 3a); this assumption is not supported by the ancestry painting shown here. Horizontal bars indicate the genotypes at each of 302 sites fixed between the two parental species. White color indicates missing data. Heterozygous genotypes are shown with the top half in each bar matching the second parental species and vice versa. Light gray cells in the morphology column indicate individuals not classified into any of the “core” groups. The species’ color code is identical to Supplementary Figure 2a.

Supplementary Figure 10: Ancestry painting of long scaffolds for backcrossed hybrids between *A. marmorata* and *A. megastoma*.

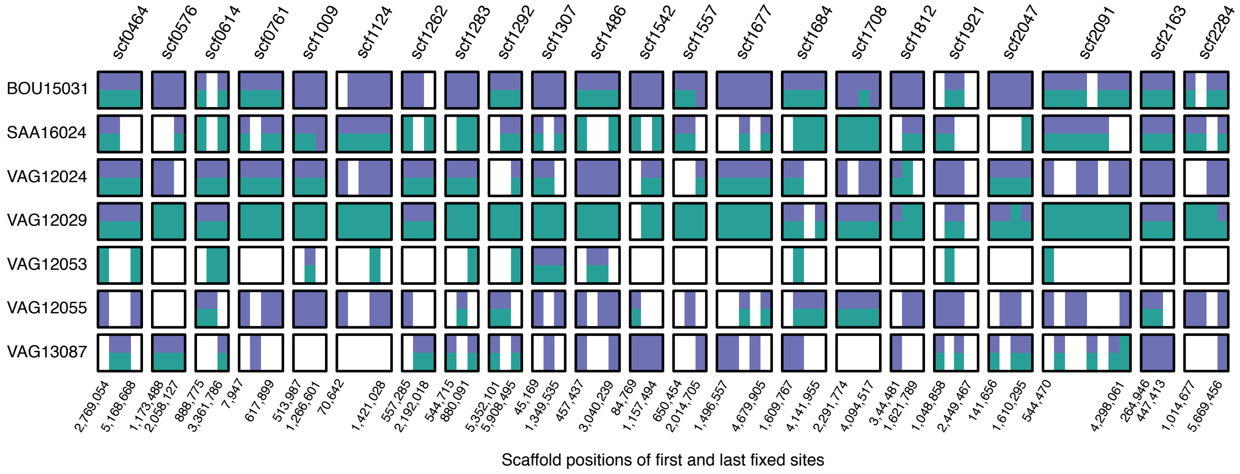

Information shown here is a part of that presented in Supplementary Figure 9, focusing only on backcrossed hybrids and their genotypes on scaffolds with at least three fixed sites. Scaffold IDs are given on top and the positions of the first and last of the sites fixed on this scaffold are given below the ancestry painting. A single change from heterozygous to homozygous states or vice versa occurs six times among the seven individuals and two such changes on the same scaffold occur twice. With a mean distance of 1,708,024 bp and a maximum distance of 4,654,779 bp between the first and the last of the sites assessed on these 22 scaffolds, recombination breakpoints therefore appeared to be rare, in agreement with the interpretation of these individuals as backcrossed second-generation hybrids.

Supplementary Figure 11: Ancestry painting for *A. marmorata* and *A. obscura*.

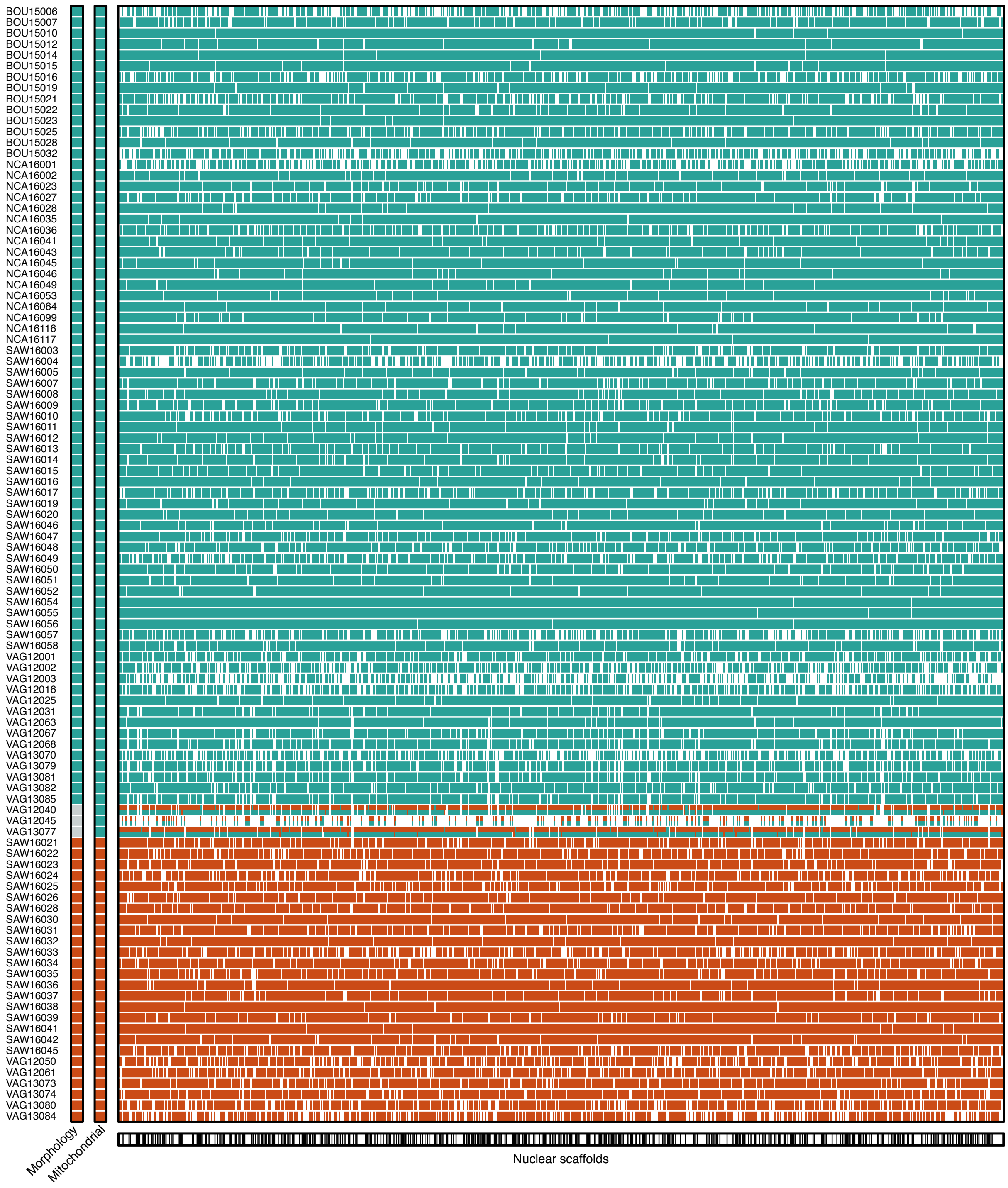

Ancestry painting as in Supplementary Figure 9, but for 73 "core" *A. marmorata* individuals, 26 "core" *A. obscura* individuals, and 3 recent hybrids between the two species. Horizontal bars indicate the genotypes at each of 742 sites fixed between the two parental species.

Supplementary Figure 12: Ancestry painting for *A. megastoma* and *A. obscura*.

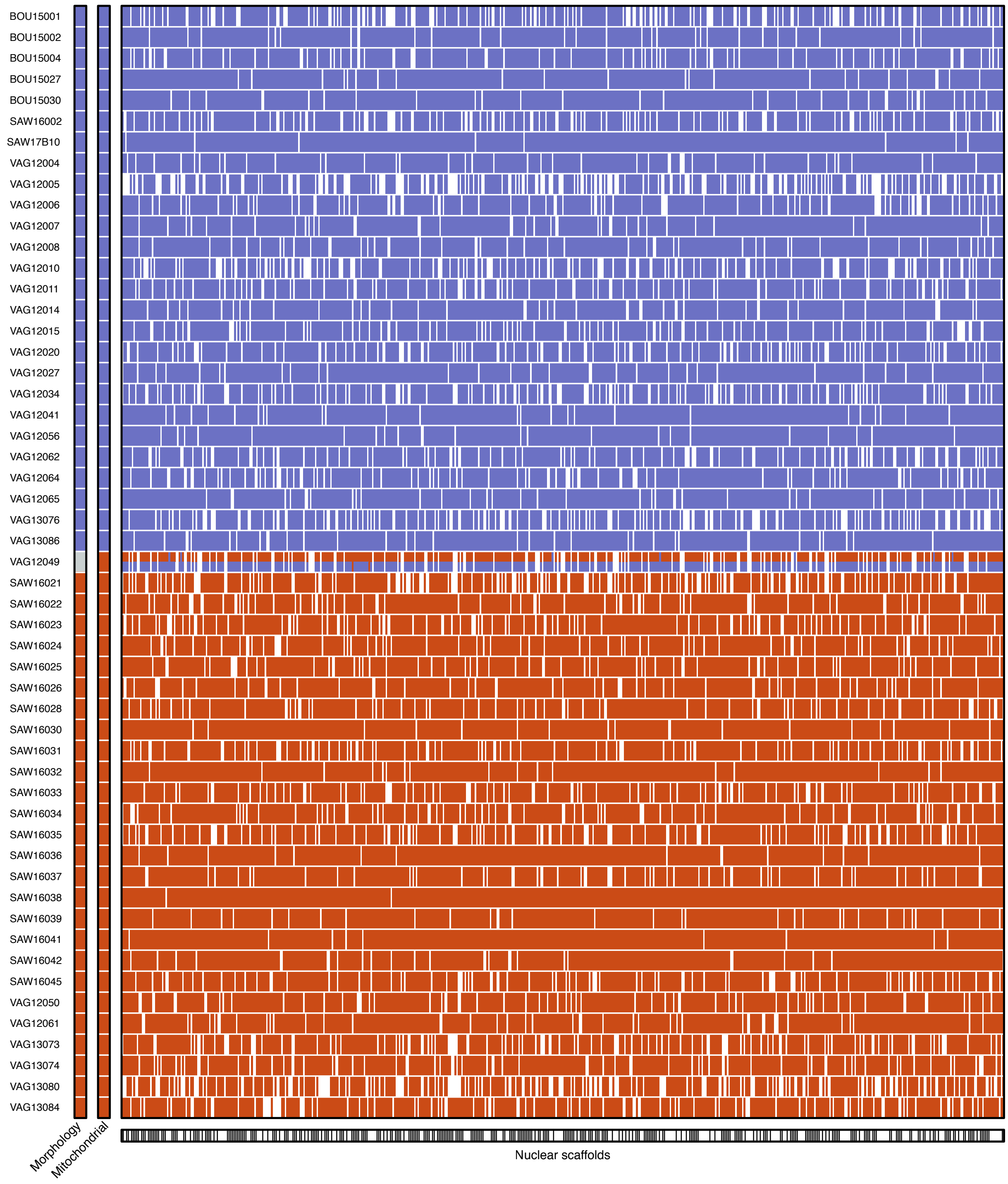

Ancestry painting as in Supplementary Figure 9, but for 26 "core" *A. megastoma* individuals, 26 "core" *A. obscura* individuals, and 1 recent hybrid between the two species. Horizontal bars indicate the genotypes at each of 525 sites fixed between the two parental species.

Supplementary Figure 13: Ancestry painting for *A. marmorata* and *A. interioris*.

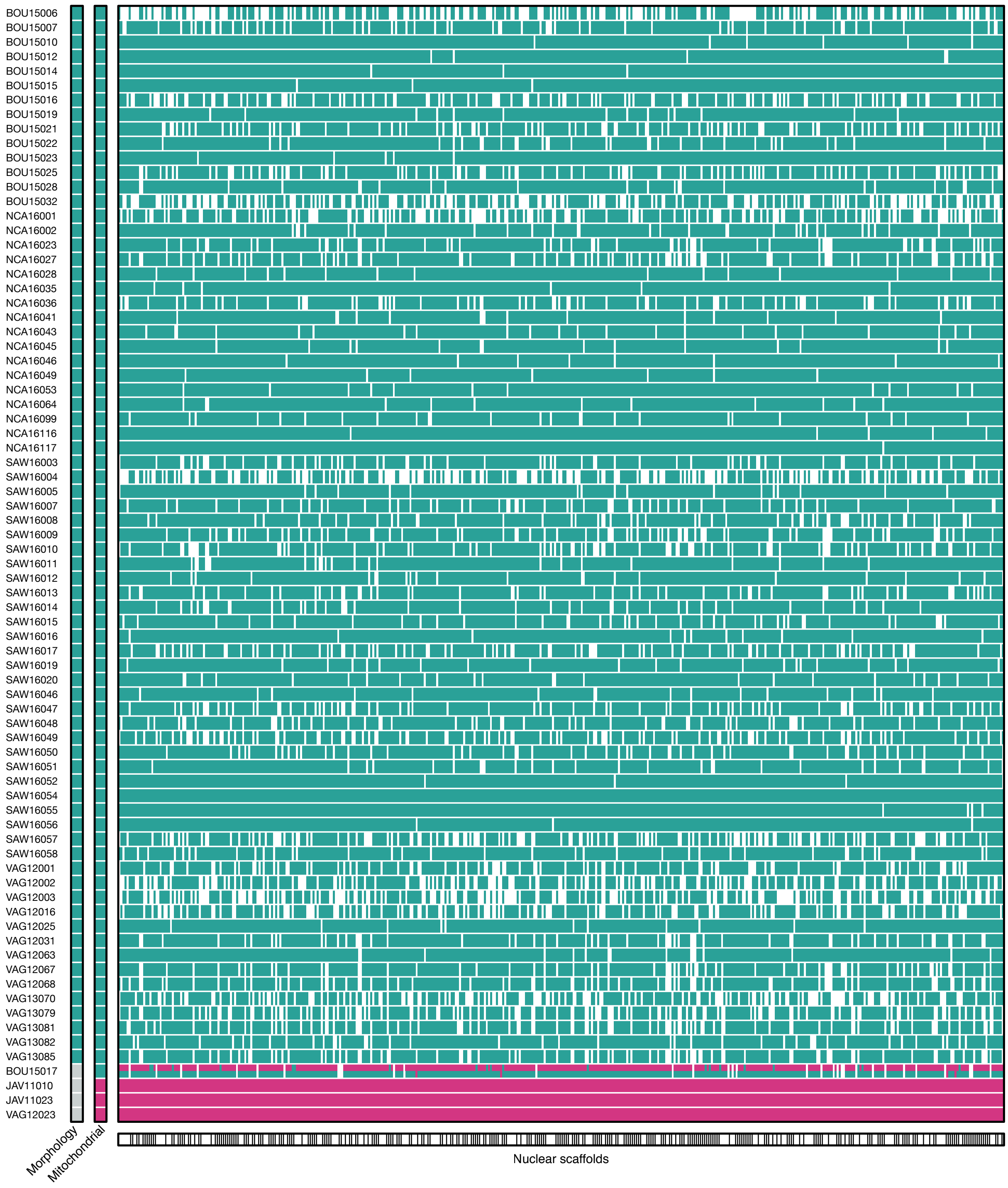

Ancestry painting as in Supplementary Figure 9, but for 73 “core” *A. marmorata* individuals, 3 *A. interioris* individuals, and 1 recent hybrid between the two species. Horizontal bars indicate the genotypes at each of 429 sites fixed between the two parental species.

Supplementary Figure 14: Hybrid frequencies per sampling location.

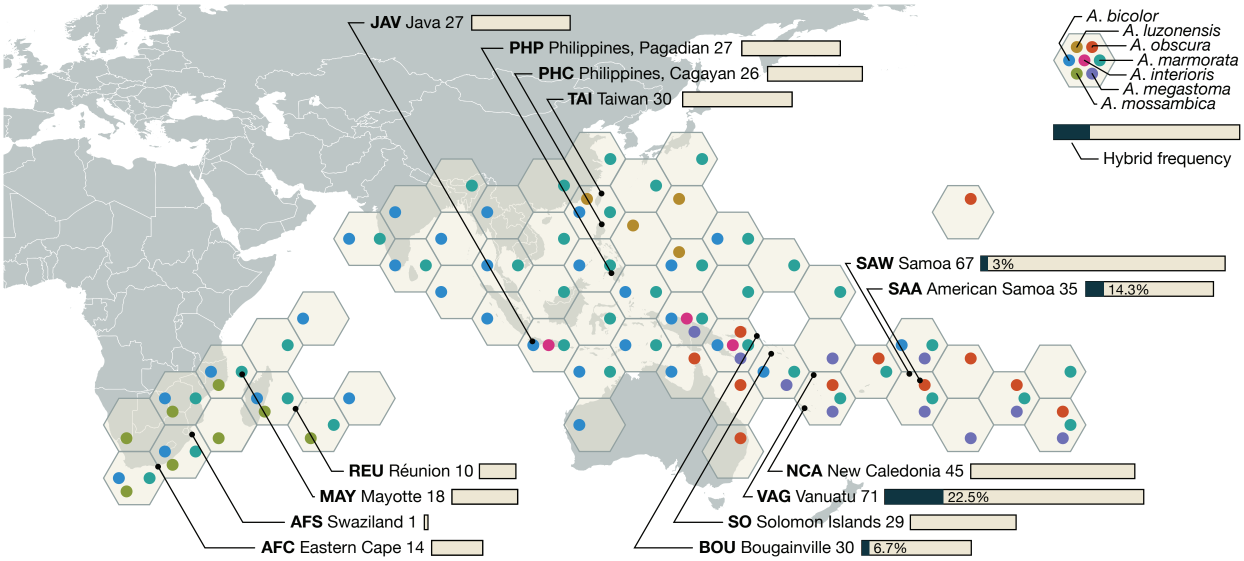

Horizontal bars indicate the numbers of individuals collected at 14 sampling locations, counting only those with sufficient sequence quality that were used in genomic analyses. Hybrid frequencies are shown as black proportions of these bars.

Supplementary Figure 15: Triangle plot for unadmixed and hybrid individuals.

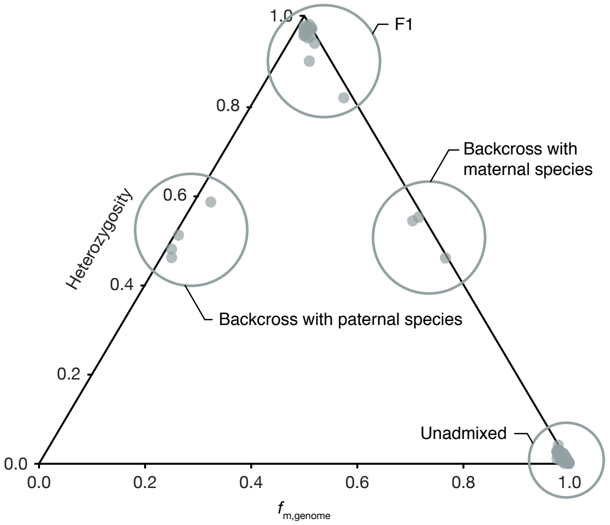

The triangle plot illustrates the proportions of nuclear genomes derived from the maternal species and heterozygosity according to Pulido-Santacruz et al.[41]. Both the proportions of the nuclear genomes derived from the maternal species, $f_{\mathrm{m,genome}}$, and the heterozygosities were calculated from genotypes at fixed sites as described in the main text. Our interpretations of individuals as unadmixed, F1, or backcross with the maternal or paternal species are indicated.

Supplementary Figure 16: Morphological variation between species pairs and their hybrids.

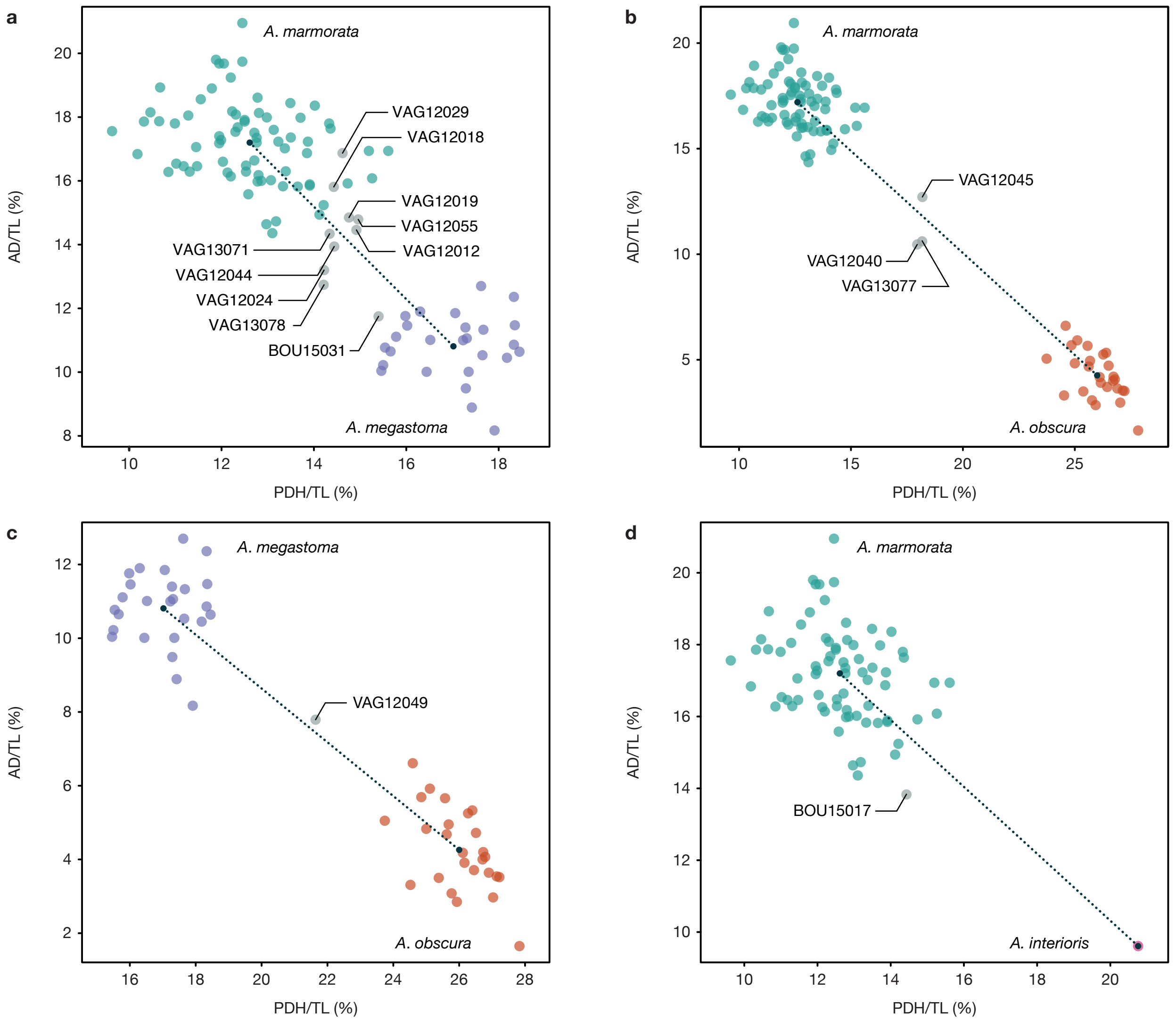

Morphological measurements followed Watanabe et al.[37]. Individuals identified as hybrids in Fig. 2 are marked with specimen IDs, excluding hybrids from Samoa (SAW) and American Samoa (SAA) and one hybrid from Vanuatu (VAG; VAG13087) for which the displayed measurements were not available (Supplementary Table 1). Mean phenotypes per species are marked with black dots that are connected by a dashed line. **a)** Morphological variation between *A. marmorata* and *A. megastoma* and their hybrids. **b-d)** As a) but for the species pairs *A. marmorata* and *A. obscura* (b), *A. megastoma* and *A. obscura* (c), and *A. marmorata* and *A. interioris* (d). The species' color code is identical to Supplementary Figure 2a. AD: distance between the dorsal fin and the anus; PDH: predorsal length without head; TL: total length.

Supplementary Figure 17: Morphology of F1 and backcrossed hybrids between *A. marmorata* and *A. megastoma*.

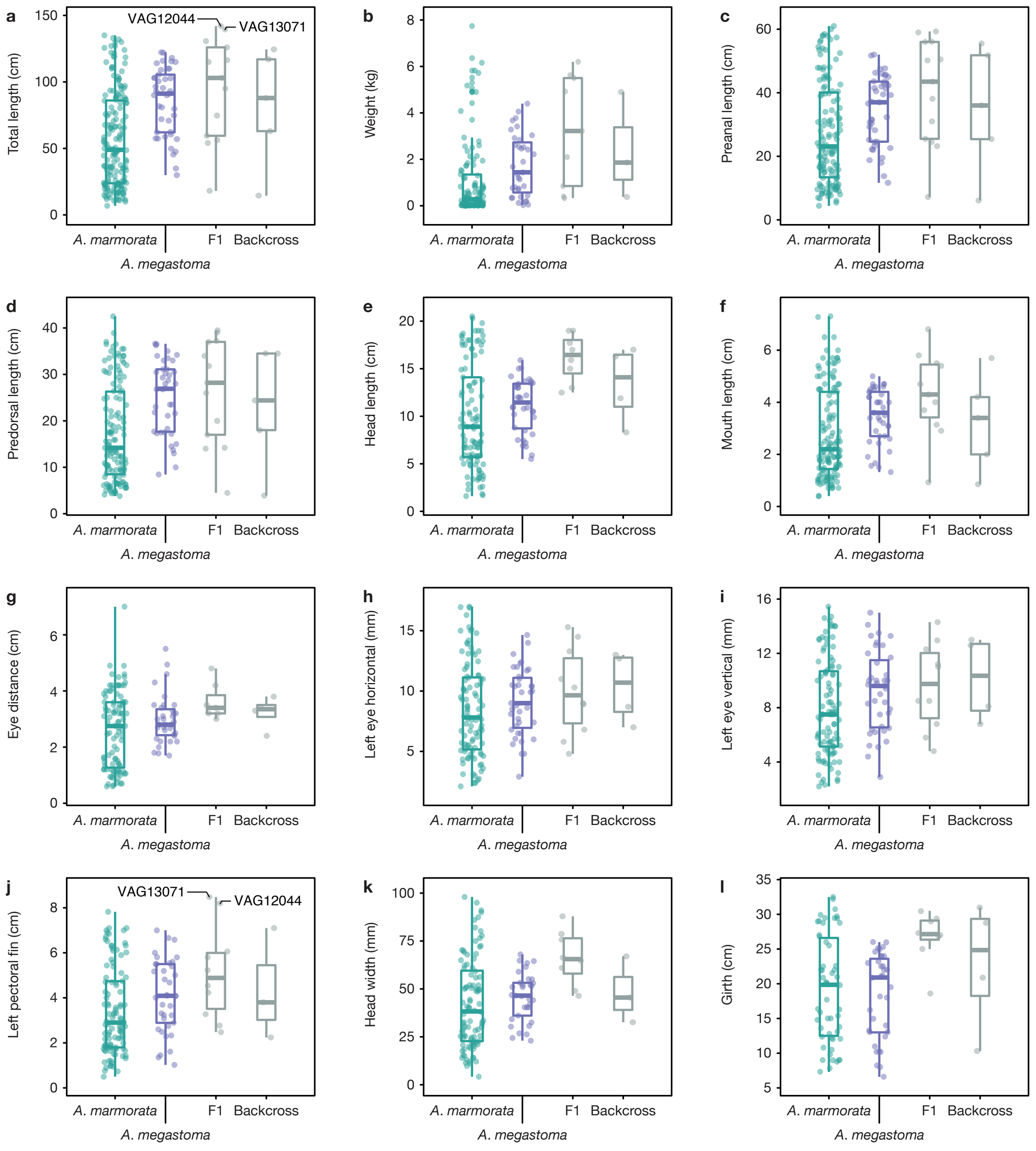

Morphological measurements followed Watanabe et al.[37]. **a-l)** Boxplots show median values and the interquartile range, and the whiskers extend to either the most extreme values or have a length corresponding to 1.5× the interquartile range. Specimen IDs are given for hybrids with transgressive phenotypes. $n = 173$ (*A. marmorata*), 42 (*A. megastoma*), 13 (F1), and 5 (backcross) individuals.

Supplementary Figure 17 (continued): Morphology of F1 and backcrossed hybrids between *A. marmorata* and *A. megastoma*.

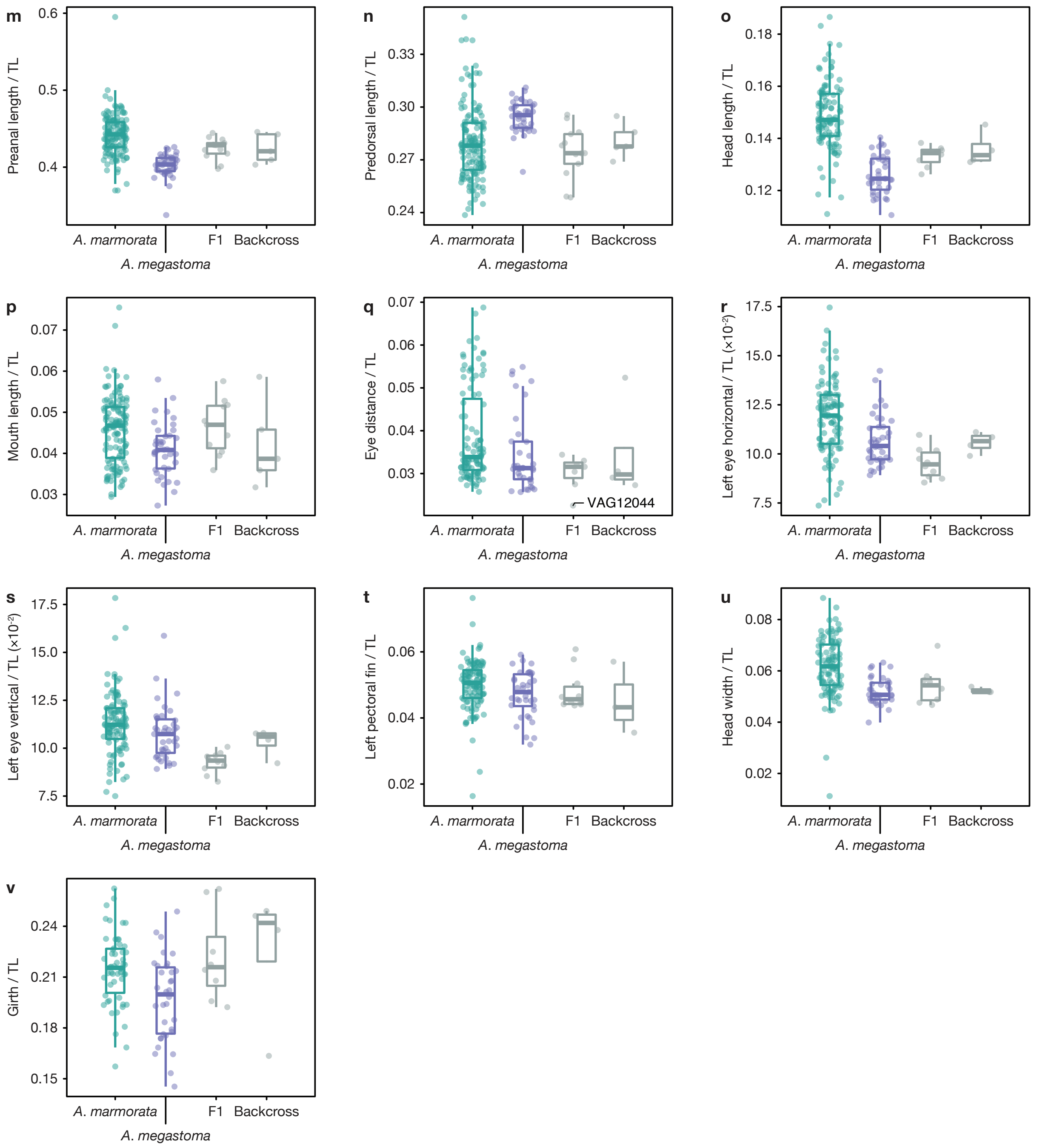

m-v) As c-l), but showing measurements standardized by terminal length (TL).

Supplementary Figure 18: Morphology of F1 hybrids between *A. marmorata* and *A. obscura*.

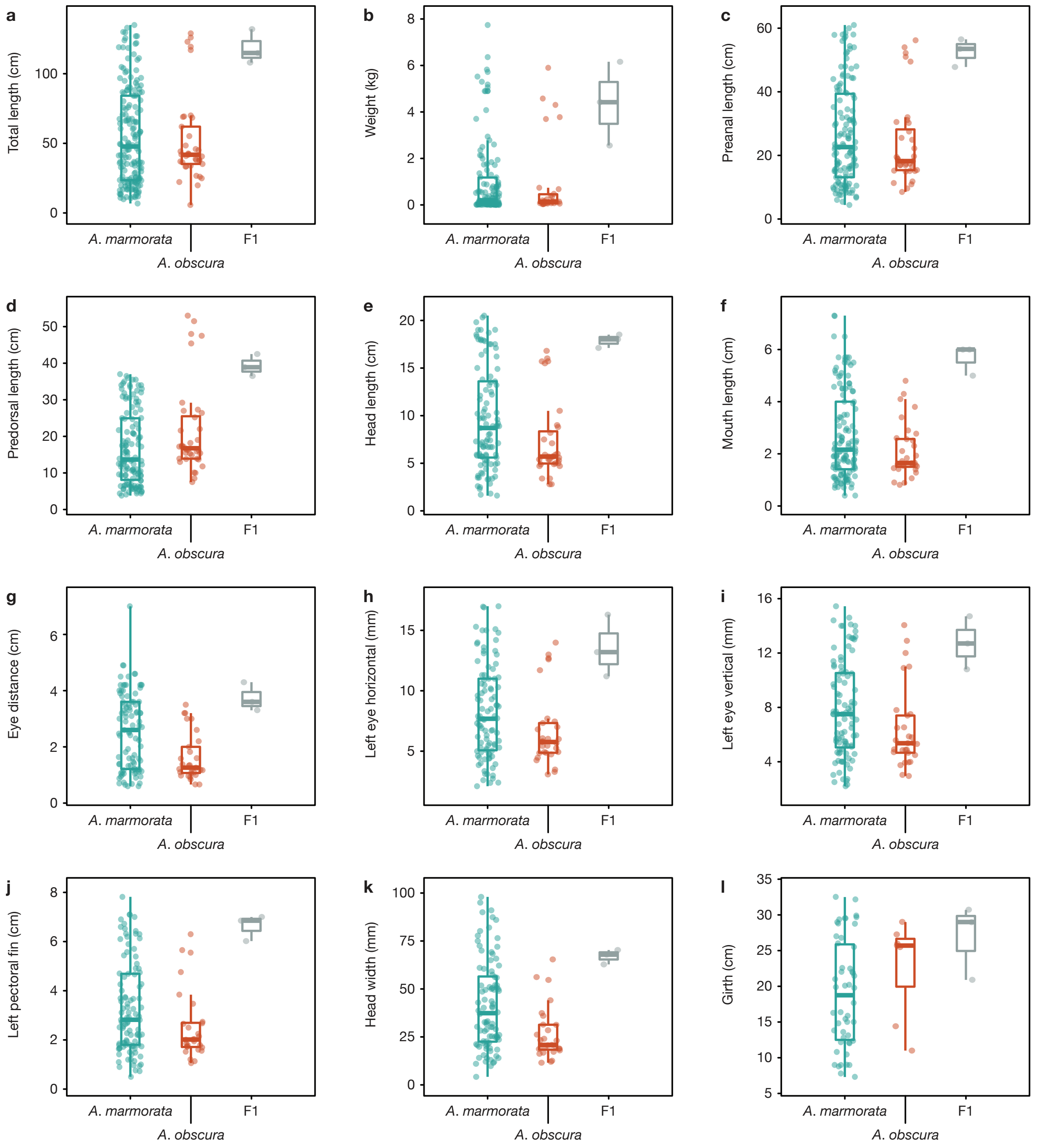

Morphological measurements followed Watanabe et al.[37]. **a-l)** Boxplots show median values and the interquartile range, and the whiskers extend to either the most extreme values or have a length corresponding to 1.5× the interquartile range. $n = 168$ (*A. marmorata*), 37 (*A. megastoma*), and 3 (F1) individuals.

Supplementary Figure 18 (continued): Morphology of F1 hybrids between *A. marmorata* and *A. obscura*.

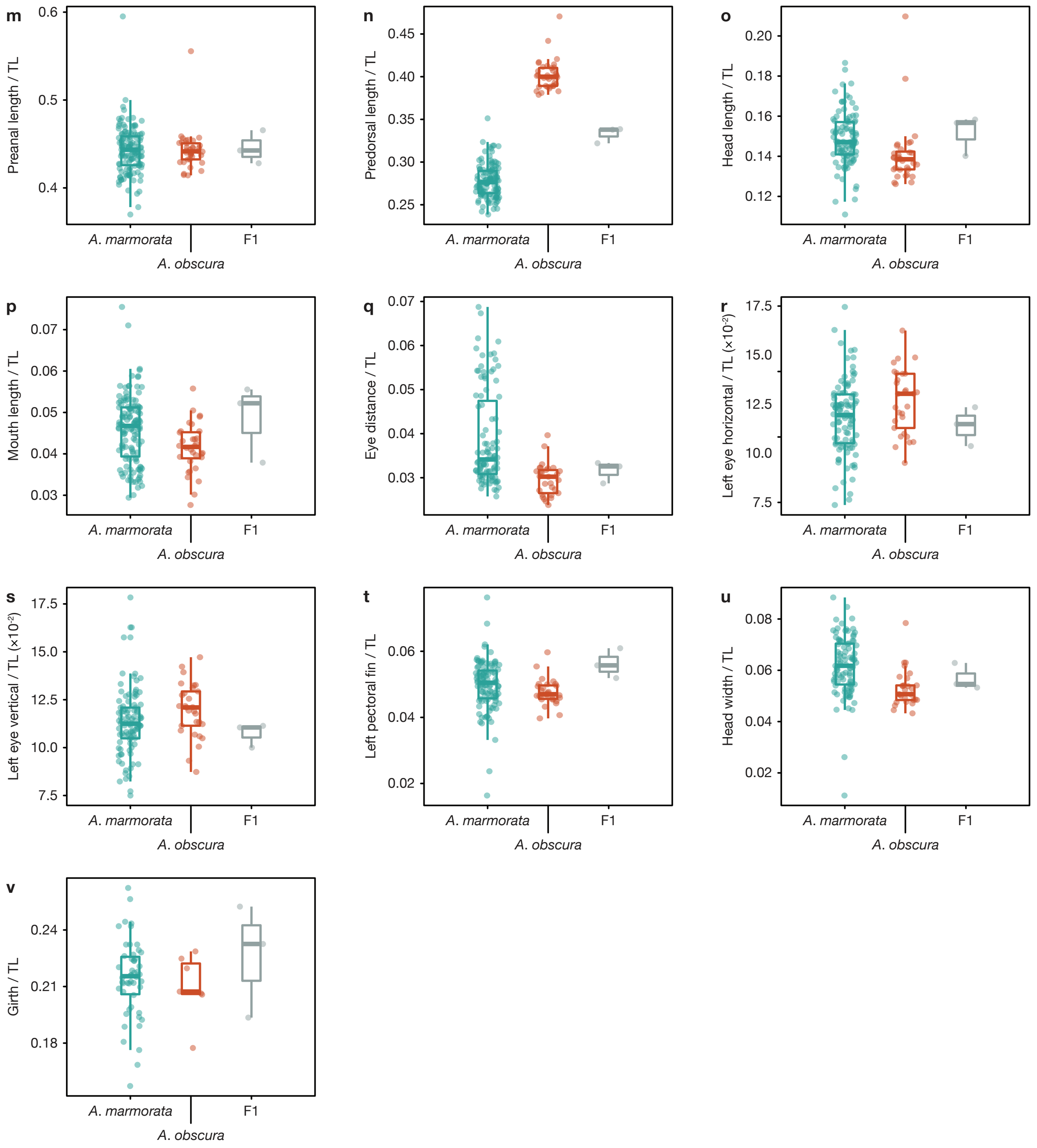

m-v) As c-l), but showing measurements standardized by terminal length (TL).

Supplementary Figure 19: Maximum-likelihood phylogenetic inference.

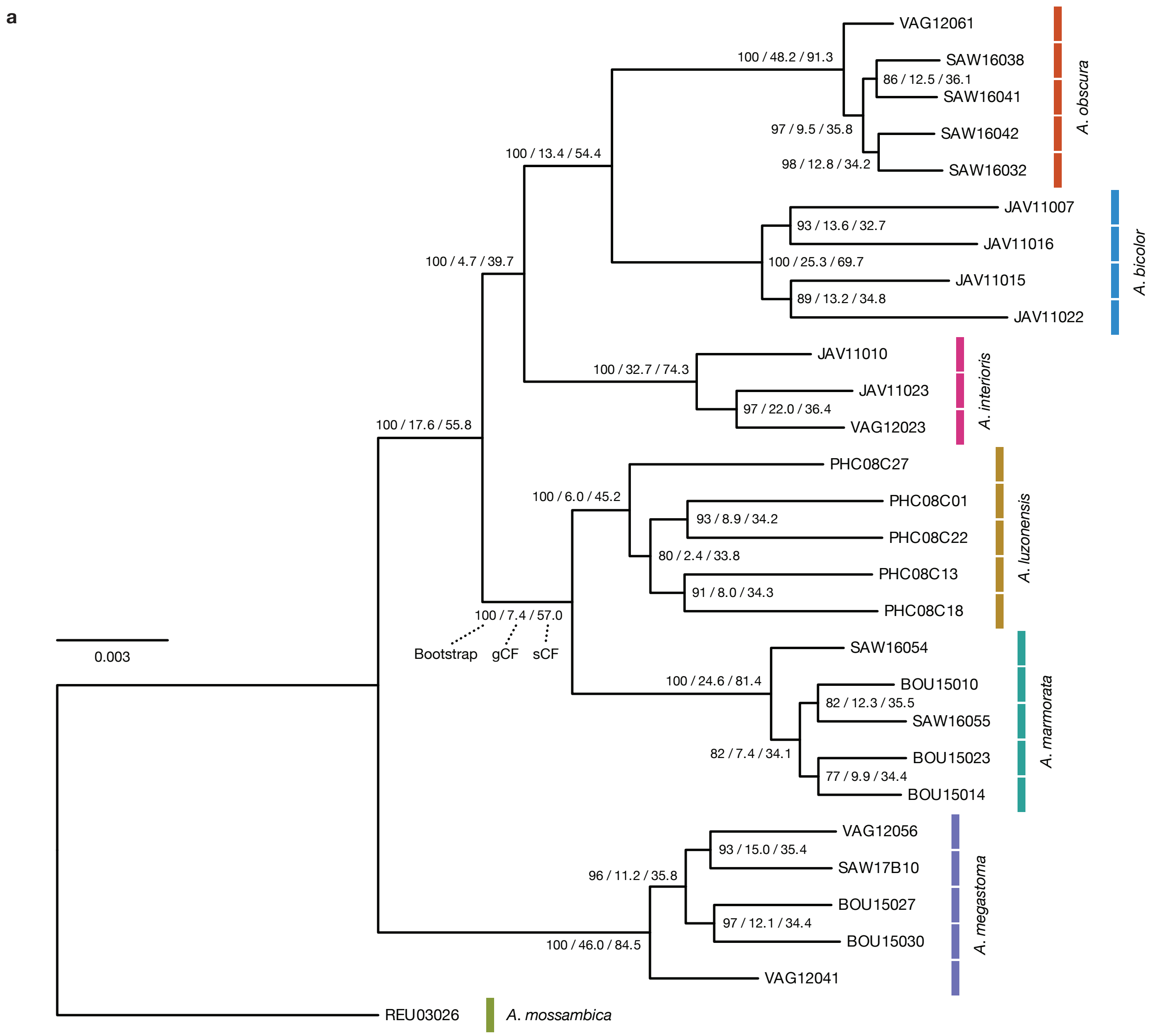

a) Phylogeny reconstructed with IQ-TREE [42] from 1,360 concatenated RAD loci without missing sequences and 20-40 variable sites per locus. Node labels indicate bootstrap support as well as per-locus (gCF) and per-site (sCF) concordance factors [43]. For *A. marmorata*, *A. megastoma*, *A. luzonensis*, and *A. obscura*, only the five individuals with the lowest proportions of missing data were used.

Supplementary Figure 19 (continued): Maximum-likelihood phylogenetic inference.

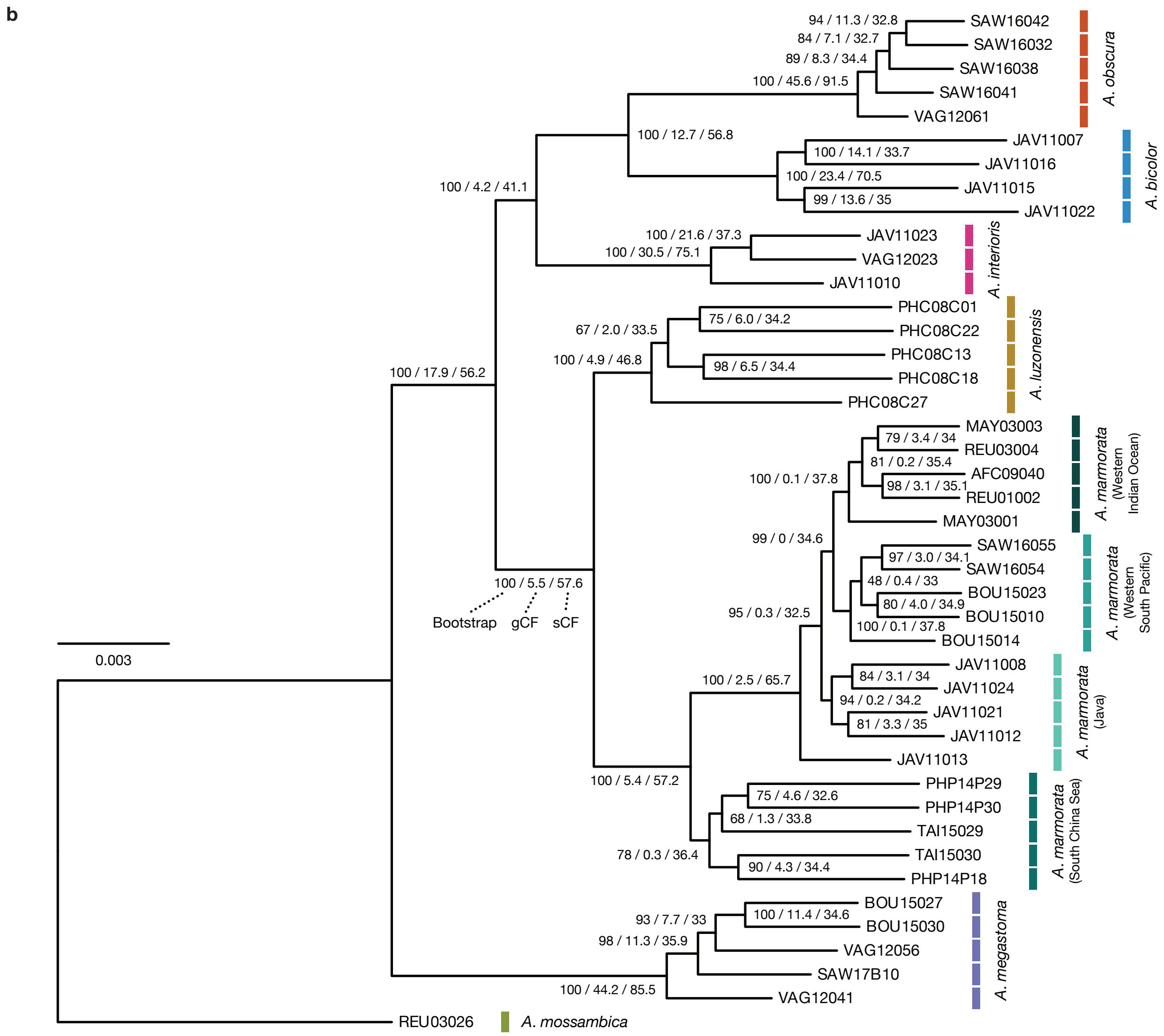

b) As a), but including five individuals from each of the four populations of *A. marmorata*. The phylogeny is reconstructed from 1,912 concatenated RAD loci without missing sequences and 20-40 variable sites per locus.

Supplementary Figure 20: Estimates of effective population size based on WGS data.

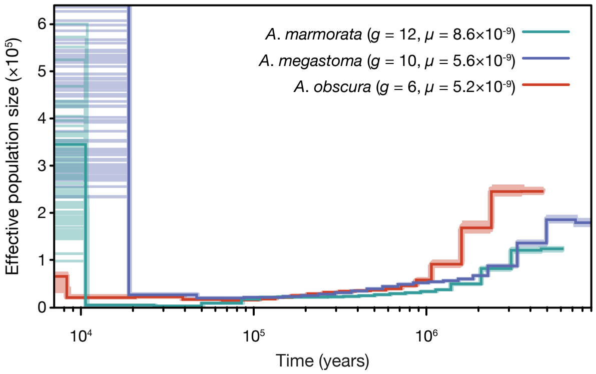

Changes in effective population size (N_e; vertical axis) over time (the last 1 myr; horizontal axis) estimated using the pairwise sequential Markovian coalescent (PSMC). The PSMC was applied to WGS data of one individual for each of the three species *A. marmorata*, *A. megastoma*, and *A. obscura*. Estimates were based on assumed generation times (g) between 6 and 12 years and mutation rates (μ) between 5.2 and 8.6 $\times$ 10^{-9} mutations/site/generation. Semi-transparent colored lines correspond to 100 bootstrap replicates. For visualization purposes, the range of contemporary N_e values is truncated for *A. megastoma*; the maximum bootstrap value is $N_e = 2.0 \times 10^6$. Note that the apparent bottleneck pattern seen in all three species could be an artifact as it is expected even without actual population-size decline when parts of the genome are affected by introgression [44].

Supplementary Figure 21: Genomic rearrangements supported by WGS data.

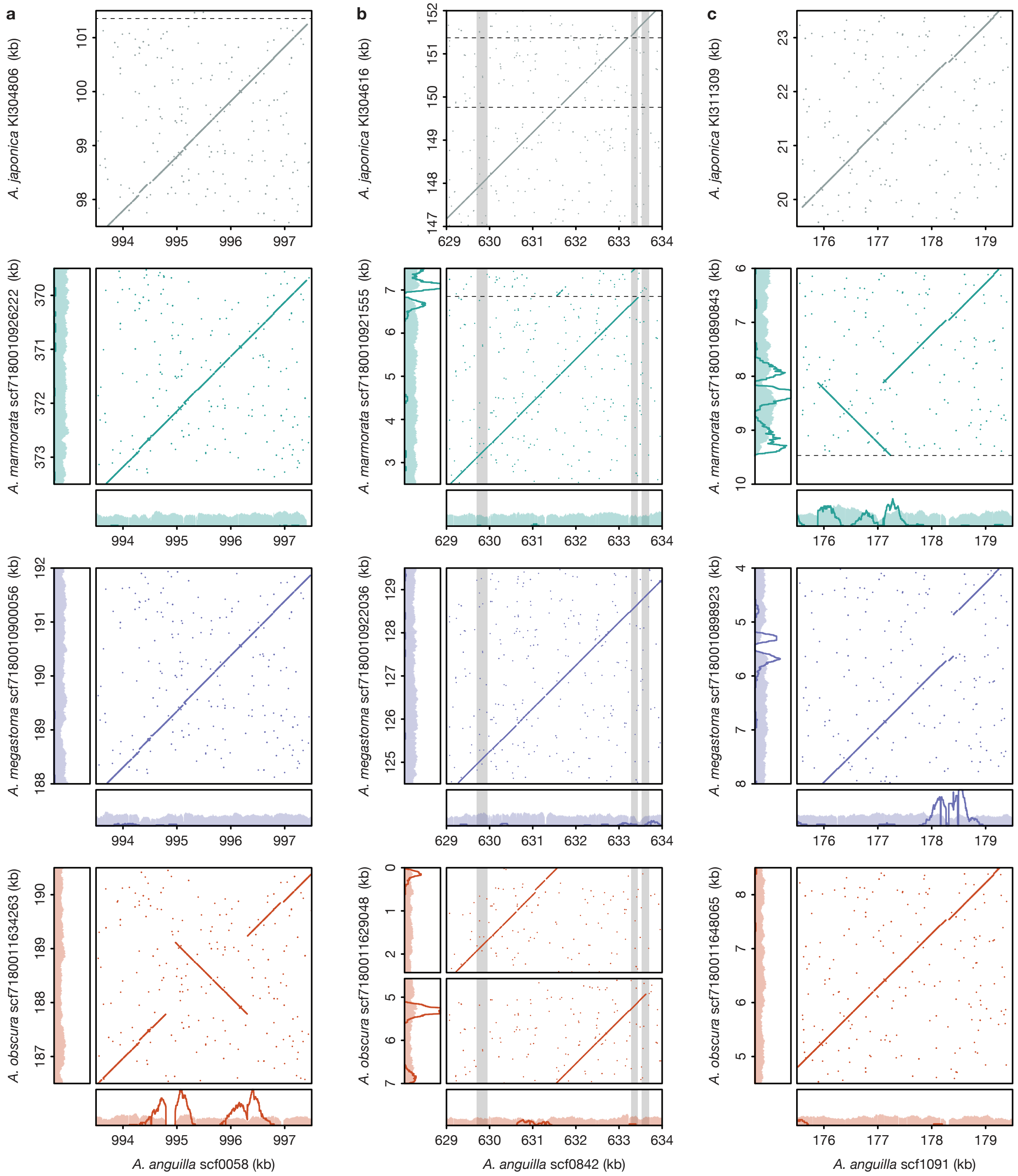

Possible inversions and transpositions between eel genome assemblies are visualized as dot plots, in which each dot represents a tuple of nine nucleotides that are identical or the reverse complement between the two scaffolds on the x- and y-axes. Dot plots are shown for each comparison of eight different regions on scaffolds of the *A. anguilla* reference genome assembly [28] (columns; **a-h**) and the corresponding scaffolds in each of the four genome assemblies for *A. japonica* [21] (gray; top row), *A. marmorata* (cyan; second row from top), *A. megastoma* (purple; third row from top), and *A. obscura* (red; bottom row). The eight regions on *A. anguilla* scaffolds were identified as candidate regions for rearrangements through a combination of automated and manual approaches as described in Supplementary Notes 5-6 and Supplementary Table 11. In the absence of rearrangements, dots are expected to fall along a single diagonal line, whereas transpositions produce multiple lines with the same orientation, and inversions lead to a combination of lines with different orientations. However, errors in genome assemblies can also produce these very same patterns.

Below each dot plot, we show in light colors the depths of *A. marmorata*, *A. megastoma*, or *A. obscura* reads mapped to the *A. anguilla* scaffold; read data of *A. japonica* were not available. These depth distributions are scaled so that the top of the plot corresponds to 200 reads. The solid line in the same plot shows the distribution of reads without proper pairing of mates (e.g. both mates sharing the same orientation or being at an unexpected distance to each other). These reads without proper pairing are expected from genomic rearrangements, close to the boundaries of the rearrangements. Distributions of reads without proper pairing are scaled so that the top of the plot corresponds to 50 reads.

To the left of each dot plot, we also show overall read depths (again in light colors and scaled to a maximum of 200 reads) and distributions of reads without proper pairing (again as a solid line and scaled to a maximum of 50 reads); however, in these cases for reads mapped to scaffold of the *A. marmorata*, *A. megastoma*, or *A. obscura* assemblies.

True rearrangments between *A. anguilla* and *A. marmorata*, *A. megastoma*, or *A. obscura* are expected to produce peaks in the distributions of reads without proper pairing when *A. marmorata*, *A. megastoma*, or *A. obscura* reads are mapped to the *A. anguilla* assembly, but no such peaks when the same reads are mapped to the species-specific assembly. In contrast, errors in the assemblies for *A. marmorata*, *A. megastoma*, or *A. obscura* are expected to produce peaks in the distributions of reads without proper pairing when reads are mapped to the species-specific assembly.

Dashed horizontal lines demarcate contig boundaries on *A. japonica*, *A. marmorata*, *A. megastoma*, and *A. obscura* scaffolds. Gray rectangles indicate coding sequences on *A. anguilla* scaffolds according to the gene prediction with AUGUSTUS.

a-c) Potential rearrangements mapping to scaffolds scf0058 (a), scf0842 (b), and scf1091 (c) of the *A. anguilla* reference genome assembly [28]. The absence of reads without proper pairing on the *A. obscura* scaffold scf7180011634263, together with peaks of these reads on the *A. anguilla* scaffold scf0058 confirms the presence of an inversion between these two scaffolds (a).

Supplementary Figure 21 (continued): Genomic rearrangements supported by WGS data.

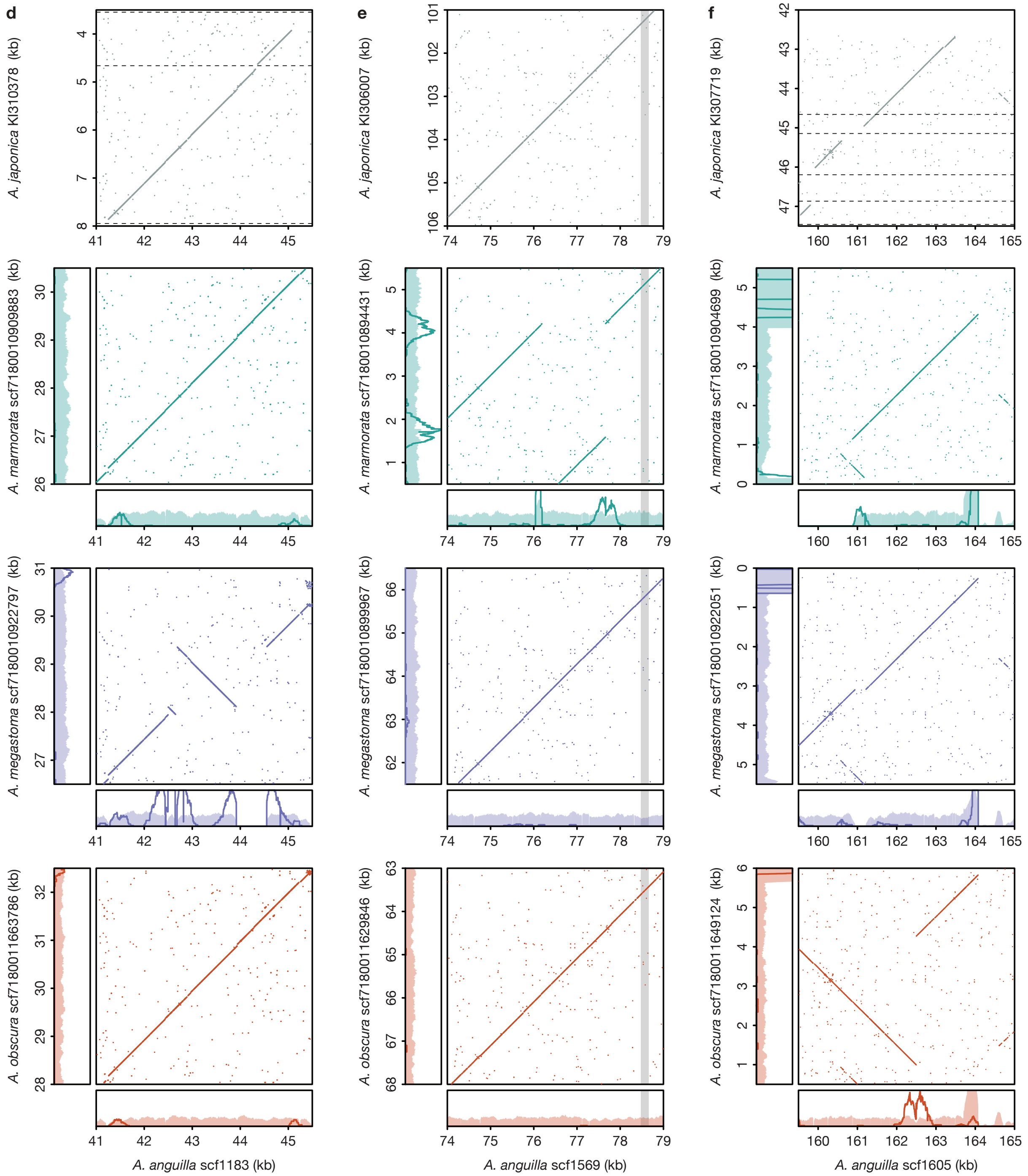

d-f) As a-c) but showing potential rearrangements mapping to *A. anguilla* scaffolds scf1183 (d), scf1569 (e), and scf1605 (f). Distributions of reads without proper pairing confirm the presence of an inversion between *A. megastoma* scaffold scf7180010922797 and *A. anguilla* scaffolds scf1183 (d).

Supplementary Figure 21 (continued): Genomic rearrangements supported by WGS data.

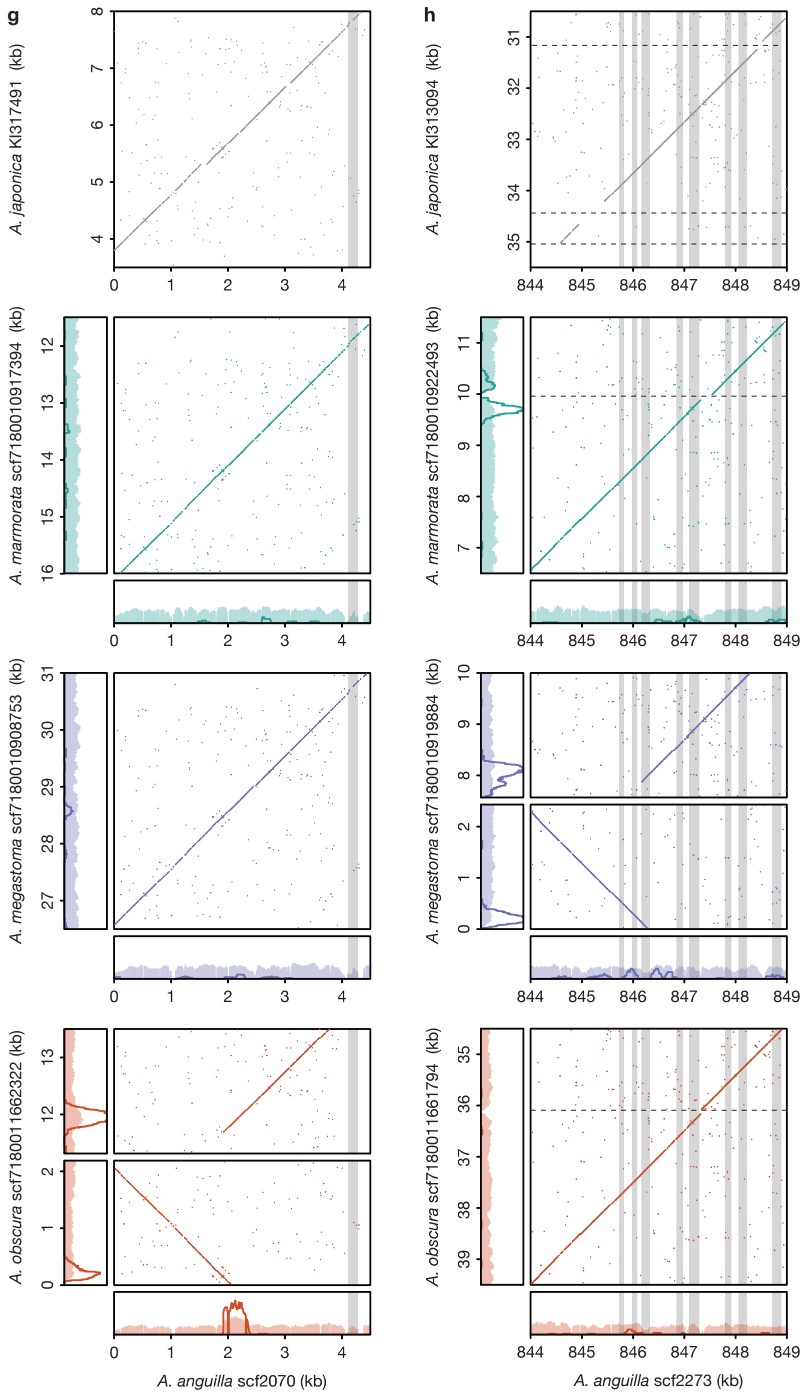

g-h) As a-f) but showing potential rearrangements mapping to *A. anguilla* scaffolds scf2070 (g) and scf2273 (h). Inversions between *A. obscura* scaffold scf7180011662322 and *A. anguilla* scaffold scf2070 (g) and between *A. megastoma* scaffold scf7180010919884 and *A. anguilla* scaffold scf2273 (h) may be present heterozygously, as shown in Supplementary Figure 22.

Supplementary Figure 22: Evidence for heterozygous presence of inversions.

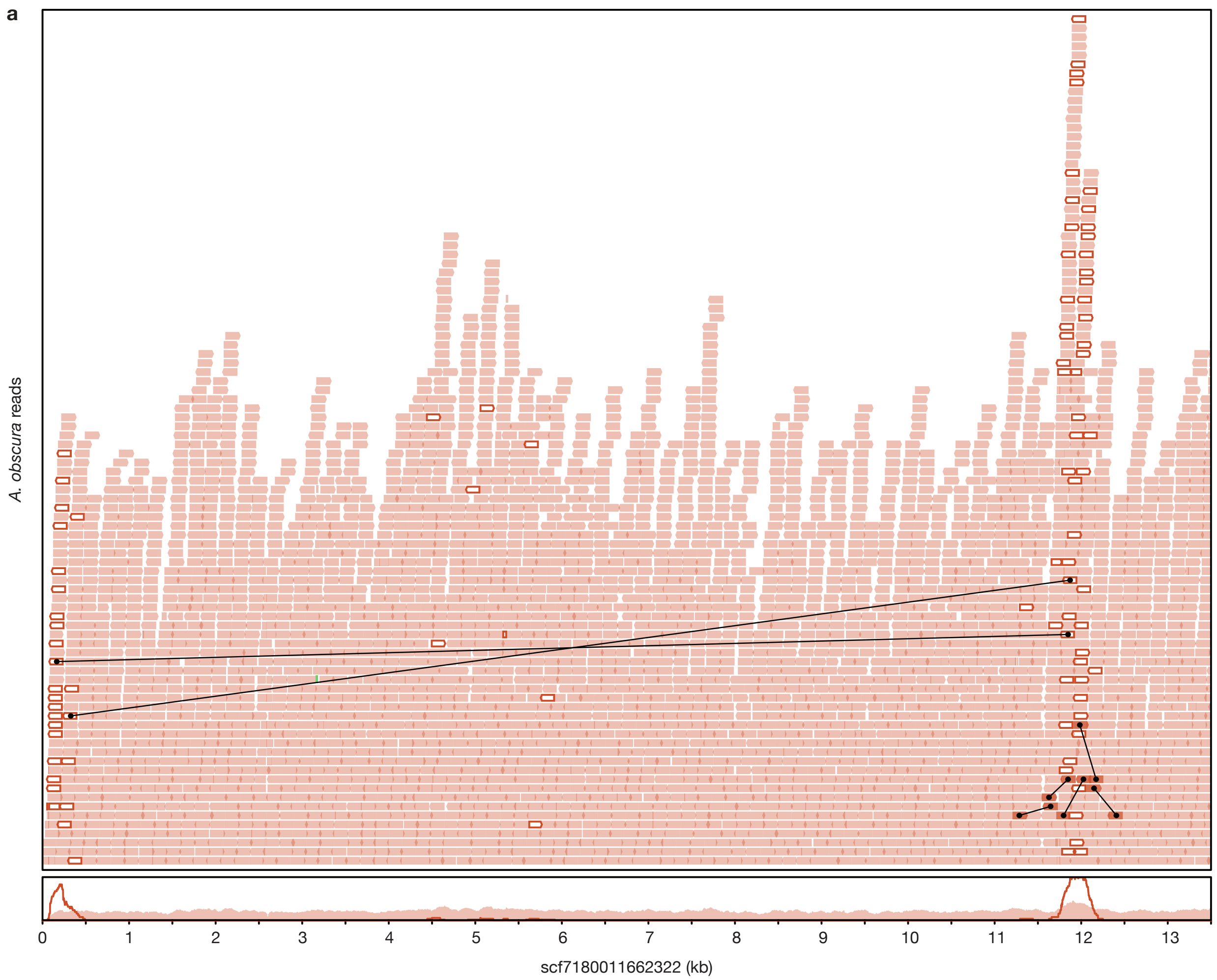

a) *A. obscura* reads mapped to scaffold scf7180011662322 of the *A. obscura* genome assembly, visualized with Integrative Genome Viewer (v.2.3.55) [45]. Reads with proper pairing are shown in light colors; those without proper pairing are shown with solid outlines. The smaller plot below shows the overall read depth (in light color) and the distribution of reads without proper pairing (as a solid line) as in Supplementary Figure 21g. Most of the reads without proper pairing that map to the reverse strand (i.e. reads that point to the left in the illustration) of *A. anguilla* scaffold scf2070 around position 12 kb have mates that also map to the reverse strand, around position 0-0.5 kb. Two examples of such mate pairs are connected with black lines to illustrate the long-distance pairing. The frequency of these pairs could indicate misassembly of the *A. obscura* scaffold; however, properly paired reads are also present across the potential breakpoint around position 12 kb. Examples of such properly paired mates are also connected with black lines. This combination of reads with and without proper pairing indicates that the *A. obscura* individual from which WGS data was obtained was heterozygous for an inversion mapping to the first 12 kb of *A. anguilla* scaffold scf2070.

Supplementary Figure 22 (continued): Evidence for heterozygous presence of inversions.

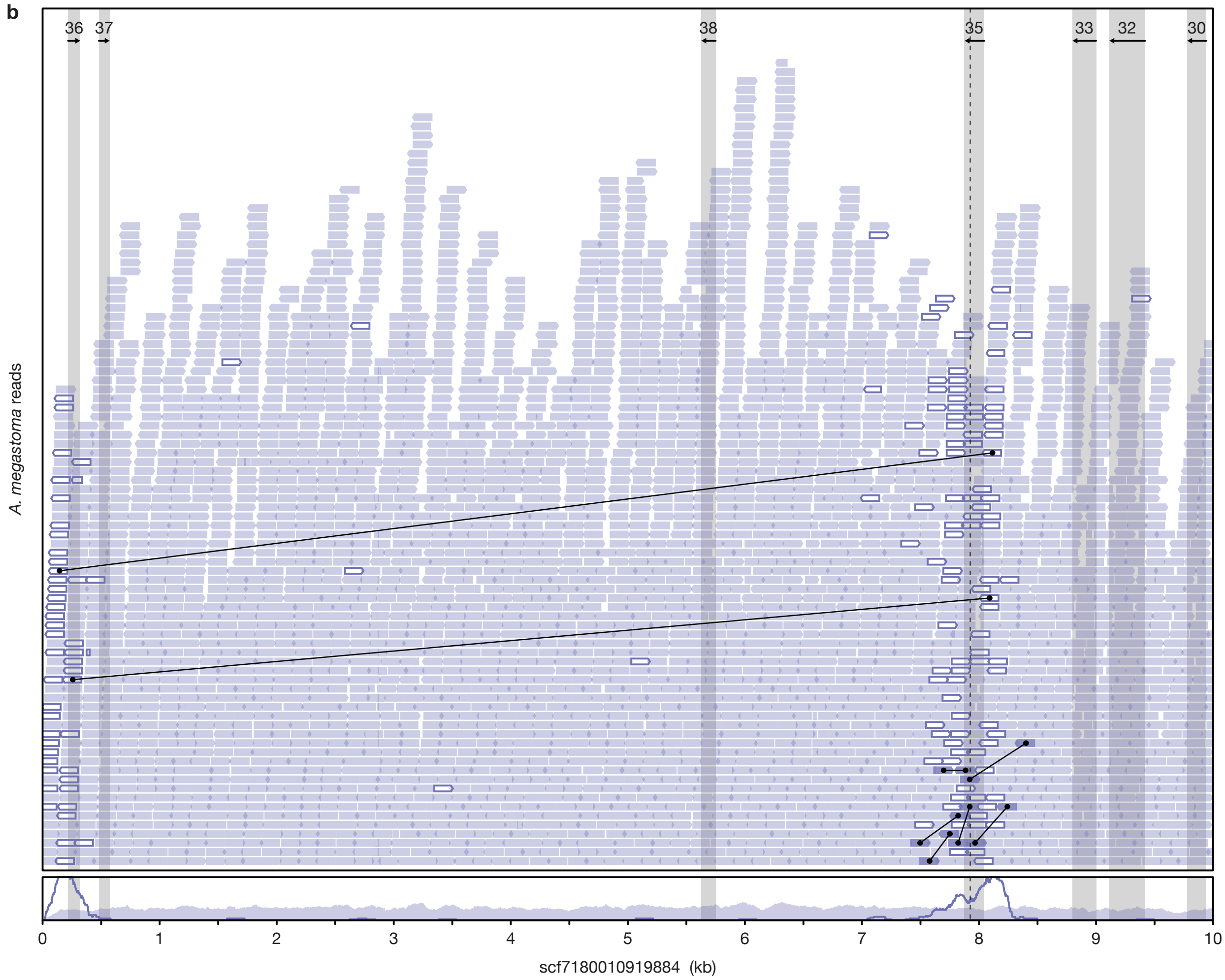

b) As a) but showing *A. megastoma* reads mapped to scaffold scf7180010919884 of the *A. megastoma* assembly. As in a) the combination of reads with and without proper pairing suggests that the *A. megastoma* individual from which WGS data was obtained was heterozygous for an inversion mapping to the first 8 kb of *A. anguilla* scaffold scf2273. Unlike in a), the distinct distributions of reads without proper pairing on the forward and reverse strands allow us to pinpoint the inversion breakpoint, shown as a dashed line. Gray rectangles indicate regions matching exon sequences of the zebrafish (*Danio rerio*) *myhc4* gene (NCBI accession NM_001020485; TBLASTX *e*-values < E-10; see Supplementary Table 12). Exon numbers and orientation are shown at the top of the figure.

Supplementary Tables

Supplementary Table 1: Sampled specimens.
Underlined specimens were included in the "core" group of individuals for *A. marmorata*, *A. megastoma*, and *A. obscura*, based on morphological measurements characteristic for the species. Species assignment is based on mitochondrial sequence data, and additionally on morphological measurements when these were available. Solomon Islands sampling sites SOK (Kolombangara), SOL (Kolombangara), SON (Nggatokae), SOR (Ranongga), and SOV (Vangunu) are jointly labeled "SO" in Figure 1, Supplementary Figure 4a, and descriptions in the text. The availability of morphological information is indicated in the morphology column. Unless specified otherwise, all specimens for which morphology information was available were included in morphological principal component analysis. [1]specimen removed due to read number below 600,000; [2]specimen not included in morphological principal component analysis even though morphological information is available; [3]specimen removed due to percentage of mapped reads below 70%.

Specimen ID	Species	Site	Lat.	Lon.	Date	# reads	Morphology
AFC09022	*A. marmorata*	AFC	-33.615	25.667	2009/03/22	6,776,718	partial
AFC09027	*A. marmorata*	AFC	-33.615	25.667	2009/03/22	4,634,730	partial
AFC09028	*A. marmorata*	AFC	-33.615	25.667	2009/03/22	4,693,084	partial
AFC09038	*A. marmorata*	AFC	-33.615	25.667	2009/03/22	2,400,252	partial
AFC09040	*A. marmorata*	AFC	-33.615	25.667	2009/03/22	4,033,770	partial
AFC09042	*A. marmorata*	AFC	-33.615	25.667	2009/03/22	6,358,602	partial
AFC09046	*A. marmorata*	AFC	-33.615	25.667	2009/03/22	5,043,994	partial
AFC09050	*A. marmorata*	AFC	-33.615	25.667	2009/03/22	3,329,484	partial
AFC09131	*A. marmorata*	AFC	-33.046	26.662	2009/04/13	8,918,104	partial
AFC09136	*A. marmorata*	AFC	-33.046	26.662	2009/04/13	4,789,220	partial
AFC09169	*A. marmorata*	AFC	-33.615	25.667	2009/03/22	183,024[1]	partial
AFC09192	*A. marmorata*	AFC	-33.615	25.667	2009/03/22	6,797,310	partial
AFC09250	*A. marmorata*	AFC	-33.615	25.667	2009/03/22	8,638,656	partial
AFC09269	*A. marmorata*	AFC	-33.615	25.667	2009/03/22	1,300,408	partial
AFC09282	*A. marmorata*	AFC	-33.615	25.667	2009/03/22	1,999,244	partial
AFS03068	*A. marmorata*	AFS	-26.713	31.979	2003/06/10	2,945,220	no
BOU15001	*A. megastoma*	BOU	-6.080	155.227	2015/04/04	10,524,168	yes
BOU15002	*A. megastoma*	BOU	-6.080	155.227	2015/04/04	8,753,268	yes
BOU15003	*A. megastoma*	BOU	-6.080	155.227	2015/04/04	12,765,510	yes
BOU15004	*A. megastoma*	BOU	-6.080	155.227	2015/04/04	8,763,996	yes
BOU15005	*A. megastoma*	BOU	-6.080	155.227	2015/04/05	6,346,666	yes[2]
BOU15006	*A. marmorata*	BOU	-5.982	155.365	2015/04/09	9,606,592	yes
BOU15007	*A. marmorata*	BOU	-5.982	155.365	2015/04/09	7,034,622	yes
BOU15009	*A. marmorata*	BOU	-5.982	155.365	2015/04/10	14,488,768	yes
BOU15010	*A. marmorata*	BOU	-5.982	155.365	2015/04/10	6,070,202	yes
BOU15011	*A. marmorata*	BOU	-5.982	155.365	2015/04/10	12,244,134	yes
BOU15012	*A. marmorata*	BOU	-5.982	155.365	2015/04/10	3,809,812	yes
BOU15013	*A. marmorata*	BOU	-5.982	155.365	2015/04/10	2,920,434	yes[2]
BOU15014	*A. marmorata*	BOU	-5.982	155.365	2015/04/10	4,069,002	yes
BOU15015	*A. marmorata*	BOU	-5.982	155.365	2015/04/10	4,142,280	yes
BOU15016	*A. marmorata*	BOU	-5.982	155.365	2015/04/10	8,406,776	yes

Supplementary Table 1 (continued)

Specimen ID	Species	Site	Lat.	Lon.	Date	# reads	Morphology
BOU15017	*A. marmorata*	BOU	-5.982	155.365	2015/04/11	6,477,100	yes
BOU15018	*A. marmorata*	BOU	-5.982	155.365	2015/04/12	16,346,782	yes
BOU15019	*A. marmorata*	BOU	-5.982	155.365	2015/04/12	3,438,714	yes
BOU15020	*A. marmorata*	BOU	-5.982	155.365	2015/04/12	4,981,164	yes
BOU15021	*A. marmorata*	BOU	-5.982	155.365	2015/04/12	1,490,284	yes
BOU15022	*A. marmorata*	BOU	-5.982	155.365	2015/04/13	2,212,684	yes
BOU15023	*A. marmorata*	BOU	-5.982	155.365	2015/04/13	4,800,140	yes
BOU15024	*A. marmorata*	BOU	-5.982	155.365	2015/04/12	12,316,926	yes
BOU15025	*A. marmorata*	BOU	-5.982	155.365	2015/04/14	1,569,478	yes
BOU15027	*A. megastoma*	BOU	-5.982	155.365	2015/04/14	4,104,044	yes
BOU15028	*A. marmorata*	BOU	-5.982	155.365	2015/04/14	3,353,376	yes
BOU15029	*A. marmorata*	BOU	-5.982	155.365	2015/04/14	715,048	yes
BOU15030	*A. megastoma*	BOU	-5.982	155.365	2015/04/14	2,705,922	yes
BOU15031	*A. marmorata*	BOU	-5.982	155.365	2015/04/14	4,074,020	yes
BOU15032	*A. marmorata*	BOU	-5.982	155.365	2015/04/12	8,950,896	yes
JAV11001	*A. marmorata*	JAV	-7.031	106.543	2011/06/NA	1,911,324	no
JAV11002	*A. marmorata*	JAV	-7.031	106.543	2011/06/NA	7,555,770	no
JAV11003	*A. marmorata*	JAV	-7.031	106.543	2011/06/NA	1,090,534	no
JAV11004	*A. marmorata*	JAV	-7.031	106.543	2011/06/NA	8,046,460	no
JAV11005	*A. marmorata*	JAV	-7.031	106.543	2011/06/NA	2,310,702	no
JAV11006	*A. marmorata*	JAV	-7.031	106.543	2011/06/NA	2,258,760	no
JAV11007	*A. bicolor*	JAV	-7.031	106.543	2011/06/NA	2,688,928	no
JAV11008	*A. marmorata*	JAV	-7.031	106.543	2011/06/NA	4,625,680	no
JAV11009	*A. marmorata*	JAV	-7.031	106.543	2011/06/NA	1,036,900	no
JAV11010	*A. interioris*	JAV	-7.031	106.543	2011/06/NA	6,425,584	no
JAV11011	*A. marmorata*	JAV	-7.031	106.543	2011/06/NA	11,251,146	no
JAV11012	*A. marmorata*	JAV	-7.031	106.543	2011/06/NA	6,491,164	no
JAV11013	*A. marmorata*	JAV	-7.031	106.543	2011/06/NA	5,513,280	no
JAV11014	*A. marmorata*	JAV	-7.031	106.543	2011/06/NA	9,067,812	no
JAV11015	*A. bicolor*	JAV	-7.031	106.543	2011/06/NA	1,363,586	no
JAV11016	*A. bicolor*	JAV	-7.031	106.543	2011/06/NA	2,046,036	no
JAV11017	*A. marmorata*	JAV	-7.031	106.543	2011/06/NA	1,643,646	no
JAV11018	*A. marmorata*	JAV	-7.031	106.543	2011/06/NA	7,677,132	no
JAV11019	*A. marmorata*	JAV	-7.031	106.543	2011/06/NA	738,182	no
JAV11020	*A. marmorata*	JAV	-7.031	106.543	2011/06/NA	272,336[1]	no
JAV11021	*A. marmorata*	JAV	-7.031	106.543	2011/06/NA	4,231,276	no
JAV11022	*A. bicolor*	JAV	-7.031	106.543	2011/06/NA	11,179,098	no
JAV11023	*A. interioris*	JAV	-7.031	106.543	2011/06/NA	5,903,968	no
JAV11024	*A. marmorata*	JAV	-7.031	106.543	2011/06/NA	3,015,256	no
JAV11025	*A. marmorata*	JAV	-7.031	106.543	2011/06/NA	1,471,420	no
JAV11026	*A. marmorata*	JAV	-7.031	106.543	2011/06/NA	406,532[1]	no
JAV11027	*A. marmorata*	JAV	-7.031	106.543	2011/06/NA	1,378,514	no
JAV11028	*A. marmorata*	JAV	-7.031	106.543	2011/06/NA	1,400,300	no
JAV11029	*A. marmorata*	JAV	-7.031	106.543	2011/06/NA	374,992[1]	no
JAV11030	*A. marmorata*	JAV	-7.031	106.543	2011/06/NA	1,060,828	no
MAY03001	*A. marmorata*	MAY	-12.736	45.173	2003/11/09	3,635,428	no
MAY03003	*A. marmorata*	MAY	-12.736	45.173	2003/11/09	4,765,700	no

Supplementary Table 1 (continued)

Specimen ID	Species	Site	Lat.	Lon.	Date	# reads	Morphology
MAY03005	*A. marmorata*	MAY	-12.736	45.173	2003/11/09	4,158,434	no
MAY03006	*A. marmorata*	MAY	-12.736	45.173	2003/11/11	2,241,702	no
MAY03007	*A. marmorata*	MAY	-12.736	45.173	2003/11/09	1,725,756	no
MAY03009	*A. marmorata*	MAY	-12.736	45.173	2003/11/09	5,806,712	no
MAY03013	*A. marmorata*	MAY	-12.736	45.173	2003/11/09	2,181,048	no
MAY03017	*A. marmorata*	MAY	-12.736	45.173	2003/11/11	3,854,094	no
MAY03018	*A. marmorata*	MAY	-12.736	45.173	2003/11/09	2,476,808	no
MAY03019	*A. marmorata*	MAY	-12.736	45.173	2003/11/09	2,426,188	no
MAY03020	*A. marmorata*	MAY	-12.736	45.173	2003/11/09	1,422,048	no
MAY03021	*A. marmorata*	MAY	-12.736	45.173	2003/11/11	3,243,600	no
MAY03022	*A. marmorata*	MAY	-12.736	45.173	2003/11/11	4,022,620	no
MAY03023	*A. marmorata*	MAY	-12.736	45.173	2003/11/09	3,288,808	no
MAY03024	*A. marmorata*	MAY	-12.736	45.173	2003/11/11	3,669,560	no
MAY03025	*A. marmorata*	MAY	-12.736	45.173	2003/11/09	3,129,952	no
MAY03027	*A. marmorata*	MAY	-12.736	45.173	2003/11/11	2,452,074	no
MAY03028	*A. marmorata*	MAY	-12.736	45.173	2003/11/11	2,337,216	no
NCA16001	*A. marmorata*	NCA	-21.305	165.025	2016/07/27	1,463,126	yes
NCA16002	*A. marmorata*	NCA	-21.305	165.025	2016/07/27	3,965,922	yes
NCA16003	*A. marmorata*	NCA	-21.305	165.025	2016/07/27	885,746	yes
NCA16004	*A. marmorata*	NCA	-21.305	165.025	2016/07/27	3,202,396	partial
NCA16005	*A. marmorata*	NCA	-21.305	165.025	2016/07/27	3,506,424	partial
NCA16006	*A. marmorata*	NCA	-21.305	165.025	2016/07/27	6,456,506	partial
NCA16007	*A. marmorata*	NCA	-21.305	165.025	2016/07/27	4,056,230	partial
NCA16008	*A. marmorata*	NCA	-21.305	165.025	2016/07/27	2,850,918	partial
NCA16009	*A. marmorata*	NCA	-21.305	165.025	2016/07/27	4,937,224	partial
NCA16010	*A. marmorata*	NCA	-21.305	165.025	2016/07/27	4,202,782	no
NCA16011	*A. marmorata*	NCA	-21.305	165.025	2016/07/27	4,716,276	no
NCA16014	*A. marmorata*	NCA	-21.305	165.025	2016/07/27	4,179,094	partial
NCA16015	*A. obscura*	NCA	-21.305	165.025	2016/07/27	1,913,782	partial
NCA16018	*A. marmorata*	NCA	-21.302	165.029	2016/07/28	7,270,454	partial
NCA16020	*A. marmorata*	NCA	-21.302	165.029	2016/07/28	6,578,652	partial
NCA16021	*A. marmorata*	NCA	-21.302	165.029	2016/07/28	3,054,210	partial
NCA16022	*A. marmorata*	NCA	-21.302	165.029	2016/07/28	2,739,816	partial
NCA16023	*A. marmorata*	NCA	-21.302	165.029	2016/07/28	6,474,304	yes
NCA16024	*A. marmorata*	NCA	-21.302	165.029	2016/07/28	6,085,708	partial
NCA16025	*A. marmorata*	NCA	-21.302	165.029	2016/07/28	1,234,354	partial
NCA16027	*A. marmorata*	NCA	-21.302	165.029	2016/07/28	7,005,388	yes
NCA16028	*A. marmorata*	NCA	-21.302	165.029	2016/07/28	5,545,016	yes
NCA16030	*A. marmorata*	NCA	-21.302	165.029	2016/07/28	4,546,024	partial
NCA16031	*A. marmorata*	NCA	-20.491	164.258	2016/08/02	4,881,226	partial
NCA16034	*A. marmorata*	NCA	-20.491	164.258	2016/08/02	1,596,114	partial
NCA16035	*A. marmorata*	NCA	-20.491	164.258	2016/08/02	4,372,818	yes
NCA16036	*A. marmorata*	NCA	-20.491	164.258	2016/08/02	1,913,554	yes
NCA16039	*A. marmorata*	NCA	-20.491	164.258	2016/08/02	1,995,702	partial
NCA16041	*A. marmorata*	NCA	-20.491	164.258	2016/08/02	3,543,860	yes
NCA16042	*A. marmorata*	NCA	-20.491	164.258	2016/08/02	5,045,656	partial
NCA16043	*A. marmorata*	NCA	-20.491	164.258	2016/08/02	2,637,300	yes

Supplementary Table 1 (continued)

Specimen ID	Species	Site	Lat.	Lon.	Date	# reads	Morphology
NCA16044	*A. marmorata*	NCA	-20.491	164.258	2016/08/02	5,381,242	partial
NCA16045	*A. marmorata*	NCA	-20.491	164.258	2016/08/02	3,630,832	yes
NCA16046	*A. marmorata*	NCA	-22.111	166.423	2016/08/09	4,248,040	yes
NCA16049	*A. marmorata*	NCA	-22.111	166.423	2016/08/09	4,212,004	yes
NCA16053	*A. marmorata*	NCA	-22.136	166.367	2016/08/09	3,653,266	yes
NCA16056	*A. marmorata*	NCA	-22.136	166.367	2016/08/09	6,829,734	partial
NCA16063	*A. marmorata*	NCA	-22.136	166.367	2016/08/09	931,716	yes[2]
NCA16064	*A. marmorata*	NCA	-22.136	166.367	2016/08/09	4,739,518	yes
NCA16084	*A. marmorata*	NCA	-21.749	166.084	2016/08/11	5,874,506	yes
NCA16099	*A. marmorata*	NCA	-21.749	166.084	2016/08/11	7,063,454	yes
NCA16110	*A. marmorata*	NCA	-22.035	166.208	2016/08/18	4,691,496	partial
NCA16116	*A. marmorata*	NCA	-22.035	166.208	2016/08/18	4,702,550	yes
NCA16117	*A. marmorata*	NCA	-22.035	166.208	2016/08/18	4,790,108	yes
NCA16120	*A. marmorata*	NCA	-22.038	166.220	2016/08/18	6,610,270	partial
PHC08C01	*A. luzonensis*	PHC	18.355	121.634	2008/09/26	4,385,110	no
PHC08C02	*A. luzonensis*	PHC	18.355	121.634	2008/09/26	2,067,082	no
PHC08C03	*A. luzonensis*	PHC	18.355	121.634	2008/09/26	6,693,386	no
PHC08C04	*A. luzonensis*	PHC	18.355	121.634	2008/09/26	7,107,044	no
PHC08C05	*A. luzonensis*	PHC	18.355	121.634	2008/09/26	8,824,514	no
PHC08C06	*A. luzonensis*	PHC	18.355	121.634	2008/09/26	9,469,068	no
PHC08C07	*A. luzonensis*	PHC	18.355	121.634	2008/09/26	6,707,486	no
PHC08C08	*A. luzonensis*	PHC	18.355	121.634	2008/09/26	176,638[1]	no
PHC08C09	*A. luzonensis*	PHC	18.355	121.634	2008/09/26	6,465,580	no
PHC08C10	*A. marmorata*	PHC	18.355	121.634	2008/09/26	6,767,542	no
PHC08C11	*A. luzonensis*	PHC	18.355	121.634	2008/09/26	11,154,032	no
PHC08C12	*A. luzonensis*	PHC	18.355	121.634	2008/09/26	6,774,270	no
PHC08C13	*A. luzonensis*	PHC	18.355	121.634	2008/09/26	5,412,554	no
PHC08C15	*A. luzonensis*	PHC	18.355	121.634	2008/09/26	3,325,444	no
PHC08C16	*A. luzonensis*	PHC	18.355	121.634	2008/09/26	3,157,590	no
PHC08C17	*A. luzonensis*	PHC	18.355	121.634	2008/09/26	35,658[1]	no
PHC08C18	*A. luzonensis*	PHC	18.355	121.634	2008/09/26	5,386,382	no
PHC08C19	*A. marmorata*	PHC	18.355	121.634	2008/09/26	301,666[1]	no
PHC08C20	*A. marmorata*	PHC	18.355	121.634	2008/09/26	4,533,308	no
PHC08C21	*A. marmorata*	PHC	18.355	121.634	2008/09/26	50,532[1]	no
PHC08C22	*A. luzonensis*	PHC	18.355	121.634	2008/09/26	6,089,432	no
PHC08C23	*A. luzonensis*	PHC	18.355	121.634	2008/09/26	58,386[1]	no
PHC08C24	*A. luzonensis*	PHC	18.355	121.634	2008/09/26	778,644	no
PHC08C25	*A. marmorata*	PHC	18.355	121.634	2008/09/26	3,036,944	no
PHC08C26	*A. luzonensis*	PHC	18.355	121.634	2008/09/26	8,041,488	no
PHC08C27	*A. luzonensis*	PHC	18.355	121.634	2008/09/26	3,149,128	no
PHC08C28	*A. luzonensis*	PHC	18.355	121.634	2008/09/26	1,770,520	no
PHC08C29	*A. luzonensis*	PHC	18.355	121.634	2008/09/26	1,787,774	no
PHC08P20	*A. marmorata*	PHC	18.355	121.634	2008/09/26	3,961,354	no
PHC08P22	*A. marmorata*	PHC	18.355	121.634	2008/09/26	4,094,926	no
PHC08P23	*A. marmorata*	PHC	18.355	121.634	2008/09/26	5,894,520	no
PHP14P01	*A. marmorata*	PHP	7.835	123.509	2014/02/14	4,783,294	no
PHP14P02	*A. marmorata*	PHP	7.835	123.509	2014/02/14	5,889,742	no

Supplementary Table 1 (continued)

Specimen ID	Species	Site	Lat.	Lon.	Date	# reads	Morphology
PHP14P03	*A. marmorata*	PHP	7.835	123.509	2014/02/14	6,371,944	no
PHP14P04	*A. marmorata*	PHP	7.835	123.509	2014/02/14	4,141,222	no
PHP14P05	*A. marmorata*	PHP	7.835	123.509	2014/02/14	5,544,038	no
PHP14P06	*A. marmorata*	PHP	7.835	123.509	2014/02/14	2,983,648	no
PHP14P07	*A. marmorata*	PHP	7.835	123.509	2014/02/14	4,178,472	no
PHP14P08	*A. marmorata*	PHP	7.835	123.509	2014/02/14	3,034,380	no
PHP14P09	*A. marmorata*	PHP	7.835	123.509	2014/02/14	4,966,488	no
PHP14P10	*A. marmorata*	PHP	7.835	123.509	2014/02/14	5,286,256	no
PHP14P11	*A. marmorata*	PHP	7.835	123.509	2014/02/14	6,830,854	no
PHP14P12	*A. marmorata*	PHP	7.835	123.509	2014/02/14	3,904,972	no
PHP14P13	*A. marmorata*	PHP	7.835	123.509	2014/02/14	3,827,650	no
PHP14P14	*A. marmorata*	PHP	7.835	123.509	2014/02/14	5,505,830	no
PHP14P15	*A. marmorata*	PHP	7.835	123.509	2014/02/14	2,567,042	no
PHP14P16	*A. marmorata*	PHP	7.835	123.509	2014/02/14	770,766	no
PHP14P17	*A. marmorata*	PHP	7.835	123.509	2014/02/14	4,868,568	no
PHP14P18	*A. marmorata*	PHP	7.835	123.509	2014/02/14	4,753,592	no
PHP14P19	*A. marmorata*	PHP	7.835	123.509	2014/02/14	4,047,700	no
PHP14P21	*A. marmorata*	PHP	7.835	123.509	2014/02/14	1,969,502	no
PHP14P24	*A. marmorata*	PHP	7.835	123.509	2014/02/14	6,280,804	no
PHP14P25	*A. marmorata*	PHP	7.835	123.509	2014/02/14	6,543,116	no
PHP14P26	*A. marmorata*	PHP	7.835	123.509	2014/02/14	7,983,072	no
PHP14P27	*A. marmorata*	PHP	7.835	123.509	2014/02/14	9,373,838	no
PHP14P28	*A. marmorata*	PHP	7.835	123.509	2014/02/14	8,095,200	no
PHP14P29	*A. marmorata*	PHP	7.835	123.509	2014/02/14	6,411,040	no
PHP14P30	*A. marmorata*	PHP	7.835	123.509	2014/02/14	6,390,714	no
REU01002	*A. marmorata*	REU	-20.983	55.685	2001/02/05	3,454,510	no
REU01014	*A. marmorata*	REU	-20.983	55.685	2001/02/05	2,737,978	no
REU01016	*A. marmorata*	REU	-20.983	55.685	2001/02/05	1,923,798	no
REU03004	*A. marmorata*	REU	-20.983	55.685	2003/11/04	3,841,652	no
REU03008	*A. marmorata*	REU	-20.983	55.685	2003/11/04	3,107,032	no
REU03010	*A. marmorata*	REU	-20.912	55.630	2003/11/04	4,676,038	no
REU03011	*A. marmorata*	REU	-20.983	55.685	2003/11/04	4,215,150	no
REU03012	*A. marmorata*	REU	-20.912	55.630	2003/11/04	8,125,876	no
REU03015	*A. marmorata*	REU	-20.912	55.630	2003/11/04	3,492,136	no
REU03026	*A. mossambica*	REU	-20.912	55.630	2003/11/04	3,450,942	no
SAA16001	*A. marmorata*	SAA	-14.304	170.816	2016/08/18	7,116,822	partial
SAA16002	*A. marmorata*	SAA	-14.304	170.816	2016/08/19	7,015,912	partial
SAA16003	*A. marmorata*	SAA	-14.304	170.816	2016/08/18	3,522,172	partial
SAA16004	*A. marmorata*	SAA	-14.304	170.816	2016/08/18	3,740,088	partial
SAA16005	*A. marmorata*	SAA	-14.304	170.816	2016/08/18	18,305,104	no
SAA16006	*A. marmorata*	SAA	-14.304	170.816	2016/08/18	6,540,862	partial
SAA16007	*A. marmorata*	SAA	-14.304	170.816	2016/08/18	3,685,986	partial
SAA16008	*A. marmorata*	SAA	-14.304	170.816	2016/08/18	5,828,106	partial
SAA16009	*A. marmorata*	SAA	-14.304	170.816	2016/08/18	5,979,728	partial
SAA16010	*A. marmorata*	SAA	-14.304	170.816	2016/08/18	1,269,150	partial
SAA16011	*A. marmorata*	SAA	-14.304	170.816	2016/08/18	3,557,730	partial
SAA16012	*A. marmorata*	SAA	-14.304	170.816	2016/08/18	7,436,372	partial

Supplementary Table 1 (continued)

Specimen ID	Species	Site	Lat.	Lon.	Date	# reads	Morphology
SAA16013	*A. marmorata*	SAA	-14.304	170.816	2016/08/18	2,752,316	partial
SAA16014	*A. megastoma*	SAA	-14.332	170.793	2016/08/19	3,018,560	partial
SAA16015	*A. megastoma*	SAA	-14.332	170.793	2016/08/19	5,022,542	partial
SAA16016	*A. marmorata*	SAA	-14.332	170.793	2016/08/19	7,564,634	partial
SAA16017	*A. marmorata*	SAA	-14.332	170.793	2016/08/19	881,890	partial
SAA16018	*A. marmorata*	SAA	-14.332	170.793	2016/08/19	6,003,128	partial
SAA16019	*A. marmorata*	SAA	-14.332	170.793	2016/08/19	1,456,892	partial
SAA16020	*A. marmorata*	SAA	-14.332	170.793	2016/08/19	4,554,650	partial
SAA16021	*A. marmorata*	SAA	-14.332	170.793	2016/08/19	1,433,016	partial
SAA16022	*A. marmorata*	SAA	-14.332	170.793	2016/08/19	5,381,110	partial
SAA16023	*A. marmorata*	SAA	-14.304	170.816	2016/08/20	5,759,626	partial
SAA16024	*A. marmorata*	SAA	-14.304	170.816	2016/08/20	7,833,688	partial
SAA16025	*A. marmorata*	SAA	-14.304	170.816	2016/08/20	3,393,344	partial
SAA16026	*A. marmorata*	SAA	-14.304	170.816	2016/08/20	6,973,470	partial
SAA16027	*A. marmorata*	SAA	-14.304	170.816	2016/08/20	8,727,666	partial
SAA16028	*A. marmorata*	SAA	-14.304	170.816	2016/08/20	12,240[1]	partial
SAA16029	*A. marmorata*	SAA	-14.304	170.816	2016/08/20	9,310,208	partial
SAA16030	*A. marmorata*	SAA	-14.304	170.816	2016/08/20	4,058,900	partial
SAA16031	*A. marmorata*	SAA	-14.304	170.816	2016/08/20	4,624,388	partial
SAA16032	*A. marmorata*	SAA	-14.304	170.816	2016/08/20	5,149,616	partial
SAA16033	*A. marmorata*	SAA	-14.304	170.816	2016/08/20	266,538[1]	partial
SAA16034	*A. marmorata*	SAA	-14.304	170.816	2016/08/20	7,292,490	partial
SAA16035	*A. marmorata*	SAA	-14.304	170.816	2016/08/20	6,989,336	partial
SAA16036	*A. marmorata*	SAA	-14.304	170.816	2016/08/20	7,190,652	partial
SAA16037	*A. marmorata*	SAA	-14.304	170.816	2016/08/20	58,520[1]	partial
SAA16038	*A. marmorata*	SAA	-14.304	170.816	2016/08/20	3,566,502	partial
SAW16001	*A. marmorata*	SAW	-13.836	171.765	2016/08/27	2,750,534	yes
SAW16002	*A. megastoma*	SAW	-13.904	171.575	2016/08/29	8,122,668	yes
SAW16003	*A. marmorata*	SAW	-13.874	171.651	2016/08/31	7,612,856	yes
SAW16004	*A. marmorata*	SAW	-13.874	171.651	2016/08/31	1,578,510	yes
SAW16005	*A. marmorata*	SAW	-13.874	171.651	2016/08/31	5,100,238	yes
SAW16006	*A. marmorata*	SAW	-13.874	171.651	2016/08/31	47,706[1]	yes[2]
SAW16007	*A. marmorata*	SAW	-13.874	171.651	2016/08/31	2,616,202	yes
SAW16008	*A. marmorata*	SAW	-13.874	171.651	2016/08/31	3,802,620	yes
SAW16009	*A. marmorata*	SAW	-13.874	171.651	2016/08/31	2,947,904	yes
SAW16010	*A. marmorata*	SAW	-13.874	171.651	2016/08/31	8,468,370	yes
SAW16011	*A. marmorata*	SAW	-13.874	171.651	2016/08/31	5,449,034	yes
SAW16012	*A. marmorata*	SAW	-13.874	171.651	2016/08/31	4,835,336	yes
SAW16013	*A. marmorata*	SAW	-13.874	171.651	2016/08/31	7,091,060	yes
SAW16014	*A. marmorata*	SAW	-13.874	171.651	2016/08/31	4,780,360	yes
SAW16015	*A. marmorata*	SAW	-13.874	171.651	2016/08/31	3,078,620	yes
SAW16016	*A. marmorata*	SAW	-13.874	171.651	2016/08/31	4,916,950	yes
SAW16017	*A. marmorata*	SAW	-13.874	171.651	2016/08/31	7,284,612	yes
SAW16018	*A. marmorata*	SAW	-13.874	171.651	2016/08/31	25,368[1]	yes[2]
SAW16019	*A. marmorata*	SAW	-13.874	171.651	2016/08/31	3,451,030	yes
SAW16020	*A. marmorata*	SAW	-13.874	171.651	2016/08/31	3,764,650	yes
SAW16021	*A. obscura*	SAW	-14.025	171.431	2016/09/01	8,156,690	yes

Supplementary Table 1 (continued)

Specimen ID	Species	Site	Lat.	Lon.	Date	# reads	Morphology
SAW16022	*A. obscura*	SAW	-14.025	171.431	2016/09/01	3,012,538	yes
SAW16023	*A. obscura*	SAW	-14.025	171.431	2016/09/01	4,522,382	yes
SAW16024	*A. obscura*	SAW	-14.025	171.431	2016/09/01	3,250,488	yes
SAW16025	*A. obscura*	SAW	-14.025	171.431	2016/09/01	2,821,526	yes
SAW16026	*A. obscura*	SAW	-14.025	171.431	2016/09/01	3,173,878	yes
SAW16027	*A. obscura*	SAW	-14.025	171.431	2016/09/01	2,975,754	yes[2]
SAW16028	*A. obscura*	SAW	-14.025	171.431	2016/09/01	2,657,622	yes
SAW16029	*A. obscura*	SAW	-14.025	171.431	2016/09/01	399,402[1]	yes[2]
SAW16030	*A. obscura*	SAW	-14.025	171.431	2016/09/01	6,092,366	yes
SAW16031	*A. obscura*	SAW	-14.025	171.431	2016/09/01	2,508,612	yes
SAW16032	*A. obscura*	SAW	-14.025	171.431	2016/09/01	4,808,164	yes
SAW16033	*A. obscura*	SAW	-14.025	171.431	2016/09/01	6,932,430	yes
SAW16034	*A. obscura*	SAW	-14.025	171.431	2016/09/01	2,710,582	yes
SAW16035	*A. obscura*	SAW	-14.025	171.431	2016/09/01	7,565,924	yes
SAW16036	*A. obscura*	SAW	-14.025	171.431	2016/09/01	6,343,662	yes
SAW16037	*A. obscura*	SAW	-14.025	171.431	2016/09/01	7,089,804	yes
SAW16038	*A. obscura*	SAW	-14.025	171.431	2016/09/01	5,230,320	yes
SAW16039	*A. obscura*	SAW	-14.025	171.431	2016/09/01	4,170,504	yes
SAW16040	*A. obscura*	SAW	-14.025	171.431	2016/09/01	9,956,058	yes[2]
SAW16041	*A. obscura*	SAW	-14.025	171.431	2016/09/01	5,220,798	yes
SAW16042	*A. obscura*	SAW	-14.025	171.431	2016/09/01	3,907,842	yes
SAW16043	*A. obscura*	SAW	-14.025	171.431	2016/09/01	451,282[1]	yes
SAW16044	*A. obscura*	SAW	-14.025	171.431	2016/09/01	696,640	yes
SAW16045	*A. obscura*	SAW	-14.025	171.431	2016/09/01	3,211,904	yes
SAW16046	*A. marmorata*	SAW	-13.876	171.639	2016/09/01	6,048,220	yes
SAW16047	*A. marmorata*	SAW	-13.876	171.639	2016/09/02	7,660,116	yes
SAW16048	*A. marmorata*	SAW	-13.876	171.639	2016/09/02	2,626,456	yes
SAW16049	*A. marmorata*	SAW	-13.876	171.639	2016/09/02	7,328,702	yes
SAW16050	*A. marmorata*	SAW	-13.876	171.639	2016/09/02	3,279,138	yes
SAW16051	*A. marmorata*	SAW	-13.876	171.639	2016/09/02	2,574,796	yes
SAW16052	*A. marmorata*	SAW	-13.876	171.639	2016/09/02	3,483,700	yes
SAW16054	*A. marmorata*	SAW	-13.876	171.639	2016/09/02	5,107,548	yes
SAW16055	*A. marmorata*	SAW	-13.876	171.639	2016/09/02	5,252,892	yes
SAW16056	*A. marmorata*	SAW	-13.876	171.639	2016/09/02	5,137,008	yes
SAW16057	*A. marmorata*	SAW	-13.876	171.639	2016/09/02	1,370,838	yes
SAW16058	*A. marmorata*	SAW	-13.876	171.639	2016/09/02	3,511,122	yes
SAW17B02	*A. marmorata*	SAW	-13.968	171.862	2017/02/23	5,261,166	no
SAW17B10	*A. megastoma*	SAW	-13.968	171.862	2017/02/24	5,240,736	yes
SAW17B13	*A. megastoma*	SAW	-13.968	171.862	2017/02/24	5,383,126	partial
SAW17B16	*A. obscura*	SAW	-13.978	171.860	2017/02/25	4,077,174	partial
SAW17B17	*A. obscura*	SAW	-13.978	171.860	2017/02/25	5,551,346	partial
SAW17B18	*A. obscura*	SAW	-13.978	171.860	2017/02/25	5,749,306	partial
SAW17B19	*A. obscura*	SAW	-13.978	171.860	2017/02/25	7,420,046	partial
SAW17B27	*A. marmorata*	SAW	-13.968	171.862	2017/02/27	7,417,178	partial
SAW17B48	*A. megastoma*	SAW	-14.026	171.714	2017/03/01	7,218,260	partial
SAW17B49	*A. marmorata*	SAW	-14.026	171.714	2017/03/01	3,919,036	partial
SAW17B55	*A. megastoma*	SAW	-13.992	171.588	2017/03/09	4,638,202	partial

Supplementary Table 1 (continued)

Specimen ID	Species	Site	Lat.	Lon.	Date	# reads	Morphology
SAW17B56	*A. marmorata*	SAW	-13.968	171.862	2017/02/27	6,826,590	partial
SAW17B57	*A. marmorata*	SAW	-13.968	171.862	2017/02/27	3,774,558	partial
SAW17B58	*A. marmorata*	SAW	-13.968	171.862	2017/02/27	6,366,828	partial
SOK16354	*A. marmorata*	SOK	-8.060	156.973	2016/04/28	5,215,368	no
SOL16V01	*A. marmorata*	SOL	-7.832	156.715	2016/04/28	3,542,070	no
SOL16V02	*A. marmorata*	SOL	-7.832	156.715	2016/04/28	4,502,312	no
SOL16V03	*A. marmorata*	SOL	-7.861	156.696	2016/10/31	7,042,480	no
SOL16V04	*A. marmorata*	SOL	-7.861	156.696	2016/10/31	7,799,996	no
SOL16V06	*A. marmorata*	SOL	-7.861	156.696	2016/10/31	3,521,598	no
SOL16V07	*A. marmorata*	SOL	-7.861	156.696	2016/10/31	4,174,210	no
SOL16V08	*A. marmorata*	SOL	-7.861	156.696	2016/10/31	7,173,740	no
SOL16V10	*A. marmorata*	SOL	-7.861	156.696	2016/10/31	998,684	no
SOL16V11	*A. marmorata*	SOL	-7.861	156.696	2016/10/31	681,170	no
SOL16V12	*A. marmorata*	SOL	-7.861	156.696	2016/10/31	6,531,962	no
SOL16V13	*A. marmorata*	SOL	-7.861	156.696	2016/10/31	8,609,022	no
SON16364	*A. marmorata*	SON	-8.804	158.203	2016/02/05	1,516,152	no
SON16370	*A. marmorata*	SON	-8.804	158.203	2016/02/05	4,083,426	no
SON16371	*A. marmorata*	SON	-8.804	158.203	2016/02/05	2,774,878	no
SON16375	*A. marmorata*	SON	-8.764	158.004	2016/02/05	1,279,182[3]	no
SON16376	*A. marmorata*	SON	-8.804	158.203	2016/02/05	1,854,150[3]	no
SOR16R01	*A. marmorata*	SOR	-8.055	156.582	2016/10/24	4,643,324	no
SOR16R02	*A. marmorata*	SOR	-8.055	156.582	2016/10/24	5,046,382	no
SOR16R03	*A. marmorata*	SOR	-8.055	156.582	2016/10/24	4,317,646	no
SOR16R06	*A. marmorata*	SOR	-8.055	156.582	2016/10/24	5,291,718	no
SOR16R07	*A. marmorata*	SOR	-8.055	156.582	2016/10/24	5,497,242	no
SOR16R09	*A. marmorata*	SOR	-8.084	156.600	2016/10/25	16,663,830	no
SOR16R12	*A. marmorata*	SOR	-8.084	156.600	2016/10/25	6,932,478	no
SOR16R13	*A. marmorata*	SOR	-8.036	156.536	2016/10/26	7,161,788	no
SOR16R20	*A. marmorata*	SOR	-8.036	156.536	2016/10/26	5,295,472	no
SOR16R21	*A. marmorata*	SOR	-8.036	156.536	2016/10/26	5,314,182	no
SOR16R22	*A. marmorata*	SOR	-8.036	156.536	2016/10/26	2,375,032	no
SOR16R23	*A. marmorata*	SOR	-8.036	156.536	2016/10/26	6,237,660	no
SOV16374	*A. megastoma*	SOV	-8.817	158.189	2016/05/03	4,844,384	no
SOV16377	*A. marmorata*	SOV	-8.764	158.004	2016/05/04	5,086,458	no
TAI15001	*A. marmorata*	TAI	24.716	121.835	2015/06/05	3,759,770	no
TAI15002	*A. marmorata*	TAI	24.716	121.835	2015/06/05	2,931,064	no
TAI15003	*A. marmorata*	TAI	24.716	121.835	2015/06/05	5,276,728	no
TAI15004	*A. marmorata*	TAI	24.716	121.835	2015/06/05	3,095,594	no
TAI15005	*A. marmorata*	TAI	24.716	121.835	2015/06/05	4,486,336	no
TAI15006	*A. marmorata*	TAI	24.716	121.835	2015/06/05	4,999,952	no
TAI15007	*A. marmorata*	TAI	24.716	121.835	2015/06/05	5,063,884	no
TAI15008	*A. marmorata*	TAI	24.716	121.835	2015/06/05	4,908,062	no
TAI15009	*A. marmorata*	TAI	24.716	121.835	2015/06/05	5,224,604	no
TAI15010	*A. marmorata*	TAI	24.716	121.835	2015/06/05	4,291,306	no
TAI15011	*A. marmorata*	TAI	24.716	121.835	2015/06/05	1,850,660	no
TAI15012	*A. marmorata*	TAI	24.716	121.835	2015/06/05	4,193,860	no
TAI15013	*A. marmorata*	TAI	24.716	121.835	2015/06/05	4,946,272	no

Supplementary Table 1 (continued)

Specimen ID	Species	Site	Lat.	Lon.	Date	# reads	Morphology
TAI15014	*A. marmorata*	TAI	24.716	121.835	2015/06/05	3,472,668	no
TAI15015	*A. marmorata*	TAI	24.716	121.835	2015/06/05	2,665,630	no
TAI15016	*A. marmorata*	TAI	24.716	121.835	2015/06/05	4,922,922	no
TAI15017	*A. marmorata*	TAI	24.716	121.835	2015/06/05	4,966,356	no
TAI15018	*A. marmorata*	TAI	24.716	121.835	2015/06/05	3,745,542	no
TAI15019	*A. marmorata*	TAI	24.716	121.835	2015/06/05	4,070,564	no
TAI15020	*A. marmorata*	TAI	24.716	121.835	2015/06/05	3,253,040	no
TAI15021	*A. marmorata*	TAI	24.716	121.835	2015/06/05	4,451,956	no
TAI15022	*A. marmorata*	TAI	24.716	121.835	2015/06/05	3,582,168	no
TAI15023	*A. marmorata*	TAI	24.716	121.835	2015/06/05	5,037,786	no
TAI15024	*A. marmorata*	TAI	24.716	121.835	2015/06/05	6,443,022	no
TAI15025	*A. marmorata*	TAI	24.716	121.835	2015/06/05	5,691,686	no
TAI15026	*A. marmorata*	TAI	24.716	121.835	2015/06/05	6,249,920	no
TAI15027	*A. marmorata*	TAI	24.716	121.835	2015/06/05	6,987,072	no
TAI15028	*A. marmorata*	TAI	24.716	121.835	2015/06/05	4,543,658	no
TAI15029	*A. marmorata*	TAI	24.716	121.835	2015/06/05	5,325,064	no
TAI15030	*A. marmorata*	TAI	24.716	121.835	2015/06/05	5,234,126	no
VAG12001	*A. marmorata*	VAG	-14.275	167.548	2012/01/17	2,943,378	yes
VAG12002	*A. marmorata*	VAG	-14.275	167.548	2012/01/18	1,740,398	yes
VAG12003	*A. marmorata*	VAG	-14.275	167.548	2012/01/19	1,406,994	yes
VAG12004	*A. megastoma*	VAG	-14.275	167.548	2012/01/21	6,051,382	yes
VAG12005	*A. megastoma*	VAG	-14.275	167.548	2012/01/21	6,977,268	yes
VAG12006	*A. megastoma*	VAG	-14.275	167.548	2012/01/21	2,449,900	yes
VAG12007	*A. megastoma*	VAG	-14.275	167.548	2012/01/21	3,009,804	yes
VAG12008	*A. megastoma*	VAG	-14.275	167.548	2012/01/21	2,672,694	yes
VAG12009	*A. megastoma*	VAG	-14.275	167.548	2012/01/21	132,204[1]	yes[2]
VAG12010	*A. megastoma*	VAG	-14.275	167.548	2012/01/21	1,859,440	yes
VAG12011	*A. megastoma*	VAG	-14.275	167.548	2012/01/21	2,859,824	yes
VAG12012	*A. marmorata*	VAG	-14.275	167.548	2012/01/21	885,610	yes
VAG12013	*A. megastoma*	VAG	-14.275	167.548	2012/01/21	4,839,628	yes
VAG12014	*A. megastoma*	VAG	-14.275	167.548	2012/01/22	3,150,160	yes
VAG12015	*A. megastoma*	VAG	-14.275	167.548	2012/01/22	2,285,658	yes
VAG12016	*A. marmorata*	VAG	-14.275	167.548	2012/01/22	2,609,394	yes
VAG12018	*A. marmorata*	VAG	-14.275	167.548	2012/01/22	2,795,482	yes
VAG12019	*A. marmorata*	VAG	-14.275	167.548	2012/01/22	3,403,952	yes
VAG12020	*A. megastoma*	VAG	-14.275	167.548	2012/01/22	8,602,088	yes
VAG12021	*A. megastoma*	VAG	-14.275	167.548	2012/01/22	254,536[1]	yes[2]
VAG12022	*A. marmorata*	VAG	-14.275	167.548	2012/01/22	1,420,164	yes
VAG12023	*A. interioris*	VAG	-14.275	167.548	2012/01/23	3,310,828	yes
VAG12024	*A. marmorata*	VAG	-14.275	167.548	2012/01/22	2,999,122	yes
VAG12025	*A. marmorata*	VAG	-14.275	167.548	2012/01/22	5,478,366	yes
VAG12026	*A. marmorata*	VAG	-14.275	167.548	2012/01/22	219,894[1]	yes[2]
VAG12027	*A. megastoma*	VAG	-14.275	167.548	2012/01/22	6,337,898	yes
VAG12028	*A. marmorata*	VAG	-14.275	167.548	2012/01/22	260,506[1]	yes[2]
VAG12029	*A. marmorata*	VAG	-14.275	167.548	2012/01/24	3,218,850	yes
VAG12030	*A. marmorata*	VAG	-14.275	167.548	2012/01/24	818,028	yes
VAG12031	*A. marmorata*	VAG	-14.275	167.548	2012/01/24	6,862,982	yes

Supplementary Table 1 (continued)

Specimen ID	Species	Site	Lat.	Lon.	Date	# reads	Morphology
VAG12032	*A. megastoma*	VAG	-14.275	167.548	2012/01/24	255,918[1]	yes[2]
VAG12033	*A. marmorata*	VAG	-14.275	167.548	2012/01/24	725,846	yes[2]
<u>VAG12034</u>	*A. megastoma*	VAG	-14.275	167.548	2012/01/24	8,292,228	yes
VAG12035	*A. marmorata*	VAG	-14.275	167.548	2012/01/24	528,696[1]	yes[2]
VAG12036	*A. marmorata*	VAG	-14.275	167.548	2012/01/24	11,598,732	yes
VAG12037	*A. marmorata*	VAG	-14.275	167.548	2012/01/24	2,505,822	yes[2]
VAG12038	*A. megastoma*	VAG	-14.275	167.548	2012/01/24	4,382,324	partial
VAG12039	*A. marmorata*	VAG	-14.261	167.605	2012/01/28	18,191,152	yes
VAG12040	*A. marmorata*	VAG	-14.261	167.605	2012/01/31	7,749,912	yes
<u>VAG12041</u>	*A. megastoma*	VAG	-14.261	167.605	2012/01/31	3,770,604	yes
VAG12044	*A. megastoma*	VAG	-14.261	167.605	2012/02/01	4,910,168	yes
VAG12045	*A. marmorata*	VAG	-14.261	167.605	2012/02/01	18,655,124	yes
VAG12046	*A. megastoma*	VAG	-14.261	167.605	2012/02/01	2,129,488	yes[2]
VAG12047	*A. megastoma*	VAG	-14.261	167.605	2012/02/01	1,262,984	yes[2]
VAG12049	*A. obscura*	VAG	-14.261	167.605	2012/01/31	5,359,114	yes
<u>VAG12050</u>	*A. obscura*	VAG	-14.261	167.605	2012/02/02	2,618,778	yes
VAG12051	*A. megastoma*	VAG	-14.261	167.605	2012/02/02	8,358,494	partial
VAG12052	*A. marmorata*	VAG	-14.261	167.605	2012/02/02	11,983,860	partial
VAG12053	*A. marmorata*	VAG	-14.261	167.605	2012/02/02	3,660,386	partial
VAG12054	*A. marmorata*	VAG	-14.261	167.605	2012/02/01	494,650[1]	yes[2]
VAG12055	*A. marmorata*	VAG	-14.261	167.605	2012/02/01	6,158,622	yes
<u>VAG12056</u>	*A. megastoma*	VAG	-14.261	167.605	2012/02/01	6,352,762	yes
VAG12059	*A. marmorata*	VAG	-14.261	167.605	2012/01/31	983,610	yes
VAG12060	*A. obscura*	VAG	-14.261	167.605	2012/02/01	1,435,834	yes
<u>VAG12061</u>	*A. obscura*	VAG	-14.261	167.605	2012/02/01	3,400,270	yes
<u>VAG12062</u>	*A. megastoma*	VAG	-14.261	167.605	2012/02/02	4,258,918	yes
<u>VAG12063</u>	*A. marmorata*	VAG	-14.261	167.605	2012/02/02	5,949,864	yes
<u>VAG12064</u>	*A. megastoma*	VAG	-14.261	167.605	2012/02/02	3,143,290	yes
<u>VAG12065</u>	*A. megastoma*	VAG	-14.261	167.605	2012/02/02	3,238,970	yes
<u>VAG12067</u>	*A. marmorata*	VAG	-14.261	167.605	2012/02/01	5,545,036	yes
<u>VAG12068</u>	*A. marmorata*	VAG	-14.261	167.605	2012/02/02	7,125,294	yes
<u>VAG13070</u>	*A. marmorata*	VAG	-14.261	167.605	2013/03/01	3,508,578	yes
VAG13071	*A. marmorata*	VAG	-14.261	167.605	2013/03/01	3,910,052	yes
VAG13072	*A. marmorata*	VAG	-14.261	167.605	2013/03/22	5,860,892	yes[2]
<u>VAG13073</u>	*A. obscura*	VAG	-14.261	167.605	2013/03/28	8,105,260	yes
<u>VAG13074</u>	*A. obscura*	VAG	-14.261	167.605	2013/03/28	7,979,904	yes
VAG13075	*A. megastoma*	VAG	-14.261	167.605	2013/03/27	4,794,394	yes
<u>VAG13076</u>	*A. megastoma*	VAG	-14.261	167.605	2013/03/30	3,882,404	yes
VAG13077	*A. marmorata*	VAG	-14.261	167.605	2013/03/27	6,948,582	yes
VAG13078	*A. marmorata*	VAG	-14.261	167.605	2013/03/26	6,147,476	yes
<u>VAG13079</u>	*A. marmorata*	VAG	-14.261	167.605	2013/03/26	7,965,414	yes
<u>VAG13080</u>	*A. obscura*	VAG	-14.261	167.605	2013/04/01	6,240,488	yes
<u>VAG13081</u>	*A. marmorata*	VAG	-14.261	167.605	2013/04/02	5,948,510	yes
<u>VAG13082</u>	*A. marmorata*	VAG	-14.261	167.605	2013/04/01	4,319,756	yes
VAG13083	*A. marmorata*	VAG	-14.261	167.605	2013/04/01	25,480[1]	yes[2]
<u>VAG13084</u>	*A. obscura*	VAG	-14.261	167.605	2013/04/01	2,637,702	yes
<u>VAG13085</u>	*A. marmorata*	VAG	-14.261	167.605	2013/04/02	6,013,544	yes

Supplementary Table 1 (continued)

Specimen ID	Species	Site	Lat.	Lon.	Date	# reads	Morphology
VAG13086	*A. megastoma*	VAG	-14.261	167.605	2013/04/03	3,298,274	yes
VAG13087	*A. marmorata*	VAG	-14.261	167.605	2013/04/03	1,115,480	partial

Supplementary Table 2: Per-species population-genetic parameters for tropical eel species. Reported parameters are calculated for the dataset for phylogenetic analyses (before reducing it to maximally five individuals per species). Heterozygosity (h), nucleotide diversity (π), and population mutation rate (Θ) were calculated twice; first assuming that all missing data are invariable, and second assuming that missing data mask genotypes that are equally variable as the observed ones. For *A. marmorata*, all parameters were calculated first for all individuals jointly and then for each of four populations: WIO, western Indian Ocean (sampling sites AFC, AFC, MAY, REU); SCS, South China Sea (sampling sites PHP, PHC, TAI); WSP, western South Pacific (sampling sites BOU, SO, VAG, NCA, SAA, SAW). n, Number of individuals used in genomic analyses.

Species	n	Completeness	# variable sites	h ($\times10^{-3}$)	π ($\times10^{-3}$)	Θ ($\times10^{-3}$)
A. marmorata	325	0.790	373,382	0.32/0.40	0.50/0.53	2.90/4.10
A. marmorata (WIO)	42	0.877	35,094	0.19/0.21	0.22/0.23	0.38/0.61
A. marmorata (Java)	21	0.599	25,187	0.14/0.24	0.26/0.28	0.32/0.82
A. marmorata (SCS)	63	0.877	201,746	0.53/0.61	0.69/0.70	2.04/2.65
A. marmorata (WSP)	199	0.765	191,935	0.30/0.39	0.44/0.46	1.60/3.23
A. luzonensis	20	0.737	113,858	0.72/0.98	1.12/1.19	1.47/2.53
A. bicolor	4	0.513	45,975	0.67/1.30	1.31/1.49	0.97/1.96
A. obscura	36	0.723	87,297	0.34/0.48	0.56/0.58	0.99/1.88
A. interioris	3	0.731	28,474	0.57/0.78	0.82/0.88	0.68/0.98
A. megastoma	41	0.738	127,411	0.43/0.58	0.71/0.71	1.40/2.41
A. mossambica	1	0.756	19,273	1.06/1.40	1.06/1.40	1.06/1.40

Supplementary Table 3: Per-species population-genetic parameters for tropical eel species. As Supplementary Table 2, but using the dataset for population-genetic analyses.

Species	n	Completeness	# variable sites	h ($\times10^{-3}$)	π ($\times10^{-3}$)	Θ ($\times10^{-3}$)
A. marmorata	325	0.910	146,998	0.65/0.72	0.86/0.88	1.14/1.20
A. marmorata (WIO)	42	0.948	46,793	0.53/0.56	0.57/0.57	0.51/0.67
A. marmorata (Java)	21	0.826	44,100	0.49/0.59	0.60/0.60	0.56/0.89
A. marmorata (SCS)	63	0.940	68,595	0.70/0.75	0.83/0.83	0.69/0.91
A. marmorata (WSP)	199	0.901	139,822	0.68/0.76	0.81/0.81	1.17/1.28
A. luzonensis	20	0.859	53,935	0.62/0.72	0.80/0.84	0.69/0.99
A. bicolor	4	0.721	13,436	0.23/0.31	0.34/0.37	0.28/0.43
A. obscura	36	0.844	60,983	0.40/0.48	0.48/0.54	0.69/0.98
A. interioris	3	0.877	12,487	0.27/0.30	0.32/0.34	0.30/0.35
A. megastoma	41	0.818	72,415	0.56/0.69	0.74/0.80	0.80/1.18
A. mossambica	1	0.777	2,595	0.14/0.18	0.14/0.18	0.14/0.18

Supplementary Table 4: Mean pairwise genetic distance between tropical eel species. Genetic distances were calculated as uncorrected p-distances (the proportion of sites at which two sequences are different), based on a concatenated alignment of 20,637 RAD loci. These loci were selected to have no fully missing sequences for the maximally five individuals per species with the overall lowest proportion of missing data; only these individuals are included in the alignment. As p-distances are symmetric, species 1 and 2 are exchangeable. Rows are sorted by p-distance.

Species 1	Species 2	# pairs	Mean p-distance
A. luzonensis	*A. marmorata*	25	0.0053
A. interioris	*A. luzonensis*	15	0.0060
A. bicolor	*A. obscura*	20	0.0064
A. interioris	*A. marmorata*	15	0.0065
A. bicolor	*A. interioris*	12	0.0067
A. interioris	*A. obscura*	15	0.0067
A. luzonensis	*A. obscura*	25	0.0072
A. bicolor	*A. luzonensis*	20	0.0073
A. bicolor	*A. marmorata*	20	0.0076
A. marmorata	*A. obscura*	25	0.0076
A. interioris	*A. megastoma*	15	0.0079
A. luzonensis	*A. megastoma*	25	0.0081
A. marmorata	*A. megastoma*	25	0.0082
A. megastoma	*A. obscura*	25	0.0088
A. bicolor	*A. megastoma*	20	0.0090
A. megastoma	*A. mossambica*	5	0.0103
A. interioris	*A. mossambica*	3	0.0105
A. luzonensis	*A. mossambica*	5	0.0106
A. marmorata	*A. mossambica*	5	0.0107
A. mossambica	*A. obscura*	5	0.0113
A. bicolor	*A. mossambica*	4	0.0116

Supplementary Table 5: Genome assemblies. The results of the BUSCO analysis are given in the order complete (c), complete and single copy (c+s), complete and duplicated (c+d), fragmented (f), missing (m). A total of 2,586 BUSCOs were searched for each assembly. scf., scaffold; ctg., contig.

Species	Assembly version	Assembly size	N50 scf.	N50 ctg.	—BUSCOs (c/c+s/c+d/f/m)—				
A. marmorata	Uncorrected	880,647,635	64,770	15,086	2,217	2,028	189	254	115
	Pilon-corrected	882,006,954	64,942	16,529	2,215	2,020	195	254	117
A. megastoma	Uncorrected	877,063,880	61,871	13,547	2,203	2,013	190	264	119
	Pilon-corrected	877,765,645	61,910	14,572	2,207	2,015	192	260	119
A. obscura	Uncorrected	881,549,187	54,844	12,017	2,161	1,977	184	307	118
	Pilon-corrected	882,390,062	54,849	12,681	2,168	1,982	186	304	114

Supplementary Table 6: Cross-validation (CV) error values of maximum-likelihood ancestry inference with ADMIXTURE.
CV errors were inferred for the models $K = 1$ to $K = 8$ based on 117,638 variable sites for five replicates (R1 to R5) per model.

Model	R1	R2	R3	R4	R5
$K = 1$	0.49028	0.49037	0.49046	0.49041	0.49040
$K = 2$	0.29901	0.29916	0.29903	0.29902	0.29913
$K = 3$	0.22883	0.22879	0.22882	0.26443	0.22880
$K = 4$	0.24704	0.19584	0.19579	0.19590	0.24694
$K = 5$	0.17819	0.17819	0.17816	0.17822	0.17820
$K = 6$	0.17491	0.17496	0.17500	0.17500	0.17488
$K = 7$	0.17614	0.17613	0.17610	0.17614	0.17380
$K = 8$	0.17508	0.17421	0.17849	0.17435	0.17487

Supplementary Table 7 (next page): Hybrid individuals.
Genomic and morphological characteristics of hybrid individuals identified via ancestry painting. Note that for backcrossed hybrids, the identity of maternal and paternal species are ambiguous because either the mother or the father is a hybrid itself; in this case, the name listed as maternal species indicates the species identity of the mitochondrial genome. h_{fixed}, heterozygosity at sites fixed between parental species; $f_{\text{m,genome}}$, proportion of genome derived from the maternal species; $f_{\text{m,morphology}}$, similarity to morphology of maternal species, relative to morphology of paternal species.

Specimen ID	Maternal species	Paternal species	Date	h_{fixed}	$f_{\text{m,genome}}$	$f_{\text{m,morphology}}$	Interpretation
BOU15031	*A. marmorata*	*A. megastoma*	04/2015	0.479	0.250	0.257	Backcross[1]
SAA16011	*A. marmorata*	*A. megastoma*	08/2016	0.974	0.506	NA	F1
SAA16012	*A. marmorata*	*A. megastoma*	08/2016	0.966	0.500	NA	F1
SAA16013	*A. marmorata*	*A. megastoma*	08/2016	0.981	0.510	NA	F1
SAA16024	*A. marmorata*	*A. megastoma*	08/2016	0.550	0.716	NA	Backcross[2]
SAA16027	*A. marmorata*	*A. megastoma*	08/2016	0.956	0.500	NA	F1
SAW17B27	*A. marmorata*	*A. megastoma*	02/2017	0.972	0.506	NA	F1
SAW17B49	*A. marmorata*	*A. megastoma*	03/2017	0.969	0.512	NA	F1
VAG12012	*A. marmorata*	*A. megastoma*	01/2012	0.971	0.515	0.524	F1
VAG12018	*A. marmorata*	*A. megastoma*	01/2012	0.977	0.500	0.685	F1
VAG12019	*A. marmorata*	*A. megastoma*	01/2012	0.975	0.508	0.572	F1
VAG12024	*A. marmorata*	*A. megastoma*	01/2012	0.510	0.263	0.504	Backcross[1]
VAG12029	*A. marmorata*	*A. megastoma*	01/2012	0.459	0.767	0.746	Backcross[2]
VAG12037	*A. marmorata*	*A. megastoma*	01/2012	0.817	0.575	NA	F1
VAG12044	*A. megastoma*	*A. marmorata*	02/2012	0.973	0.514	0.537	F1
VAG12053	*A. marmorata*	*A. megastoma*	02/2012	0.542	0.705	NA	Backcross[2]
VAG12055	*A. marmorata*	*A. megastoma*	02/2012	0.460	0.250	0.545	Backcross[1]
VAG13071	*A. marmorata*	*A. megastoma*	03/2013	0.954	0.512	0.580	F1
VAG13078	*A. marmorata*	*A. megastoma*	03/2013	0.972	0.500	0.470	F1
VAG13087	*A. marmorata*	*A. megastoma*	04/2013	0.584	0.324	NA	Backcross[1,3]
VAG12040	*A. marmorata*	*A. obscura*	02/2012	0.951	0.507	0.540	F1
VAG12045	*A. marmorata*	*A. obscura*	02/2012	0.899	0.510	0.618	F1
VAG13077	*A. marmorata*	*A. obscura*	03/2013	0.957	0.500	0.537	F1
VAG12049	*A. obscura*	*A. megastoma*	01/2012	0.977	0.506	0.512	F1
BOU15017	*A. marmorata*	*A. interioris*	04/2015	0.939	0.520	0.666	F1
210 *A. marmorata*, *A. megastoma*, and *A. obscura*				0.040	0.977		Unadmixed[4]

[1]The mitochondrial genome of *A. marmorata* and the $f_{\text{m,genome}}$ around 0.25 indicate that the mother of the mother of this individual was an *A. marmorata* but all other grantparents were *A. megastoma*.

[2]The mitochondrial genome of *A. marmorata* and the $f_{\text{m,genome}}$ around 0.75 indicate that one of the grandparents of this individual, but not the mother of the mother, was an *A. megastoma* and all other grandparents were *A. marmorata*.

[3]The $f_{\text{m,genome}}$ between 0.25 and 0.5 could indicate that one of the grandparents of VAG13087 was itself admixed. However, as VAG13087 has a comparatively large proportion of missing data and its genotypes are available for only 125 of 302 fixed sites, we do not consider this evidence of earlier admixture reliable enough to warrant further discussion.

[4]For comparison, we calculated h_{fixed} and $f_{\text{m,genome}}$ for all *A. marmorata*, *A. megastoma*, and *A. obscura* individuals except "core" individuals (because these had been used to identify fixed sites), the identified hybrids listed above, and individuals with less than 2 million reads. After excluding these, 210 individuals remained for the analysis, of which 194 were mitochondrially assigned to *A. marmorata*, 12 to *A. megastoma*, and 4 to *A. obscura*. Given here are the maximum value for h_{fixed} and the minimum value for $f_{\text{m,genome}}$ across the 210 individuals; mean values are $h_{\text{fixed}}= 0.005$ and $f_{\text{m,genome}}= 0.996$. Thus, no individuals have a $f_{\text{m,genome}}$ between 0.767 and 0.977.

Supplementary Table 8: Frequency of hybrids at sampling sites.
n_t, number of sampled specimens; n_g, number of individuals used in genomic analyses, excluding those with low sequence quality.

Site	Location	Country	n_t	n_g	# hybrids	Frequency (%)
AFC	Eastern Cape	South Africa	15	14	0	0
AFS	Lubombo	Swaziland	1	1	0	0
MAY	Mayotte	France	18	18	0	0
REU	Réunion	France	10	10	0	0
JAV	Java	Indonesia	30	27	0	0
PHP	Pagadian	Philippines	27	27	0	0
PHC	Cagayan	Philippines	31	26	0	0
TAI	Yilan County	Taiwan	30	30	0	0
BOU	Bougainville	Papua New Guinea	30	30	2	6.7
SOK	Kolombangara	Solomon Islands	1	1	0	0
SOL	Vella Lavella	Solomon Islands	11	11	0	0
SON	Nggatokae	Solomon Islands	5	3	0	0
SOR	Ranongga	Solomon Islands	12	12	0	0
SOV	Vangunu	Solomon Islands	2	2	0	0
VAG	Gaua	Vanuatu	79	71	16	22.5
NCA	New Caledonia	France	45	45	0	0
SAW	Upolu	Samoa	71	67	2	3.0
SAA	Tutuila	American Samoa	38	35	5	14.3
Total			456	430	25	5.8

Supplementary Table 9: Introgression statistics for species quartets.
All species quartets compatible with the inferred species tree were tested. Quartet comparisons are sorted by D values. mar, *A. marmorata*; luz, *A. luzonensis*; int, *A. interioris*; obs, *A. obscura*; bic, *A. bicolor*; meg, *A. megastoma*; mos, *A. mossambica*; ang, *A. anguilla*; n, number of informative sites; C_{BBAA}, number of "BBAA" sites; C_{ABBA}, number of "ABBA" sites; C_{BABA}, number of "BABA" sites; D, Patterson's D statistic [46, 47]; f_4, the f_4 statistic [48]; p, p-value for $f_4 = 0$ assessed through simulations with the F4 program [49]. p-values are based on one-sided comparisons and not adjusted for multiple comparisons. The comparison reported in the last table row is based on WGS reads of a single individual of *A. obscura*, *A. marmorata*, and *A. megastoma*, aligned to the available reference genome assembly of *A. anguilla* [28].

P1	P2	P3	Outgroup	n	C_{BBAA}	C_{ABBA}	C_{BABA}	D	f_4	p
mar	luz	int	mos	10,290	273.1	182.7	77.1	0.406	-0.0070	0.000
mar	luz	int	meg	13,396	335.9	207.6	98.2	0.358	-0.0054	0.000
mar	luz	obs	meg	15,689	412.1	186.6	93.0	0.334	-0.0043	0.000
mar	luz	obs	mos	12,054	358.2	162.7	83.7	0.321	-0.0048	0.000
mar	bic	int	mos	7,772	100.9	266.3	138.4	0.316	-0.0109	0.000
mar	luz	bic	mos	11,542	311.9	158.1	82.8	0.313	-0.0052	0.000
mar	bic	int	meg	9,680	136.6	295.3	168.6	0.273	-0.0077	0.000
mar	luz	bic	meg	14,793	360.6	168.2	103.4	0.239	-0.0035	0.000
mar	obs	int	meg	10,208	137.3	307.9	197.8	0.218	-0.0051	0.000
mar	obs	int	mos	8,068	106.3	268.6	173.1	0.216	-0.0086	0.000
obs	bic	mar	meg	11,372	653.6	104.7	71.2	0.191	-0.0025	0.002
obs	bic	int	mos	8,304	373.7	123.8	84.1	0.191	-0.0030	0.005
obs	bic	int	meg	10,444	471.0	125.7	86.6	0.184	-0.0026	0.003
obs	bic	mar	mos	9,068	555.9	98.8	68.3	0.182	-0.0016	0.078
obs	bic	luz	mos	12,557	487.2	113.4	80.0	0.173	-0.0022	0.008
mar	int	meg	mos	9,951	482.4	96.4	72.7	0.140	-0.0023	0.026
obs	bic	luz	meg	16,064	582.5	108.7	82.5	0.137	-0.0015	0.017
mar	luz	meg	mos	13,129	677.0	69.0	52.9	0.133	-0.0008	0.201
luz	mar	bic	int	14,675	392.7	105.4	84.5	0.110	-0.0011	0.106
luz	bic	int	meg	14,246	133.6	228.4	191.0	0.089	-0.0015	0.062
luz	int	meg	mos	13,632	550.4	82.4	70.2	0.080	-0.0007	0.192
luz	bic	int	mos	11,133	111.4	197.5	168.3	0.080	-0.0022	0.042
mar	bic	meg	mos	11,134	441.3	110.9	95.0	0.077	-0.0003	0.430
luz	mar	obs	int	15,500	417.8	111.7	96.5	0.073	-0.0003	0.406
mar	obs	meg	mos	11,647	458.9	126.1	110.0	0.068	-0.0009	0.241
bic	obs	mar	int	11,303	520.4	80.0	73.0	0.046	-0.0007	0.261
obs	bic	meg	mos	11,761	813.0	64.7	59.5	0.042	-0.0002	0.447
bic	obs	luz	int	15,856	526.5	78.1	72.1	0.040	-0.0010	0.141
obs	int	meg	mos	11,017	557.5	96.2	90.8	0.029	-0.0011	0.137
luz	bic	meg	mos	14,602	480.2	97.0	93.1	0.020	0.0002	0.416
luz	obs	int	meg	15,143	144.2	227.1	221.7	0.012	0.0005	0.300
bic	int	meg	mos	10,451	535.3	84.0	82.0	0.012	-0.0007	0.213
luz	obs	meg	mos	15,405	507.8	107.9	106.2	0.008	-0.0001	0.461
luz	obs	int	mos	11,638	107.7	197.6	198.7	-0.003	-0.0007	0.303
obs	luz	meg	mos	15,405	507.8	106.2	107.9	-0.008	0.0001	0.463
int	bic	meg	mos	10,451	535.3	82.0	84.0	-0.012	0.0007	0.227

Supplementary Table 9 (continued)

P1	P2	P3	Outgroup	n	C_{BBAA}	C_{ABBA}	C_{BABA}	D	f_4	p
bic	luz	meg	mos	14,602	480.2	93.1	97.0	-0.020	-0.0002	0.420
int	obs	meg	mos	11,017	557.5	90.8	96.2	-0.029	0.0011	0.164
obs	bic	luz	int	15,856	526.5	72.1	78.1	-0.040	0.0010	0.141
bic	obs	meg	mos	11,761	813.0	59.5	64.7	-0.042	0.0002	0.420
obs	bic	mar	int	11,303	520.4	73.0	80.0	-0.046	0.0007	0.263
obs	mar	meg	mos	11,647	458.9	110.0	126.1	-0.068	0.0009	0.232
mar	luz	obs	int	15,500	417.8	96.5	111.7	-0.073	0.0003	0.384
bic	mar	meg	mos	11,134	441.3	95.0	110.9	-0.077	0.0003	0.403
int	luz	meg	mos	13,632	550.4	70.2	82.4	-0.080	0.0007	0.193
mar	luz	bic	int	14,675	392.7	84.5	105.4	-0.110	0.0011	0.119
luz	mar	meg	mos	14,059	690.1	53.8	69.0	-0.124	0.0008	0.183
bic	obs	luz	meg	16,064	582.5	82.5	108.7	-0.137	0.0015	0.013
int	mar	meg	mos	9,951	482.4	72.7	96.4	-0.140	0.0023	0.025
bic	obs	luz	mos	12,557	487.2	80.0	113.4	-0.173	0.0022	0.009
bic	obs	mar	mos	9,068	555.9	68.3	98.8	-0.182	0.0016	0.074
bic	obs	int	meg	10,444	471.0	86.6	125.7	-0.184	0.0026	0.006
bic	obs	mar	meg	11,372	653.6	71.2	104.7	-0.191	0.0025	0.002
bic	obs	int	mos	8,304	373.7	84.1	123.8	-0.191	0.0030	0.006
luz	mar	bic	meg	14,793	360.6	103.4	168.2	-0.239	0.0035	0.000
luz	mar	bic	mos	11,542	311.9	82.8	158.1	-0.313	0.0052	0.000
luz	mar	obs	mos	12,054	358.2	83.7	162.7	-0.321	0.0048	0.000
luz	mar	obs	meg	15,689	412.1	93.0	186.6	-0.334	0.0043	0.000
luz	mar	int	meg	13,396	335.9	98.2	207.6	-0.358	0.0054	0.000
luz	mar	int	mos	10,290	273.1	77.1	182.7	-0.406	0.0070	0.000
obs	mar	meg	ang	23,165,451	1638567.0	596786.0	587910.0	0.007	—	—

Supplementary Table 10: Introgression statistics for quartets of species and *A. marmorata* populations.
As Supplementary Table 9, but for comparisons involving individual *A. marmorata* populations. Only a single quartet involving *A. marmorata* appeared significant in Supplementary Table 9 and was tested further with separate populations. In addition, the possibility of different degrees of introgression between *A. luzonensis* and the four *A. marmorata* populations was explored because *A. luzonensis* appeared to share more coancestry with the South China Sea population of *A. marmorata* than with other populations in the fineRADstructure analysis (Supplementary Figure 8). O, Outgroup; WIO, western Indian Ocean (sampling sites AFC, AFC, MAY, REU); SCS, South China Sea (sampling sites PHP, PHC, TAI); WSP, western South Pacific (sampling sites BOU, SO, VAG, NCA, SAA, SAW).

P1	P2	P3	O	n	C_{BBAA}	C_{ABBA}	C_{BABA}	D	f_4	p
obs	bic	mar (WIO)	meg	11,104	653.0	105.5	71.5	0.192	-0.0016	0.042
obs	bic	mar (WIO)	mos	8,869	555.0	98.8	68.8	0.179	-0.0016	0.099
obs	bic	mar (Java)	meg	13,543	681.5	103.2	70.2	0.191	-0.0031	0.000
obs	bic	mar (Java)	mos	8,297	508.0	89.2	62.1	0.179	-0.0025	0.008
obs	bic	mar (SCS)	meg	10,794	632.5	97.2	68.1	0.176	-0.0029	0.000
obs	bic	mar (SCS)	mos	13,340	557.4	98.6	68.7	0.179	-0.0014	0.079
obs	bic	mar (WSP)	meg	16,834	656.1	100.0	70.8	0.171	-0.0015	0.025
obs	bic	mar (WSP)	mos	11,181	559.9	99.8	68.6	0.186	-0.0020	0.048
mar (WIO)	mar (Java)	luz	meg	12,097	864.9	23.7	28.0	-0.083	0.0002	0.319
mar (WIO)	mar (Java)	luz	mos	9,190	627.4	21.8	24.7	-0.063	0.0004	0.350
mar (WIO)	mar (SCS)	luz	meg	18,097	735.4	48.7	63.9	-0.135	0.0006	0.256
mar (WIO)	mar (SCS)	luz	mos	13,755	538.9	43.0	49.9	-0.074	0.0005	0.329
mar (WIO)	mar (WSP)	luz	meg	15,210	842.0	27.2	78.1	-0.483	0.0033	0.025
mar (WIO)	mar (WSP)	luz	mos	12,015	612.6	26.2	41.9	-0.232	0.0012	0.238
mar (WSP)	mar (WIO)	luz	meg	15,210	842.0	78.1	27.2	0.483	-0.0033	0.023[1]
mar (WSP)	mar (WIO)	luz	mos	12,015	612.6	41.9	26.2	0.232	-0.0012	0.218
mar (WSP)	mar (Java)	luz	meg	15,119	831.1	76.9	30.4	0.434	-0.0034	0.021[1]
mar (WSP)	mar (Java)	luz	mos	11,976	605.8	41.6	28.7	0.183	-0.0010	0.253
mar (WSP)	mar (SCS)	luz	meg	20,938	704.2	101.3	65.7	0.213	-0.0017	0.098
mar (WSP)	mar (SCS)	luz	mos	16,383	517.6	62.6	53.7	0.076	-0.0003	0.361

[1]Note that the support for introgression between *A. luzonensis* and the *A. marmorata* populations from the western Indian Ocean and Java is not robust to outgroup choice and no longer significant after correcting for multiple tests.

Supplementary Table 11: Genomic rearrangements supported by whole-genome alignment. Evidence for possible genomic rearrangements from pairwise whole-genome alignment between the *A. anguilla* reference genome assembly [28] and either the *A. japonica* (jap) genome assembly [21] or one of the newly generated genome assemblies for *A. marmorata* (mar), *A. megastoma* (meg), and *A. obscura* (obs). Scaffold IDs and regions refer to the *A. anguilla* reference genome assembly. Possible inversions ("i") and transpositions ("t") were recorded when sequences in multiple alignment blocks between the *A. anguilla* assembly and another assembly showed different orientations or orders, respectively (see Supplementary Notes 5-6 for details). When a single alignment block or multiple closely spaced alignment blocks (with sequences in identical orientation and order) for the same two scaffolds spanned the whole region, the absence ("n") of a rearrangement was recorded. A question mark indicates insufficient support for inversions, transpositions, or their absence. Lower-case records ("i", "t", and "n") indicate support obtained with an automatic rearrangement identification pipeline while upper-case records in bold ("**I**", "**T**", and "**N**") indicate additional support from visual inspection of dot plots comparing pairs of scaffolds. The distance to the nearest coding sequence in the *A. anguilla* assembly, according to the gene prediction with AUGUSTUS [32] (see Supplementary Note 7), is given; a distance of 0 indicates that the region overlaps with a coding sequence. In cases where these distances are shorter than 2,000 bp, we used BLASTP [25] searches to compare the translated coding sequences to the zebrafish (*Danio rerio*) proteome (assembly version GRCz11; NCBI accession GCA_000002035.4 [33]) to identify homologous proteins. Protein IDs, *e*-values, and percentages of identical amino acids (p_{ident}) are reported for the best match in each BLASTP search. Underlined sets of records indicate genomic rearrangements that are supported by both the identification pipeline and the visual inspection as species-specific, affecting only one of *A. marmorata*, *A. megastoma*, and *A. obscura*. These nine rearrangements with comparatively strongest support are visualized in Supplementary Figure 21 (jointly in the case of two rearrangements identified on scaffold scf0058).

Scaffold	Region (bp)	jap	mar	meg	obs	Dist. (bp)	Protein	e	p_{ident}
scf0002	2,115,902-2,116,902	**T**	**N**	**N**	**N**	1,329	ENSDARP00000012730	0.0	82
scf0012	52,975-53,974	i	**N**	?	?	3,875			
scf0012	55,073-64,200	i	?	?	?	0	ENSDARP00000002544	4E-20	41
scf0012	610,945-611,945	t	**N**	**N**	**N**	16,724			
scf0023	1,800,176-1,801,175	**T**	**N**	**N**	**N**	5,910			
scf0058	994,382-995,382	**N**	**N**	**N**	**I**	20,577			
scf0058	995,807-996,807	**N**	**N**	**N**	**I**	19,152			
scf0102	118,834-123,017	?	?	?	t	19,301			
scf0105	965,601-966,600	i	**I**	**I**	?	7,317			
scf0105	967,002-968,002	**I**	**I**	**I**	?	8,718			
scf0136	2,243,490-2,245,130	?	**T**	?	?	9,048			
scf0136	2,415,454-2,416,454	i	?	**I**	**I**	12,056			
scf0136	2,416,795-2,417,794	i	i	i	i	13,397			
scf0156	327,153-328,153	i	i	?	?	10,658			
scf0156	328,541-329,540	**I**	**I**	i	i	9,271			
scf0160	3,342,645-3,344,015	?	?	t	?	1,619	ENSDARP00000097499	0.0	63

Supplementary Table 11 (continued):

Scaffold	Region (bp)	jap	mar	meg	obs	Dist. (bp)	Protein	e	p_{ident}
scf0195	1,126,521-1,127,520	?	?	**N**	i	50,042			
scf0195	1,596,002-1,597,002	**N**	t	**N**	**N**	7,381			
scf0248	83,204-89,355	?	?	i	?	10,593			
scf0306	298,096-299,176	?	?	**I**	**N**	9,839			
scf0315	413,254-416,766	?	t	?	?	3,901			
scf0329	2,840,974-2,841,973	?	**N**	i	n	3,895			
scf0329	3,664,279-3,666,236	i	?	i	?	3,711			
scf0357	5,042-6,194	?	?	?	t	16,353			
scf0389	576,893-579,277	?	?	?	**I**	21,034			
scf0392	167,788-170,892	?	?	i	?	0	ENSDARP00000062165	6E-37	39
scf0392	177,678-178,790	?	?	i	?	1,778	ENSDARP00000130141	1E-70	53
scf0402	967,259-968,724	i	n	n	n	0	ENSDARP00000090766	0.0	83
scf0402	961,570-962,569	?	**I**	?	i	0	ENSDARP00000090766	2E-160	67
scf0419	49,504-50,888	t	t	t	t	14,249			
scf0445	74,276-81,244	?	?	?	i	37,248			
scf0445	229,239-230,238	?	?	?	i	5,473			
scf0447	17,978-18,977	?	**I**	?	**N**	68,702			
scf0448	655,524-657,133	**N**	**N**	?	t	20,456			
scf0463	69,108-72,730	?	i	?	?	717	ENSDARP00000086268	8E-74	53
scf0464	1,113,109-1,114,108	t	?	?	t	22,419			
scf0464	3,061,885-3,062,884	**N**	**N**	**N**	t	48,705			
scf0478	515,658-516,658	**T**	**N**	**N**	**N**	22,350			
scf0499	62,645-64,928	t	?	?	?	38,428			
scf0523	345,327-354,200	t	?	t	?	0	ENSDARP00000129600	5E-07	24
scf0525	128,297-132,890	?	i	**N**	**N**	946	ENSDARP00000029601	7E-59	71
scf0526	846,581-848,016	t	?	?	?	17,037			
scf0531	273,605-274,604	n	i	**N**	**N**	27,436			
scf0542	880,074-885,340	?	t	?	?	431	ENSDARP00000063057	0.0	61
scf0542	928,608-929,608	**N**	?	t	?	4,040			
scf0544	267,780-268,964	?	**N**	**N**	t	0	ENSDARP00000021675	9E-123	61
scf0558	33,663-34,662	n	?	i	i	2,198			
scf0563	1,905,051-1,906,050	**T**	**N**	**N**	**N**	302	ENSDARP00000149410	7E-92	64
scf0571	151,830-152,830	?	?	t	?	43,500			
scf0576	3,140,417-3,146,666	t	?	?	?	32,299			
scf0584	258,668-263,954	?	?	i	?	10,287			
scf0584	269,175-270,174	t	**N**	**N**	?	4,067			
scf0596	325,397-326,397	t	**N**	?	**N**	95	ENSDARP00000126885	3E-58	36
scf0598	316,128-318,639	?	?	?	t	2,040			
scf0599	80,572-81,917	**I**	i	i	**I**	6,681			
scf0599	82,659-83,658	**I**	i	**I**	**I**	7,455			
scf0603	33,631-34,631	**I**	**I**	**I**	**I**	47,457			
scf0603	34,734-35,734	**I**	**I**	**I**	**I**	46,354			
scf0603	647,785-648,785	i	**N**	**N**	**N**	0	ENSDARP00000136997	0.0	77
scf0606	2,735,360-2,736,360	?	**N**	?	i	3,637			
scf0621	166,715-167,715	**N**	t	**N**	**N**	5,822			
scf0645	471,078-472,078	?	**I**	?	?	24,475			
scf0645	472,836-473,944	?	**I**	?	**I**	26,233			

Supplementary Table 11 (continued):

Scaffold	Region (bp)	jap	mar	meg	obs	Dist. (bp)	Protein	e	p_{ident}
scf0645	954,171-955,171	?	i	**N**	?	4,838			
scf0661	1,957,158-1,959,274	i	i	i	i	6,965			
scf0661	1,961,191-1,962,875	i	i	i	i	10,998			
scf0685	1,005,750-1,006,750	**N**	**N**	**N**	t	12,002			
scf0709	281,497-282,496	t	?	**N**	**N**	690	ENSDARP00000124540	6E-15	35
scf0709	1,128,814-1,129,814	?	?	**N**	i	36,694			
scf0709	1,131,044-1,133,400	?	?	**N**	**I**	38,924			
scf0725	855,352-856,352	**N**	**N**	t	**N**	24,330			
scf0728	5,009-6,008	?	?	?	**T**	462	ENSDARP00000055920	4E-09	30
scf0728	376,488-377,487	?	**N**	?	t	2,984			
scf0740	541,739-542,739	**I**	**N**	**N**	**N**	51,938			
scf0740	543,414-544,414	i	**N**	**N**	**N**	53,613			
scf0775	42,721-43,720	**N**	**T**	?	?	436	ENSDARP00000064152	0.0	60
scf0799	55,095-56,095	**N**	t	?	?	3,469			
scf0810	535,438-536,437	i	i	i	i	3,084			
scf0810	540,436-541,752	i	?	?	?	2,922			
scf0839	489,992-492,139	?	i	?	?	0	ENSDARP00000021860	0.0	78
scf0842	631,075-632,075	**N**	**N**	**N**	**T**	1,126	ENSDARP00000114637	0.0	64
scf0844	251,592-254,960	?	?	?	i	2,747			
scf0850	84,713-85,713	**I**	**I**	**I**	**I**	62,609			
scf0850	86,265-87,265	**I**	**I**	**I**	**I**	64,161			
scf0869	40,033-44,164	?	?	t	?	79,459			
scf0873	373,044-374,075	i	**N**	**N**	?	3,351			
scf0891	1,949,018-1,950,018	?	i	**N**	?	20,499			
scf0895	315,630-316,695	i	?	?	?	0	ENSDARP00000071423	7E-171	70
scf0895	309,124-310,124	n	n	i	n	4,254			
scf0915	32,539-33,863	?	**I**	?	?	26,052			
scf0933	31,347-32,347	?	**N**	?	i	29,437			
scf0936	164,667-167,398	?	t	t	t	11,564			
scf0951	113,224-114,224	**N**	**N**	?	t	1,770			
scf0953	1,177,157-1,179,535	?	?	**N**	t	8,414			
scf0991	156,975-160,867	?	?	?	t	679	ENSDARP00000110758	1E-22	32
scf1010	21,681-22,681	?	i	**N**	?	198	ENSDARP00000137800	3E-107	90
scf1016	795,631-796,836	t	**N**	**N**	**N**	253,015			
scf1039	516,803-517,803	i	?	?	?	21,050			
scf1062	1,106,630-1,107,630	?	**N**	**N**	**T**	25,247			
scf1062	1,116,550-1,117,550	t	**N**	**N**	**N**	15,327			
scf1064	168,384-170,234	**I**	**I**	**I**	**I**	9,859			
scf1064	171,825-177,719	**I**	**I**	**I**	**I**	5,391			
scf1091	176,598-177,598	**N**	**I**	**N**	**N**	2,899			
scf1117	113,731-114,730	n	?	?	**I**	2,328			
scf1151	5,974-6,973	**N**	t	**N**	**N**	0	ENSDARP00000045908	2E-28	43
scf1183	42,092-43,092	**N**	**N**	**I**	**N**	3,102			
scf1185	59,573-60,956	?	i	i	?	678	No match	NA	NA
scf1186	396,470-398,388	?	?	i	?	0	ENSDARP00000155576	0.002	31
scf1201	157,099-158,099	**N**	**N**	n	**N**	24,168			
scf1207	89,147-90,146	n	**N**	**I**	**N**	261	ENSDARP00000131892	2E-57	66

Supplementary Table 11 (continued):

Scaffold	Region (bp)	jap	mar	meg	obs	Dist. (bp)	Protein	e	p_{ident}
scf1210	92,067-96,819	?	?	**I**	?	0	ENSDARP00000132236	9E-65	83
scf1227	145,858-152,706	?	i	?	?	11,238			
scf1244	513,816-514,815	?	?	t	t	22,149			
scf1253	253,467-256,396	i	**N**	**N**	**N**	0	ENSDARP00000128891	0.0	78
scf1272	128,039-134,102	?	?	i	?	85,121			
scf1292	5,811,513-5,812,513	**N**	i	**N**	**N**	28,423			
scf1307	1,227,169-1,228,786	?	t	?	?	15,229			
scf1326	44,366-45,365	**I**	**I**	**I**	**I**	12,721			
scf1326	46,393-47,627	**I**	**I**	?	**I**	14,748			
scf1329	556,939-561,398	?	?	t	?	20,672			
scf1362	177,499-178,499	t	**N**	**N**	**N**	5,061			
scf1399	13,212-15,371	?	t	?	t	484	ENSDARP00000059310	8E-106	54
scf1406	445,350-446,589	?	?	?	i	32,987			
scf1463	601,381-602,381	?	t	**N**	**N**	60,458			
scf1486	2,670,131-2,671,130	t	**N**	**N**	**N**	7,480			
scf1486	2,796,525-2,798,196	?	?	?	t	0	ENSDARP00000101608	9E-22	35
scf1526	1,441,401-1,449,572	?	?	?	t	0	ENSDARP00000054593	9E-62	44
scf1536	67,234-68,233	**T**	**T**	**T**	t	9,605			
scf1538	154,298-155,298	?	t	**N**	**N**	1,837	ENSDARP00000026978	2E-72	83
<u>scf1569</u>	<u>75,661-76,661</u>	<u>**N**</u>	<u>**T**</u>	<u>**N**</u>	<u>**N**</u>	<u>1,820</u>	<u>No match</u>	<u>NA</u>	<u>NA</u>
scf1580	19,002-21,687	?	?	i	?	0	ENSDARP00000039867	5E-73	73
scf1591	275,330-276,329	**N**	i	**N**	**N**	1,344	ENSDARP00000153354	0.0	51
scf1592	291,450-292,449	?	**I**	**I**	**I**	9,920			
scf1592	292,897-293,897	i	**I**	**I**	**I**	11,367			
scf1592	1,039,987-1,040,987	?	?	**T**	?	2,081			
scf1593	903,340-904,340	t	**N**	**N**	**N**	13,975			
scf1594	917,356-919,881	i	?	?	?	10,354			
scf1598	580,233-581,233	**N**	**N**	t	**N**	3,497			
scf1598	620,326-621,326	**N**	i	n	**N**	35,058			
scf1598	626,225-627,225	**N**	i	n	**N**	40,957			
scf1598	1,375,535-1,377,190	?	?	t	t	2,311			
<u>scf1605</u>	<u>162,000-163,000</u>	<u>**N**</u>	<u>**N**</u>	<u>**N**</u>	<u>**I**</u>	<u>66,161</u>			
scf1615	468,887-469,887	**N**	**I**	**N**	?	12,139			
scf1616	117,750-118,753	**N**	?	?	**I**	0	No match	NA	NA
scf1616	8,944,364-8,945,364	**N**	**N**	i	**N**	15,866			
scf1634	885,970-892,883	?	?	t	t	0	ENSDARP00000040755	2E-79	79
scf1634	3,578,160-3,579,160	n	n	n	i	0	ENSDARP00000107046	0.0	91
scf1636	1,035,824-1,036,824	n	**N**	t	**N**	45,174			
scf1638	287,078-288,077	?	t	**T**	?	1,364	ENSDARP00000134707	2E-74	80
scf1653	130,788-131,788	**N**	**N**	i	**N**	62,264			
scf1660	338,184-346,736	t	?	?	?	12,303			
scf1675	9,646-10,646	t	n	**N**	**N**	9,259			
scf1677	2,312,527-2,313,526	n	**I**	n	n	31,718			
scf1684	1,014,151-1,015,151	**N**	n	n	**I**	2,896			
scf1684	1,016,710-1,017,710	n	n	n	**I**	4,625			
scf1684	1,643,127-1,644,127	**T**	**N**	**N**	**N**	113,361			
scf1718	38,239-39,239	t	?	?	?	2,348			

Supplementary Table 11 (continued):

Scaffold	Region (bp)	jap	mar	meg	obs	Dist. (bp)	Protein	e	p_{ident}
scf1724	240,561-244,676	i	?	?	?	15,134			
scf1725	36,226-37,225	?	n	?	i	0	ENSDARP00000138648	2E-67	42
scf1744	244,216-245,215	?	?	**T**	?	10,064			
scf1750	84,724-85,723	**T**	**T**	**T**	**T**	5,245			
scf1757	190,802-191,801	**I**	**N**	**N**	**N**	55,322			
scf1776	1,548,541-1,549,541	?	t	?	t	1,334	ENSDARP00000128035	3E-99	60
scf1820	141,030-144,957	t	?	?	?	8,083			
scf1860	549,179-550,178	i	?	?	?	2,967			
scf1860	3,344,167-3,345,166	**N**	**N**	**N**	i	89,950			
scf1884	339,733-340,733	**N**	t	**N**	**N**	16,068			
scf1891	218,847-219,846	?	**N**	**T**	**N**	2,232			
scf1900	124,069-125,068	i	?	?	?	0	ENSDARP00000061365	1E-94	68
scf1916	193,790-194,790	?	**N**	**N**	i	19,992			
scf1922	12,877-16,946	?	t	?	?	21,579			
scf1940	76,099-78,717	?	i	?	?	12,064			
scf1940	79,341-80,341	**I**	?	**I**	**I**	10,440			
scf1953	613,118-614,118	i	**N**	**N**	?	3,540			
scf1953	615,022-617,968	i	?	?	?	5,444			
scf2010	315,783-316,782	i	**N**	**N**	**N**	50,868			
scf2024	222,780-223,815	?	i	i	i	6,940			
scf2024	224,374-225,374	?	**I**	**I**	**I**	8,534			
scf2026	108,009-109,300	i	i	i	?	4,528			
scf2033	180,250-189,823	i	?	?	?	912	ENSDARP00000125758	8E-78	77
scf2047	1,795,251-1,796,368	t	**N**	**N**	**N**	2,671			
scf2066	1,050,648-1,051,647	**N**	?	**N**	t	0	ENSDARP00000138825	0.0	77
scf2070	1,418-2,418	**N**	**N**	**N**	**I**	1,682	ENSDARP00000120732	1E-18	41
scf2070	1,507,665-1,508,665	?	t	**N**	?	2,239			
scf2086	663,990-664,990	?	n	t	?	4,753			
scf2091	1,862,051-1,867,889	?	?	i	i	85	ENSDARP00000134352	0.009	42
scf2105	2,913,650-2,914,649	**N**	t	t	t	22,811			
scf2122	287,068-288,068	**N**	i	i	**N**	21,102			
scf2124	346,963-347,962	t	?	?	?	1,563	ENSDARP00000009902	6E-80	65
scf2138	8,551-9,551	**N**	**N**	**N**	t	4,618			
scf2139	651,171-659,077	?	?	?	t	14,235			
scf2139	702,270-703,269	t	**N**	?	?	23,193			
scf2139	850,183-851,183	t	**N**	?	**N**	1,390	ENSDARP00000133945	2E-62	80
scf2139	854,888-855,888	?	?	t	t	1,953	ENSDARP00000133945	2E-62	80
scf2158	84,900-85,900	**N**	t	?	**N**	0	ENSDARP00000114313	6E-82	63
scf2163	30,886-35,811	?	?	?	i	1,470	ENSDARP00000132945	0.0	57
scf2168	24,880-29,201	t	t	?	?	4,869			
scf2173	198,822-199,821	**N**	**N**	**N**	t	739	ENSDARP00000127227	3E-89	46
scf2173	1,648,302-1,651,613	?	?	t	?	4,676			
scf2173	1,676,372-1,681,635	**N**	?	t	**N**	0	ENSDARP00000062792	0.0	49
scf2177	19,207-20,482	**N**	?	t	?	8,289			
scf2193	246,990-248,371	i	?	?	?	7,730			
scf2199	1,909,399-1,910,479	?	**N**	**N**	t	7,401			
scf2218	567,732-571,325	?	t	?	?	0	ENSDARP00000125370	9E-79	42

Supplementary Table 11 (continued):

Scaffold	Region (bp)	jap	mar	meg	obs	Dist. (bp)	Protein	e	p_{ident}
scf2220	8,810-9,810	t	**T**	t	t	NA			
scf2220	50,765-51,764	t	?	t	t	NA			
scf2227	385,869-386,869	**T**	**N**	**N**	**N**	6,290			
scf2227	1,530,152-1,532,577	i	?	?	?	8,709			
scf2238	200,716-206,285	?	?	?	i	0	No match	NA	NA
scf2254	240,538-241,538	**I**	?	**N**	**N**	0	ENSDARP00000044079	1E-28	46
scf2257	97,083-98,082	?	?	**N**	i	12,284			
scf2257	99,113-100,714	?	?	n	i	14,314			
scf2257	228,812-229,812	t	**N**	?	**N**	15,012			
scf2257	241,987-242,987	t	?	?	t	28,187			
scf2257	247,501-248,988	?	?	t	t	33,701			
scf2257	300,788-301,788	t	t	?	?	86,988			
scf2261	1,456,068-1,457,067	**N**	t	?	n	12,393			
scf2263	142,260-143,260	**T**	**N**	**N**	**N**	5,223			
scf2269	230,043-231,833	**N**	n	i	n	0	ENSDARP00000133844	9E-74	51
scf2269	560,349-561,348	t	?	?	?	5,374			
scf2269	562,757-564,221	?	**T**	?	?	2,501			
scf2273	845,743-846,743	**N**	**N**	**I**	**N**	0	ENSDARP00000106440	0.0	78
scf2273	870,426-872,991	**N**	t	?	**N**	0	ENSDARP00000088845	4E-135	69
scf2285	56,599-59,195	?	t	?	?	25,236			

Supplementary Table 12: *myhc4* exon regions in the *A. megastoma* assembly.
TBLASTX matches between exon sequences of the zebrafish (*Danio rerio*) *myhc4* gene (NCBI accession NM_001020485) and *A. megastoma* scaffold scf7180010919884. *e*-values and percentages of identical amino acids (p_{ident}) are reported for the best match returned in each TBLASTX search.

Exon	e	p_{ident}	Exon sequence (bp)	scf7180010919884 (bp)
2	7E-28	91.11	10-144	20,560-20,426
3	3E-31	90.38	1-156	20,013-19,858
5	2E-17	81.58	2-115	19,213-19,100
6	7E-16	90.32	1-93	19,018-18,926
7	3E-11	100.00	1-63	18,713-18,651
8	2E-17	90.62	3-98	18,535-18,440
9	8E-18	91.18	3-104	18,209-18,108
10	4E-24	82.61	1-138	18,003-17,866
11	1E-20	84.62	3-119	17,356-17,240
12	2E-26	86.00	1-150	16,200-16,051
13	5E-37	96.49	1-171	15,847-15,677
14	6E-60	91.58	1-285	15,589-15,305
16	2E-17	96.55	1-87	14,813-14,727
17	2E-21	89.47	3-116	14,544-14,431
18	8E-21	80.49	2-124	14,313-14,191
19	3E-20	83.33	1-126	14,052-13,927
20	2E-29	80.39	2-154	13,780-13,628
21	4E-34	87.65	1-243	13,106-12,864
22	2E-27	83.05	1-177	12,771-12,595
23	1E-25	93.75	1-144	12,504-12,361
24	9E-13	86.21	2-88	12,260-12,174
25	1E-53	93.75	55-390	12,029-11,694
26	6E-17	72.50	7-126	11,592-11,473
27	7E-18	84.62	3-119	11,372-11,256
28	3E-21	96.67	106-195	10,800-10,711
29	1E-31	90.16	2-184	10,557-10,375
30	6E-29	89.09	1-165	9,943-9,779
32	3E-55	86.27	4-309	9,419-9,114
33	7E-38	92.65	1-204	9,002-8,799
35	7E-31	89.47	1-171	8,045-7,875
36	4E-16	77.14	1-105	218-322
37	1E-14	87.10	4-96	481-573
38	9E-20	87.80	1-123	5,750-5,628

Supplementary Table 13: Nuclear protein-coding sites fixed between *A. marmorata* and *A. megastoma*.

Sites fixed between *A. marmorata* and *A. megastoma* that lie within coding regions of the *A. anguilla* assembly according to the gene prediction with AUGUSTUS. All remainig of the 302 sites determined to be fixed between *A. marmorata* and *A. megastoma* (see Supplementary Figure 9) are located outside of coding regions. Homologous proteins of the zebrafish (*Danio rerio*) proteome were identified with BLASTX [25]; the protein IDs, *e*-values, and percentages of identical amino acids (p_{ident}) are reported for the best match returned in each BLASTX search. We further list the amino-acid (AA) positions at which the nucleotide change occurred as well as the corresponding codon triplet in the *A. anguilla* reference genome asssembly and the new *A. marmorata* and *A. megastoma* assemblies. Non-synonymous substitutions between *A. marmorata* and *A. megastoma* are underlined. With the exception of two non-synonymous substitutions in coding regions for which no zebrafish homolog could be identified, the only non-synonymous substitution in a gene with an identified homolog is located on scaffold scf1929 of the *A. anguilla* assembly. The homologous protein (ENSDARP00000105577) is titin.

Scaffold	Position (bp)	Protein	e	p_{ident}	AA	ang	mar	meg
scf0045	59,074	No match	NA	NA	126	AAA	GAA (Glu)	AAA (Lys)
scf1009	707,494	ENSDARP00000101899	4E-77	63	120	ATC	ATT (Ile)	ATC (Ile)
scf1167	1,209,322	ENSDARP00000139662	4E-148	54	264	GGC	GGC (Gly)	GGT (Gly)
scf1262	557,285	No match	NA	NA	65	GGC	AGC (Ser)	GGC (Gly)
scf1486	2,815,425	ENSDARP00000152243	2E-129	62	173	GTG	GTT (Val)	GTG (Val)
scf1900	1,824,062	ENSDARP00000129749	0	63	729	AAA	AAG (Lys)	AAA (Lys)
scf1929	31,226	ENSDARP00000105577	0	68	5,732	GAC	GGC (Gly)	GAC (Asp)
scf2040	354,121	ENSDARP00000076500	9E-98	50	129	GAG	GAG (Glu)	GAA (Glu)
scf2211	739,277	ENSDARP00000065858	1E-161	98	38	TCT	TCT (Ser)	TCA (Ser)

Supplementary Table 14: *ttna* exon regions in the *A. anguilla* assembly.
TBLASTX matches between exon sequences 160-199 of the zebrafish (*Danio rerio*) *ttna* gene (NCBI accession DQ649453) and *A. anguilla* scaffold scf1929. *e*-values and percentages of identical amino acids (p_{ident}) are reported for the best match returned in each TBLASTX search.

Exon	e	p_{ident}	Exon sequence (bp)	scf1929 (bp)
160	2E-49	70.59	3-308	52,399-52,094
161	7E-24	61.02	6-182	52,004-51,828
163	1E-52	80.81	3-299	51,495-51,199
164	2E-65	90.48	3-317	50,599-50,285
165	1E-42	63.27	3-296	50,192-49,899
166	1E-55	82.83	3-299	49,728-49,432
167	2E-28	87.76	3-149	48,228-48,082
168	2E-26	89.80	4-150	47,976-47,830
169	2E-48	82.61	6-281	47,739-47,464
170	8E-59	84.00	3-302	47,358-47,059
171	3E-47	71.93	3-173	46,907-46,737
172	2E-38	62.37	3-281	46,515-46,237
173	1E-58	80.81	3-299	45,738-45,442
174	4E-55	74.00	3-302	45,343-45,044
175	5E-46	71.29	3-305	44,932-44,630
176	4E-46	74.19	3-281	44,527-44,249
177	1E-55	85.86	3-299	43,795-43,499
178	6E-66	95.88	3-293	43,334-43,044
179	0.0	73.23	1,023-2,210	41,923-40,736
180	2E-51	82.46	162-332	39,735-39,565
181	3E-53	86.02	6-284	39,432-39,154
182	2E-55	81.63	3-296	38,743-38,450
183	7E-55	77.00	3-302	38,357-38,058
184	1E-41	72.53	3-275	37,939-37,667
185	7E-45	71.15	3-158	37,570-37,415
186	2E-50	74.75	6-302	37,140-36,844
187	1E-46	69.70	3-299	36,739-36,443
188	3E-42	70.53	3-287	36,342-36,058
189	2E-60	85.00	3-302	35,938-35,639
190	5E-57	82.00	3-302	31,799-31,500
191	1E-60	84.16	3-305	31,401-31,099
192	1E-51	83.16	3-287	30,929-30,645
193	3E-52	81.25	3-290	30,501-30,214
194	4E-40	62.11	3-287	29,988-29,704
195	1E-102	75.26	6-587	28,747-28,166
196	1E-31	76.92	3-197	26,915-26,721
197	1E-54	81.63	3-296	26,626-26,333
198	2E-96	84.10	3-587	26,246-25,662
199	5E-56	82.83	3-299	25,573-25,277

Supplementary Table 15: Non-synonymous substitutions in mitochondrial genes between *A. marmorata* and *A. megastoma*.
Mitochondrial gene sequences were extracted from the newly generated mitochondrial genome assemblies for *A. marmorata* (mar) and *A. megastoma* (meg) and compared to sequences from the *A. anguilla* (ang) mitochondrial genome (NCBI accession NC_006531).

Gene	AA	ang	mar	meg
nd1	172	ATC (Ile)	ACC (Thr)	ATC (Ile)
nd1	316	ATG (Met)	ATA (Met)	GTG (Val)
nd2	2	AAC (Asn)	AGC (Ser)	AAC (Asn)
nd2	73	ACA (Thr)	ACA (Thr)	TCA (Ser)
nd2	85	GAA (Glu)	GAC (Asp)	GAA (Glu)
nd2	93	ATA (Met)	ACA (Thr)	ATA (Met)
nd2	140	GCC (Ala)	ACC (Thr)	GCC (Ala)
nd2	156	ACA (Thr)	ACA (Thr)	ATA (Met)
nd2	159	GTA (Val)	TTA (Leu)	GCA (Ala)
nd2	285	ACA (Thr)	GCA (Ala)	ACA (Thr)
nd2	331	GTC (Val)	ATC (Ile)	ATA (Met)
nd2	332	CTA (Leu)	ATA (Met)	CTA (Leu)
nd2	335	ATA (Met)	ATA (Met)	ACA (Thr)
co1	155	ATT (Ile)	ATT (Ile)	GTT (Val)
co1	332	ATC (Ile)	ATC (Ile)	GTC (Val)
atp6	25	ACC (Thr)	ATC (Ile)	ACC (Thr)
atp6	40	AAT (Asn)	AAT (Asn)	AGT (Ser)
atp6	48	AGC (Ser)	GGC (Gly)	AGC (Ser)
atp6	52	GGC (Gly)	AAC (Asn)	GGC (Gly)
atp6	71	GTT (Val)	GTT (Val)	ATT (Ile)
atp6	72	ATA (Met)	ATC (Ile)	ATA (Met)
atp6	81	CTA (Leu)	ATT (Ile)	TTA (Leu)
atp6	85	CTG (Leu)	ATA (Met)	CTC (Leu)
atp6	101	CTA (Leu)	ATA (Met)	CTC (Leu)
atp6	112	GCC (Ala)	GCC (Ala)	TCC (Ser)
atp6	116	ATT (Ile)	GTC (Val)	ATC (Ile)
atp6	179	GTC (Val)	GCC (Ala)	GTC (Val)
atp6	185	ATA (Met)	ATA (Met)	ATT (Ile)
atp6	194	GCA (Ala)	TCA (Ser)	GCA (Ala)
atp6	198	TTT (Phe)	CTT (Leu)	TTT (Phe)
atp8	15	TTC (Phe)	CTC (Leu)	TTC (Phe)
atp8	45	CCA (Pro)	CCA (Pro)	TCA (Ser)
co3	175	TTC (Phe)	TTC (Phe)	CTC (Leu)
nd3	13	ACC (Thr)	ACC (Thr)	GCT (Ala)
nd3	84	AAC (Asn)	GAC (Asp)	AAT (Asn)
nd3	87	AAT (Asn)	AAC (Asn)	CAC (His)
nd3	88	GCA (Ala)	ACA (Thr)	GCA (Ala)
nd4l	55	GTA (Val)	ATA (Met)	GTA (Val)

Supplementary Table 15 (continued): Non-synonymous substitutions in mitochondrial genes between *A. marmorata* and *A. megastoma*.

Gene	AA	ang	mar	meg
nd4	18	GTA (Val)	GTA (Val)	ACA (Thr)
nd4	19	AAC (Asn)	AAT (Asn)	GAC (Asp)
nd4	27	ACT (Thr)	ACT (Thr)	ATT (Ile)
nd4	40	GTA (Val)	ATA (Met)	GCA (Ala)
nd4	57	CTA (Leu)	CTA (Leu)	ATA (Met)
nd4	249	ATC (Ile)	ACT (Thr)	ATT (Ile)
nd4	422	AAT (Asn)	AGT (Ser)	AAC (Asn)
nd4	426	GGT (Gly)	GGT (Gly)	GCT (Ala)
nd5	32	ATA (Met)	ATA (Met)	GTA (Val)
nd5	47	ATA (Met)	ATA (Met)	ACA (Thr)
nd5	74	GCT (Ala)	GCT (Ala)	ACT (Thr)
nd5	81	TTA (Leu)	TTA (Leu)	ATA (Met)
nd5	121	CGA (Arg)	CAA (Gln)	CGA (Arg)
nd5	206	GTA (Val)	GTA (Val)	TTA (Leu)
nd5	220	ATA (Met)	ATA (Met)	ATT (Ile)
nd5	224	GTT (Val)	ATT (Ile)	GTT (Val)
nd5	374	TTT (Phe)	TTC (Phe)	TTA (Leu)
nd5	501	GCC (Ala)	GCT (Ala)	GTT (Val)
nd5	505	TTT (Phe)	TTT (Phe)	CTT (Leu)
nd5	506	ACT (Thr)	GCC (Ala)	ACC (Thr)
nd5	540	GTC (Val)	ATC (Ile)	GTC (Val)
nd5	555	GTA (Val)	GTA (Val)	GCA (Ala)
nd6	96	TTT (Phe)	TTT (Phe)	CTT (Leu)
nd6	99	ATT (Ile)	GTT (Val)	ATC (Ile)
nd6	104	GTA (Val)	ATA (Met)	GTA (Val)
nd6	106	TAT (Tyr)	TAT (Tyr)	AGT (Ser)
nd6	146	GTT (Val)	ATT (Ile)	GTT (Val)
cyb	46	CTT (Leu)	ATC (Ile)	GTT (Val)
cyb	369	GTC (Val)	GTA (Val)	GCA (Ala)

Supplementary References

1. Patterson, N., Price, A. L. & Reich, D. Population structure and eigenanalysis. *PLoS Genet.* **2,** e190 (2006).

2. Ishikawa, S., Tsukamoto, K. & Nishida, M. Genetic evidence for multiple geographic populations of the giant mottled eel *Anguilla marmorata* in the Pacific and Indian oceans. *Ichthyol. Res.* **51,** 343–353 (2004).

3. Minegishi, Y., Aoyama, J. & Tsukamoto, K. Multiple population structure of the giant mottled eel, Anguilla marmorata. *Mol. Ecol.* **17,** 3109–3122 (2008).

4. Watanabe, S. *et al.* Evidence of population structure in the giant mottled eel, *Anguilla marmorata*, using total number of vertebrae. *Copeia* **2008,** 680–688 (2008).
5. Gagnaire, P.-A. *et al.* Within-population structure highlighted by differential introgression across semipermeable barriers to gene flow in *Anguilla marmorata. Evolution* **65,** 3413–3427 (2011).
6. Schabetsberger, R. *et al.* Genetic and migratory evidence for sympatric spawning of tropical Pacific eels from Vanuatu. *Mar. Ecol. Prog. Ser.* **521,** 171–187 (2015).
7. Schabetsberger, R. *et al.* Hydrographic features of anguillid spawning areas: potential signposts for migrating eels. *Mar. Ecol. Prog. Ser.* **554,** 141–155 (2016).
8. Peterson, B. K., Weber, J. N., Kay, E. H., Fisher, H. S. & Hoekstra, H. E. Double digest RADseq: An inexpensive method for de novo SNP discovery and genotyping in model and non-model species. *PLoS ONE* **7,** e37135 (2012).
9. Rohland, N. & Reich, D. Cost-effective, high-throughput DNA sequencing libraries for multiplexed target capture. *Genome Res.* **22,** 939–946 (2012).
10. Kircher, M., Sawyer, S. & Meyer, M. Double indexing overcomes inaccuracies in multiplex sequencing on the Illumina platform. *Nucleic Acids Res.* **40,** e3–e3 (2012).
11. Sinha, R. *et al.* Index switching causes "spreading-of-signal" among multiplexed samples in Illumina HiSeq 4000 DNA sequencing. *bioRxiv.* doi:10.1101/125724 (2018).
12. Jacobsen, M. W. *et al.* Speciation and demographic history of Atlantic eels (*Anguilla anguilla* and *A. rostrata*) revealed by mitogenome sequencing. *Heredity* **113,** 432–442 (2014).
13. Patterson, C. in *The fossil record 2* 621–656 (Chapman & Hall, London, UK, 1993).
14. Carnevale, G., Bannikov, A. F., Marramà, G., Tyler, J. C. & Zorzin, R. in *The Bolca Fossil-Lagerstätten: A window into the Eocene World* (eds Papazzoni, C. A. *et al.*) 37–63 (Società Paleontologica Italiana, 2014).
15. Benton, M. J. *et al.* Constraints on the timescale of animal evolutionary history. *Palaeontologia Electronica* **18.1.1FC,** 1–106 (2015).
16. Matschiner, M. *et al.* Bayesian phylogenetic estimation of clade ages supports trans-Atlantic dispersal of cichlid fishes. *Syst. Biol.* **66,** 3–22 (2017).
17. Dela Pierre, F. *et al.* The record of the Messinian salinity crisis in the Tertiary Piedmont Basin (NW Italy): The Alba section revisited. *Palaeogeogr. Palaeoclimatol. Palaeoecol.* **310,** 238–255 (2011).
18. Musilova, Z. *et al.* Vision using multiple distinct rod opsins in deep-sea fishes. *Science* **364,** 588–592 (2019).
19. Rabosky, D. L. *et al.* An inverse latitudinal gradient in speciation rate for marine fishes. *Nature* **559,** 392–395 (2018).
20. Henkel, C. V. *et al.* Primitive duplicate Hox clusters in the European eel's genome. *PLoS ONE* **7,** e32231 (2012).

21. Henkel, C. V. *et al.* First draft genome sequence of the Japanese eel, *Anguilla japonica. Gene* **511,** 195–201 (2012).

22. Bouckaert, R. R. *et al.* BEAST 2.5: An advanced software platform for Bayesian evolutionary analysis. *PLoS Comput. Biol.* **15,** e1006650 (2019).

23. Tavaré, S. Some probabilistic and statistical problems in the analysis of DNA sequences. *Lectures on Mathematics in the Life Sciences* **17,** 57–86 (1986).

24. Yule, G. U. A mathematical theory of evolution, based on the conclusions of Dr. J. C. Willis, F.R.S. *Phil. Trans. R. Soc. B* **213,** 21–87 (1925).

25. Altschul, S. F., Gish, W., Miller, W., Myers, E. W. & Lipman, D. J. Basic local alignment search tool. *Journal of Molecular Biology* **215,** 403–410 (1990).

26. Hasegawa, M., Kishino, H. & Yano, T. Dating of the human-ape splitting by a molecular clock of mitochondrial DNA. *Journal of Molecular Evolution* **22,** 160–174 (1985).

27. Harris, R. S. *Improved pairwise alignment of genomic DNA* PhD thesis (Pennsylvania State University, 2007).

28. Jansen, H. J. *et al.* Rapid de novo assembly of the European eel genome from nanopore sequencing reads. *Sci. Rep.* **7,** 7213 (2017).

29. Benson, G. Tandem repeats finder: a program to analyze DNA sequences. *Nucleic Acids Res.* **27,** 573–580 (1999).

30. Kent, J. W. BLAT–The BLAST-like alignment tool. *Genome Res.* **12,** 656–664 (2002).

31. Blanchette, M. *et al.* Aligning multiple genomic sequences with the threaded blockset aligner. *Genome Res.* **14,** 708–715 (2004).

32. Hoff, K. J. & Stanke, M. WebAUGUSTUS–a web service for training AUGUSTUS and predicting genes in eukaryotes. *Nucleic Acids Res.* **41,** W123–W128 (2013).

33. Howe, K. *et al.* The zebrafish reference genome sequence and its relationship to the human genome. *Nature* **496,** 498–505 (2013).

34. Li, H. & Durbin, R. Fast and accurate short read alignment with Burrows-Wheeler transform. *Bioinformatics* **25,** 1754–1760 (2009).

35. Matschiner, M. Fitchi: haplotype genealogy graphs based on the Fitch algorithm. *Bioinformatics* **32,** 1250–1252 (2016).

36. Stamatakis, A. RAxML version 8: a tool for phylogenetic analysis and post-analysis of large phylogenies. *Bioinformatics* **30,** 1312–1313 (2014).

37. Watanabe, S., Miller, M. J., Aoyama, J. & Tsukamoto, K. Morphological and meristic evaluation of the population structure of *Anguilla marmorata* across its range. *J. Fish Biol.* **74,** 2069–2093 (2009).

38. Alexander, D. H., Novembre, J. & Lange, K. Fast model-based estimation of ancestry in unrelated individuals. *Genome Res.* **19,** 1655–1664 (2009).

39. Malinsky, M., Trucchi, E., Lawson, D. J. & Falush, D. RADpainter and fineRADstructure: Population Inference from RADseq Data. *Mol. Biol. Evol.* **35,** 1284–1290 (2018).

40. Lawson, D. J., Hellenthal, G., Myers, S. & Falush, D. Inference of population structure using dense haplotype data. *PLoS Genet.* **8,** e1002453 (2012).

41. Pulido-Santacruz, P., Aleixo, A. & Weir, J. T. Morphologically cryptic Amazonian bird species pairs exhibit strong postzygotic reproductive isolation. *Proc. R. Soc. London B* **285,** 20172081 (Mar. 2018).

42. Nguyen, L.-T., Schmidt, H. A., Von Haeseler, A. & Minh, B. Q. IQ-TREE: A fast and effective stochastic algorithm for estimating maximum-likelihood phylogenies. *Mol. Biol. Evol.* **32,** 268–274 (2015).

43. Minh, B. Q., Hahn, M. W. & Lanfear, R. New methods to calculate concordance factors for phylogenomic datasets. *bioRxiv.* doi:10.1101/487801 (2018).

44. Nielsen, R. & Beaumont, M. A. Statistical inferences in phylogeography. *Mol. Ecol.* **18,** 1034–1047 (2009).

45. Robinson, J. T. *et al.* Integrative genomics viewer. *Nat. Biotech.* **29,** 24–26 (2011).

46. Green, R. E. *et al.* A draft sequence of the Neandertal genome. *Science* **328,** 710–722 (2010).

47. Durand, E. Y., Patterson, N., Reich, D. & Slatkin, M. Testing for ancient admixture between closely related populations. *Mol. Biol. Evol.* **28,** 2239–2252 (2011).

48. Reich, D., Thangaraj, K., Patterson, N., Price, A. L. & Singh, L. Reconstructing Indian population history. *Nature* **461,** 489–494 (2009).

49. Meyer, B. S., Matschiner, M. & Salzburger, W. Disentangling incomplete lineage sorting and introgression to refine species-tree estimates for Lake Tanganyika cichlid fishes. *Syst. Biol.* **66,** 531–550 (2017).
