# Supplementary material for: Stable species boundaries despite ten million years of hybridization in tropical eels
Source: Nat Commun. 2020 Mar 18;11:1433. doi: 10.1038/s41467-020-15099-x (PMC7080837; doi:10.1038/s41467-020-15099-x)
Supplement: Supplementary file 3 — Reporting Summary [file 41467_2020_15099_MOESM3_ESM.pdf]

nature research

Corresponding author(s): Michael Matschiner, Robert Jehle, Robert Schabetsberger

Last updated by author(s): Feb 5, 2020

Reporting Summary

Nature Research wishes to improve the reproducibility of the work that we publish. This form provides structure for consistency and transparency in reporting. For further information on Nature Research policies, see Authors & Referees and the Editorial Policy Checklist .

Statistics

For all statistical analyses, confirm that the following items are present in the figure legend, table legend, main text, or Methods section.

n/a	Confirmed	
☐	☒	The exact sample size (n) for each experimental group/condition, given as a discrete number and unit of measurement
☐	☒	A statement on whether measurements were taken from distinct samples or whether the same sample was measured repeatedly
☐	☒	The statistical test(s) used AND whether they are one- or two-sided<br>*Only common tests should be described solely by name; describe more complex techniques in the Methods section.*
☒	☐	A description of all covariates tested
☒	☐	A description of any assumptions or corrections, such as tests of normality and adjustment for multiple comparisons
☐	☒	A full description of the statistical parameters including central tendency (e.g. means) or other basic estimates (e.g. regression coefficient) AND variation (e.g. standard deviation) or associated estimates of uncertainty (e.g. confidence intervals)
☐	☒	For null hypothesis testing, the test statistic (e.g. F, t, r) with confidence intervals, effect sizes, degrees of freedom and P value noted<br>*Give P values as exact values whenever suitable.*
☐	☒	For Bayesian analysis, information on the choice of priors and Markov chain Monte Carlo settings
☒	☐	For hierarchical and complex designs, identification of the appropriate level for tests and full reporting of outcomes
☒	☐	Estimates of effect sizes (e.g. Cohen's d, Pearson's r), indicating how they were calculated

Our web collection on statistics for biologists contains articles on many of the points above.

Software and code

Policy information about availability of computer code

Data collection	No particular software was used for data collection. Raw sequencing reads were provided by the sequencing company (Macrogen, Korea).
Data analysis	Read processing and variant calling: STACKS v.2.0-beta9, v.2.2, and GATK v.3.4.64; read mapping: BWA MEM v.0.7.12 and v.0.7.17; read sorting: SAMTOOLS v.1.4, PICARD-TOOLS v.2.6.0; read coverage calculation: BEDTOOLS v.2.26.0; DNA sequence comparisons: BLAST v.2.7.1; variant filtering: BCFTOOLS v.1.6, VCFTOOLS v.0.1.14, PLINK v.1.9; genome assembly: Celera Assembler software v.8.3 (downloaded from the CVS Concurrent Version System repository on 21 June 2017), Pilon v.1.22, BUSCO v.3.0.1; data format conversions: SAMTOOLS v.1.3, BCFTOOLS v.1.6, SE-QTK v.1.0; sequence alignment: MAFFT v.7.397; phylogenetic reconstructions: RAxML v.8.2.11, Fitchi v.1.1.4, BEAST 2 v.2.5.0, SNAPP v.1.3, IQ-TREE v.1.7-beta12; Markov-chain Monte Carlo convergence assessment: Tracer v.1.7; principal component analyses: EIGENSOFT v.6.0.1, JMP v.7.0; model-based population clustering: ADMIXTURE v.1.3, fineRADstructure v.0.3.1, fineSTRUCTURE v.4; past introgression: F4 program v.0.92, fastsimcoal v.2.5.2; demographic analyses: PSMC v.0.6.4-r33; mitochondrial genome assembly: MITObim v.1.8, MIRA v.4.0.2; whole-genome alignment: Tandem Repeat Finder v.4.07b, RepeatMasker v.1.0.8, LASTZ v.1.0.4, MULTIZ-TBA v.012109; gene prediction: AUGUSTUS v.3.3.3. Custom code for computational analyses is available from Github (http://github.com/mmatschiner/anguilla).

For manuscripts utilizing custom algorithms or software that are central to the research but not yet described in published literature, software must be made available to editors/reviewers. We strongly encourage code deposition in a community repository (e.g. GitHub). See the Nature Research guidelines for submitting code & software for further information.

Data

Policy information about availability of data

All manuscripts must include a data availability statement. This statement should provide the following information, where applicable:

- Accession codes, unique identifiers, or web links for publicly available datasets
- A list of figures that have associated raw data
- A description of any restrictions on data availability

The raw RADseq data are deposited on the NCBI SRA database with project number PRJNA590038. Genome assemblies and WGS reads for A. marmorata, A. megastoma, and A. obscura are deposited on ENA with project number PRJEB32187. Haplotype files, alignment files, SNP datasets in VCF format, and input and output of phylogenetic analyses are available from the associated Dryad repository (https://doi.org/10.5061/dryad.ncjsxksr1). Previously available datasets used in this study include the NCBI accessions CM002536, NC_006531, GCA_000002035.4, GCA_000695075, GCA_000470695, NM_001020485, and DQ649453. The source data underlying Figs. 1b-c, 2a-d, i-l, 3c-d, and Supplementary Figs. 3-5, 7, and 15-18 are provided as a Source Data file.

Field-specific reporting

Please select the one below that is the best fit for your research. If you are not sure, read the appropriate sections before making your selection.

☐ Life sciences ☐ Behavioural & social sciences ☒ Ecological, evolutionary & environmental sciences

For a reference copy of the document with all sections, see nature.com/documents/nr-reporting-summary-flat.pdf

Ecological, evolutionary & environmental sciences study design

All studies must disclose on these points even when the disclosure is negative.

Study description	Our study analyzes species relationships, hybridization, and introgression among tropical eel species, based on genomic and morphological data collected in the field.
Research sample	Our sampling covers 7 species of tropical eels (genus Anguilla) from various locations in the Indo-Pacific. The sampled species are particularly suitable for a study of hybridization and introgression as earlier studies have shown evidence of these processes in some species pairs as well as overlapping spawning areas that could promote hybridization (Schabetsberger et al. 2015). Of the seven species, we collected tissue samples from a total of 456 individuals (325 Anguilla marmorata, 41 A. megastoma, 36 A. obscura, 20 A. luzonensis, 4 A. bicolor, 3 A. interioris, and 1 A. mossambica) and morphological measurements from 161 individuals (100 A. marmorata, 30 A. megastoma, 30 A. obscura, 1 A. interioris).
Sampling strategy	Sample sizes for population genomic analyses were based on previous successful projects on hybridization and population structure in marine fish. The sample sizes for some species (particularly A. bicolor, A. interioris, and A. mossambica) were also limited by sample availability. Sampling location were chosen depending on known distributions of species and accessibility. Per location, available juvenile and adult individuals were collected in freshwater by electrofishing and handnets. They were then anaesthetized in clove oil or metomidate. Morphometric measurements were measured and small fin-clips were taken from the pectoral fin and stored in 98% ethanol. Glass eels collected in Indonesia, Philippines, and Taiwan were preserved directly in 98% ethanol.
Data collection	Morphometric measurements of 161 individuals were recorded in the field. Total length, distance from lower jaw to anus, to dorsal fin, and to gill opening, as well as length of mouth and length of pectoral fin were measured with a measuring tape to the nearest mm. Horizontal and vertical eye diameter was measured to the nearest 0.1 mm with ruler callipers. Weight measurements were taken on an electronic balance to the nearest 10 g. All eel species were determined through analysis of body proportions and dentition of the upper jaw. Measurements were taken by Chrysa Gubili, Robert Schabetsberger, Eric Feunteun, David Boseto, Olaf Weyl, and Yu-San Han.<br>Genomic data was obtained through RAD sequencing of 456 individuals, performed by Macrogen (Korea) on the Illumina HiSeq 4000 platform. In addition, whole-genome sequencing was performed at the same sequencing center and the same platform for 3 individuals representing A. marmorata, A. megastoma, and A. obscura.
Timing and spatial scale	Samples were collected over a period of 17 years. Sampling localities (with start and stop dates given in parentheses) included South Africa (AFC: 22/03/09-13/04/09), Swaziland (AFS: 10/06/03), Mayotte (MAY: 09/11/03-11/11/03), Réunion (REU: 05/02/01 and 04/11/03), Indonesia (JAV: 06/11), Philippines (PHC: 26/09/08; PHP: 14/02/14), Taiwan (TAI: 05/06/15), Bougainville Island (BOU: 04/04/15-14/04/15), Solomon Islands (SO: 05/02/16, 05/03/16, 05/04/16, 28/04/16, and 24/10/16-31/10/16), Vanuatu (VAG: 17/01/12-02/02/12 and 01/03/13-03/04/13), New Caledonia (NCA: 27/07/16-18/08/16), Samoa (SAW: 27/08/16-02/09/16 and 23/02/17-09/03/17), and American Samoa (SAA: 18/08/16-20/08/16).
Data exclusions	Genomic data of 26 individuals were excluded due to low sequence quality. The threshold of a minimum read number of 600,000 per individual was established after investigating the overall read-number distribution. Details for all individuals (also for the 26 excluded ones) are given in Supplementary Table 1.
Reproducibility	Analyses of species relationships, hybridization, and introgression were repeated with several approaches to verify the reproducibility of the results. Five replicates of the same analyses were performed with the programs ADMIXTURE and SNAPP, and two different settings were tested with the program IQ-TREE; all of these analyses produced consistent results. To enable the reproduction of our result by other researchers, we provide all datasets, analysis code, and input files for certain programs in dedicated online repositories.

Randomization	Samples were non-randomly allocated to species based on morphology identification and confirmed by genomic sequence data. In some analyses, subsets of individuals were selected per species. This selection was based on data completeness , which can be assumed to be independent of other covariates such as sampling location. Random allocation of individuals to species was not appropriate.
Blinding	Morphological and genomic analyses to determine hybrid status of individuals were carried out by different investigators who shared their results with each other only after both analyses were complete; thus, hybrid status was blinded to each investigator.

Did the study involve field work? ☒ Yes ☐ No

Field work, collection and transport

Field conditions	Fish were collected in tropical lakes, rivers and estuaries. Field trips were conducted over the course of 17 years at various times of the year.
Location	Sample collection was conducted at 14 different location across the Indo-Pacific region. All locations, including sampling dates and coordinates are provided in the Supplementary Material.
Access and import/export	All samples were collected in compliance with local, national, and international laws. Collecting permits were issued by Department of Environmental Protection and Conservation (DEPC, Vanuatu, 1 March 2013), Direction de l'environement, New Caledonia, France (Province Nord-60912-1286-2016/JJC and Province Sud-APA_NCPS_2016.004), Department of Marine and Wildlife Resources, American Samoa (2015/010), Ministry of Natural Resources and Environment, Samoa (1041072), Solomon Islands (RP/2014/007), National Research Institute, Papua New Guinea (P6025227 9AUT6307158M1506303), Province of the Eastern Cape, Department of Economic Affairs and Environment South Africa (CR07/14CR, issued on 01/01/2014). Permits for scientific sampling in Réunion Island and Mayotte were delivered by the French authorities. The methods used to capture the glass eels in Taiwan were approved by the Fisheries Agency of Executive Yuan, Taiwan. Glass eel collection in the Philippines and Indonesia does not require permission. Moreover, the experimental procedures all complied with the experimental animal ethics criteria and were approved by the Institutional Animal Care and Use Committee (IACUC) of the National Taiwan University. No species subject to the Convention on International Trade in Endangered Species of Wild Fauna and Flora (CITES) were collected. Animal health import licenses for non-CITES species from outside the European Union were issued by the Department for Environment, Food, and Rural Affairs, UK (import from South Africa: TARP/2016/039, issued on 10/03/2017; import from Solomon Islands, New Caledonia, Samoa, and American Samoa: ITIMP16.0390A, issued on 13/07/2018; import from Solomon Islands: ITIMP16/0390, issued on 23/06/2018; import from Taiwan: ITIMP16.0878, issued on 04/10/2016).
Disturbance	Only small tissue samples were taken and the majority of fish was released less than 1 h after capture.

Reporting for specific materials, systems and methods

We require information from authors about some types of materials, experimental systems and methods used in many studies. Here, indicate whether each material, system or method listed is relevant to your study. If you are not sure if a list item applies to your research, read the appropriate section before selecting a response.

Materials & experimental systems

n/a	Involved in the study	
☒	☐	Antibodies
☒	☐	Eukaryotic cell lines
☒	☐	Palaeontology
☐	☒	Animals and other organisms
☒	☐	Human research participants
☒	☐	Clinical data

Methods

n/a	Involved in the study	
☒	☐	ChIP-seq
☒	☐	Flow cytometry
☒	☐	MRI-based neuroimaging

Animals and other organisms

Policy information about studies involving animals; ARRIVE guidelines recommended for reporting animal research

Laboratory animals	This study did not involve laboratory animals.
Wild animals	Glass eels, elvers and adult individuals of seven eel species (genus Anguilla) were collected in freshwater by electrofishing and/or handnets. Sex was not determined. Further information on samples is provided in the Supplementary Material. Elvers and adults were anaesthetized in clove oil or metomidate. Morphometric parameters were measured and small fin-clips were taken from the pectoral fin and stored in 98% ethanol. Animals were kept in keep-nets until released within the same day at the capture site (except for a few animals tagged with satellite tags). In Vanuatu a total of 49 individuals were sacrificed by local fishermen. After sampling they were given to local families for food. Glass eels from Taiwan, Indonesia and the Philippines were preserved in 98% ethanol immediately after capture.
Field-collected samples	No field-collected animals were studied in the laboratory. All tissue samples were stored in 98% ethanol.

Ethics oversight	The project was approved by the Research, Innovation and Academic Engagement Ethical Approval Panel of the University of Salford (the institution where DNA extraction and library preparation took place, permit number ST15/68). Local governments further approved the sampling protocols.

Note that full information on the approval of the study protocol must also be provided in the manuscript.
